# Supplementary material for: Disease burden of ageing, sex and regional disparities and health resources allocation: a longitudinal analysis of 31 provinces in Mainland China
Source: BMJ Open. 2022 Nov 15;12(11):e064641. doi: 10.1136/bmjopen-2022-064641 (PMC9670959; doi:10.1136/bmjopen-2022-064641)
Supplement: Supplementary data [file bmjopen-2022-064641supp002.pdf]

Appendix

Table S1. Administrative division of provinces in mainland China

Administrative division (No.)	Province
Eastern provinces (11)	Beijing, Fujian, Guangdong, Hainan, Hebei, Jiangsu, Liaoning, Shandong, Shanghai, Tianjin, Zhejiang
Central provinces (8)	Anhui, Heilongjiang, Henan, Hubei, Hunan, Jiangxi, Jilin, Shanxi
Western provinces (12)	Inner Mongolia, Guangxi, Chongqing, Gansu, Guizhou, Ningxia, Qinghai, Shaanxi, Sichuan, Tibet, Xinjiang, Yunnan

Table S2. IHME standard age weight

Age group	Population percentage
<5	7.2%
5–9	8.7%
10–14	8.4%
15–19	8.1%
20–24	7.8%
25–29	7.6%
30–34	7.2%
35–39	6.9%
40–44	6.4%
45–49	5.8%
50–54	5.3%
55–59	4.7%
60–64	4.1%
65–69	3.4%
70 +	8.6%

ARD burden-adjusted age calculation

First, we identified the ARD burden-adjusted age of China by comparing the national average burden rate with its burden rates by age group. We selected the two five-year age groups with the closest burden rate as the national average and calculated the national average age by assuming a linear increase in the ARD burden within each five-year age group. For example, the age-related disease burden of China was 12637.2 DALYs per 100,000 population in 2016, closest to burden rates for the age group of 45–49 (9316.3) and 50–54 (14163.7). Hence, the national age was calculated as: 45+12637.2/((9316.3+14163.7)/10)=50.38.

Chen S, *et al. BMJ Open* 2022; 12:e064641. doi: 10.1136/bmjopen-2022-064641

Second, we calculated the ARD burden-adjusted age of each province by dividing the provincial age-related disease burden rate by the unit share of the national average burden rate per age. The unit share of the national average burden rate per age was calculated by dividing the national average burden rate by the national equivalent age. For example, the age-related disease burden of Shanghai was 8,127·4 DALYs per 100,000 population. Hence, Shanghai's ARD burden-adjusted age was calculated as: 8,127·4/(12637·2/50.38)=32·40.

Regression model

$$Y_{it} = \alpha + \beta_0 healthexp_{it} + \beta_1 healthwf_{it} + \beta_2 X_{it} + \beta_3 D_t + \eta_i + \varepsilon_{it},$$

where Y_{it} is the age-standardised age-related disease burden of Province i in Year t, β_0 is the coefficient of interest, $healthexp_{it}$ is the total health expenditures per capita, and $healthwf_{it}$ is the total health professional density, licensed doctor density, or licensed nurse density (per 1,000 population) of the Province i in Year t (three separate models). X_{it} is the set of covariates controlled in the model, including GDP per capita, education, the proportion of females, the proportion of people living in urban areas, and the urban-rural health workforce density ratio. D_t represents the time dummies, η_i the province fixed effects, and ε_{it} is the error term. All variables are in log form except for the time dummies.

We also assessed the correlation between 1) the total health expenditures per capita and health workforce density and 2) the rural/urban ratio in health workforce density and the proportion of rural/urban population to check multicollinearity before and after controlling for covariates, including GDP per capita, sex and education. The results are presented below in Table S3 and Table S4.

Chen S, *et al. BMJ Open* 2022; 12:e064641. doi: 10.1136/bmjopen-2022-064641

Table S3. Regression model results: assessing correlation between health workforce density and total health expenditures per capita

Independent Variables	Model 1		Model 2		Model 3		Model 4		Model 5		Model 6	
	Coef (β)	P value	Coef (β)	P value	Coef (β)	P value	Coef (β)	P value	Coef (β)	P value	Coef (β)	P value
Health prof density	0.09	0.10	-0.04	0.34	··	··	··	··	··	··	··	··
Licensed doc density	··	··	··	··	0.11	0.06	0.01	0.89	··	··	··	··
Licensed nurse density	··	··	··	··	··	··	··	··	0.08	0.09	-0.02	0.51
GDP per capita	··	··	0.37	<0.01	··	··	0.33	<0.01	··	··	0.36	<0.01
Female (%)	··	··	0.08	0.32	··	··	0.08	0.35	··	··	0.08	0.33
≥ junior mid sch educ (%)	··	··	0.08	0.50	··	··	0.06	0.58	··	··	0.08	0.50
adj. R^2	0.96	··	0.94	··	0.96	··	0.94	··	0.96	··	0.94	··

Note:
Model 1: Total health professional density as independent variable, not controlling for covariates except for time dummies
Model 2: Total health professional density as independent variable, controlling for covariates except for time dummies
Model 3: Licensed doctor density as independent variable, not controlling for covariates except for time dummies
Model 4: Licensed doctor density as independent variable, controlling for covariates except for time dummies
Model 5: Licensed nurse density as independent variable, not controlling for covariates except for time dummies
Model 6: Licensed nurse density as independent variable, controlling for covariates except for time dummies

Table S4. Regression model results: assessing correlation between urban residency and urban-rural ratio in health workforce density

Independent Variables	Model 1		Model 2	
	Coef (β)	P value	Coef (β)	P value
Population living in urban areas (%)	-0.44	0.23	-0.06	0.90
GDP per capita	··	··	-0.01	0.95
Female (%)	··	··	-0.19	0.02
≥ junior mid sch educ (%)	··	··	-0.28	0.11
adj. R^2	0.84	··	0.94	··

Note:
Model 1: Population living in urban areas as independent variable, not controlling for covariates except for time dummies
Model 2: Population living in urban areas as independent variable, controlling for covariates except for time dummies

Chen S, *et al. BMJ Open* 2022; 12:e064641. doi: 10.1136/bmjopen-2022-064641

Table S5. Selected statistics of the variables in the regression model, 2010 and 2016

Variables	Health expenditures per capita (CNY)*		Total health professionals density (per 1,000 population)		Urban-rural ratio in total health professionals density		GDP per capita (CNY)*		Living in urban areas (%)		Female (%)		Received at least middle school education (%)	
Province	2010	2016	2010	2016	2010	2016	2010	2016	2010	2016	2010	2016	2010	2016
Anhui	1210·5	2652·2	3·1	4·7	2·4	2·3	20888·0	39561·0	44.8%	52.0%	49·2%	48·7%	62·8%	65·5%
Beijing	4147·2	9429·7	13·6	10·8	1·9	··	73856·0	118198·0	86.2%	86.5%	48·4%	48·6%	87·6%	88·6%
Chongqing	1501·0	3492·2	3·4	5·9	1·4	2·0	27596·0	58502·0	55.0%	62.6%	49·4%	49·2%	61·0%	64·1%
Fujian	1280·1	3226·8	4·1	5·7	2·8	2·5	40025·0	74707·0	58.1%	63.6%	48·6%	49·1%	63·0%	61·7%
Gansu	1153·9	2889·2	3·7	5·2	2·0	2·2	16113·0	27643·0	37.2%	44.7%	48·9%	49·3%	56·5%	57·9%
Guangdong	1445·9	3812·5	5·3	6·0	3·2	3·2	44736·0	74016·0	66.5%	69.2%	47·8%	46·9%	73·2%	74·3%
Guangxi	1116·9	2557·0	3·6	6·0	2·2	2·2	20219·0	38027·0	41.8%	48.1%	48·0%	48·0%	62·5%	67·4%
Guizhou	946·6	2472·4	2·5	5·8	4·2	3·9	13119·0	33246·0	35.0%	44.2%	48·3%	48·4%	48·8%	54·6%
Hainan	1193·0	3306·8	4·4	6·3	2·3	3·3	23831·0	44347·0	50.5%	56.8%	47·4%	47·3%	72·1%	73·0%
Hebei	1253·8	2710·6	4·0	5·3	3·2	2·7	28668·0	43062·0	45.6%	53.3%	49·3%	48·9%	69·9%	69·7%
Heilongjiang	1580·2	3133·4	5·0	5·8	2·3	2·3	27076·0	40432·0	56.5%	59.2%	49·2%	49·5%	73·0%	73·3%
Henan	1134·0	2594·0	3·5	5·7	3·0	3·2	24446·0	42575·0	40.6%	48.5%	49·5%	49·0%	68·2%	69·8%
Hubei	1191·1	3270·6	4·2	6·5	2·1	2·2	27906·0	55665·0	51.8%	58.1%	48·6%	48·7%	69·8%	70·5%
Hunan	1042·1	2821.0	3·8	5·8	2·9	2·9	24719·0	46382·0	45.1%	52.8%	48·6%	48·9%	67·1%	71·2%

Chen S, *et al. BMJ Open* 2022; 12:e064641. doi: 10.1136/bmjopen-2022-064641

**Inner Mongolia**	1767·5	3599·7	5·1	6·8	2·7	2·6	47347·0	72064·0	56.6%	61.2%	48·1%	49·5%	69·9%	73·3%
**Jiangsu**	1566·0	4200·2	4·4	6·5	2·0	2·1	52840·0	96887·0	61.9%	67.7%	49·6%	49·6%	70·9%	71·5%
**Jiangxi**	992·0	2374·8	5·1	6·1	1·8	2·1	21253·0	40400·0	45.7%	53.1%	48·2%	48·0%	65·6%	64·1%
**Jilin**	1653·9	3501·19	5·5	6·3	2·5	2·9	31599·0	53868·0	53.4%	56.0%	49·3%	49·2%	69·8%	73·6%
**Liaoning**	1765·9	3390·9	4·7	6·6	3·0	2·6	42355·0	50791·0	64.0%	67.4%	49·4%	49·5%	75·5%	78·3%
**Ningxia**	1190·1	3730·5	4·5	6·2	4·4	5·4	26860·0	47194·0	49.8%	56.3%	48·8%	48·4%	61·3%	66·5%
**Qinghai**	1472·0	4043·1	4·7	7·6	2·0	2·0	24115·0	43531·0	46.3%	51.6%	48·2%	48·6%	49·8%	51·8%
**Shaanxi**	2040·7	3535·7	4·7	6·5	1·7	2·2	27133·0	51015·0	47.3%	55.3%	48·3%	49·5%	69·8%	71·5%
**Shandong**	1403·1	3372·7	9·7	7·4	1·3	1·6	41106·0	68733·0	50.9%	59.0%	49·4%	49·0%	67·5%	69·0%
**Shanghai**	2828·1	7596·0	5·6	6·1	2·6	3·2	76074·0	116562·0	89.3%	87.9%	48·5%	48·6%	83·6%	83·5%
**Shanxi**	1297·5	2650·3	3·4	4·8	2·7	3·0	26283·0	35532·0	49.7%	56.2%	48·6%	48·5%	74·1%	77·5%
**Sichuan**	1019·1	3238·6	3·6	6·0	2·0	2·0	21182·0	40003·0	41.8%	49.2%	49·2%	50·1%	57·2%	58·9%
**Tianjin**	2737·3	5294·2	7·1	6·1	1·4	1·1	72994·0	115053·0	80.5%	82.9%	46·6%	46·6%	80·5%	82·0%
**Tibet**	1472·0	3780·9	3·4	4·5	4·9	4·1	17027·0	35184·0	22.8%	29.6%	48·6%	49·5%	26·4%	29·6%
**Xinjiang**	1676·8	4012·9	5·7	7·1	3·2	2·4	25034·0	40564·0	43.5%	48.3%	48·7%	48·9%	66·1%	65·5%
**Yunnan**	1107·2	2754·1	3·2	5·2	3·2	3·3	15752·0	31093·0	36.8%	45.0%	48·1%	49·5%	48·3%	52·5%
**Zhejiang**	2099·0	4603·8	6·1	7·7	1·8	1·8	51711·0	84916·0	62.3%	67.0%	48·6%	47·9%	64·7%	66·0%

*CNY=Chinese Yuan, exchange rate: 1USD≈6.37CNY (Dec 3, 2020)

Data source: National Statistical Yearbook of China (2011, 2017) and National Health Statistical Yearbook of China (2011,2017)

Chen S, *et al. BMJ Open* 2022; 12:e064641. doi: 10.1136/bmjopen-2022-064641
