# Supplementary material for: Disease burden of ageing, sex and regional disparities and health resources allocation: a longitudinal analysis of 31 provinces in Mainland China
Source: BMJ Open. 2022 Nov 15;12(11):e064641. doi: 10.1136/bmjopen-2022-064641 (PMC9670959; doi:10.1136/bmjopen-2022-064641)
Supplement: Author's manuscript [file bmjopen-2022-064641.draft_revisions.pdf]

BMJ Open is committed to open peer review. As part of this commitment we make the peer review history of every article we publish publicly available.

When an article is published we post the peer reviewers' comments and the authors' responses online. We also post the versions of the paper that were used during peer review. These are the versions that the peer review comments apply to.

The versions of the paper that follow are the versions that were submitted during the peer review process. They are not the versions of record or the final published versions. They should not be cited or distributed as the published version of this manuscript.

BMJ Open is an open access journal and the full, final, typeset and author-corrected version of record of the manuscript is available on our site with no access controls, subscription charges or pay-per-view fees (http://bmjopen.bmj.com).

If you have any questions on BMJ Open's open peer review process please email info.bmjopen@bmj.com

BMJ Open

Health Burden of Ageing, Sex and Regional Disparities, and Health Resources Allocation: A Longitudinal Analysis of 31 Provinces in Mainland China

Journal:	*BMJ Open*
Manuscript ID	bmjopen-2022-064641
Article Type:	Original research
Date Submitted by the Author:	11-May-2022

Complete List of Authors:	Chen, Shu; UNSW, School of Risk and Actuarial Studies; UNSW, ARC Centre of Excellence in Population Ageing Research (CEPAR)<br>Si, Yafei; University of New South Wales, School of Risk and Actuarial Studies; University of New South Wales, ARC Centre of Excellence in Population Ageing Research (CEPAR)<br>Hanewald, Katja; University of New South Wales, School of Risk and Actuarial Studies; University of New South Wales, ARC Centre of Excellence in Population Ageing Research (CEPAR)<br>Li, Bingqin; University of New South Wales, Social Policy Research Center<br>Bateman, Hazel; University of New South Wales, School of Risk and Actuarial Studies; University of New South Wales, ARC Centre of Excellence in Population Ageing Research (CEPAR)<br>Dai, Xiaochen; Institute of Health Metrics and Evaluation<br>wu, chenkai; Duke Kunshan University, Global Health Research Center<br>Tang, Shenglan; Duke Kunshan University, Global Health Research Center; Duke University, Duke Global Health Institute

Keywords:	Health policy < HEALTH SERVICES ADMINISTRATION & MANAGEMENT, EPIDEMIOLOGY, Health economics < HEALTH SERVICES ADMINISTRATION & MANAGEMENT, PUBLIC HEALTH

SCHOLARONE™
Manuscripts

I, the Submitting Author has the right to grant and does grant on behalf of all authors of the Work (as defined in the below author licence), an exclusive licence and/or a non-exclusive licence for contributions from authors who are: i) UK Crown employees; ii) where BMJ has agreed a CC-BY licence shall apply, and/or iii) in accordance with the terms applicable for US Federal Government officers or employees acting as part of their official duties; on a worldwide, perpetual, irrevocable, royalty-free basis to BMJ Publishing Group Ltd ("BMJ") its licensees and where the relevant Journal is co-owned by BMJ to the co-owners of the Journal, to publish the Work in this journal and any other BMJ products and to exploit all rights, as set out in our licence.

The Submitting Author accepts and understands that any supply made under these terms is made by BMJ to the Submitting Author unless you are acting as an employee on behalf of your employer or a postgraduate student of an affiliated institution which is paying any applicable article publishing charge ("APC") for Open Access articles. Where the Submitting Author wishes to make the Work available on an Open Access basis (and intends to pay the relevant APC), the terms of reuse of such Open Access shall be governed by a Creative Commons licence – details of these licences and which Creative Commons licence will apply to this Work are set out in our licence referred to above.

Other than as permitted in any relevant BMJ Author's Self Archiving Policies, I confirm this Work has not been accepted for publication elsewhere, is not being considered for publication elsewhere and does not duplicate material already published. I confirm all authors consent to publication of this Work and authorise the granting of this licence.

For peer review only

Health Burden of Ageing, Sex and Regional Disparities, and Health Resources Allocation: A Longitudinal Analysis of 31 Provinces in Mainland China

Title page

Shu Chen MSc [1,2], Yafei Si MA [1,2], Katja Hanewald PhD [1,2], Bingqin Li PhD [3], Hazel Bateman PhD [1,2], Xiaochen Dai MSc, MPH, PhD [4], Chenkai Wu PhD [5], Shenglan Tang MD, PhD * [5,6,7]

1 Australian Research Council Centre of Excellence in Population Ageing Research (CEPAR), University of New South Wales, Sydney, Australia.
2 School of Risk & Actuarial Studies, University of New South Wales, Sydney, Australia.
3 Social Policy Research Centre, University of New South Wales, Sydney, Australia.
4 The Institute of Health Metrics and Evaluation, Seattle, US.
5 Global Health Research Center, Duke Kunshan University, Kunshan, China.
6 Duke Global Health Institute, Duke University, Durham, US.
7 SingHealth Duke-NUS Global Health Institute, Duke-NUS, Singapore.

Corresponding to:

Shenglan Tang, MD, PhD
Email: shenglan.tang@duke.edu

Address:
Duke Global Health Institute
310 Trent Drive, Durham
NC 27710, United States

For peer review only

For peer review only - http://bmjopen.bmj.com/site/about/guidelines.xhtml

Abstract

Objectives
To measure the health burden of ageing based on age-related diseases (ARD), the sex and regional disparities, and the impact of health resources allocation on the burden in China.

Methods
We used the estimates from the Global Burden of Diseases, Injuries, and Risk Factors Study (GBD) and China's routine official statistics for analysis. We first identified the ARDs and calculated the national burden in 2016. We assessed the ARD burden disparities by province and sex, and calculated the provincial ARD burden-adjusted age. We assessed historical changes between 1990 and 2016. Fixed-effects regression models were adopted to assess the impact of health expenditures and human resources indicators on the ARD burden in 2010-2016.

Results
In 2016, China's total burden of ARDs was 15703.7 DALYs (95% UI: 12628.5, 18406.2) per 100,000 population. Non-communicable diseases (NCDs) accounted for 91.9% of the burden. There were significant regional disparities. The leading five youngest provinces were Beijing, Guangdong, Shanghai, Zhejiang and Fujian, located in east coast of China with an ARD burden-adjusted age below 40. After standardising the age structure, western provinces including Tibet, Qinghai, Guizhou, and Xinjiang had the highest burden of ARDs. Males were disproportionately affected by ARDs. China's overall age-standardised ARD burden had decreased since 1990, females and eastern provinces experienced the largest decline. We did not observe burden decline in sense organ diseases and neurological disorders. Regression results showed the urban-rural gap in health workforce density was positively associated with the ARD burdens.

Conclusion
Chronological age alone does not provide a strong enough basis for appropriate ageing resource planning or policymaking. In China, concerted efforts should be made to reduce the ARDs burden and its disparities. Health resources should be deliberately allocated to western provinces which face the greatest health threat due to future ageing.

For peer review only - http://bmjopen.bmj.com/site/about/guidelines.xhtml

Introduction

The world is rapidly ageing due to increased life expectancy and decreased fertility rates.[1] Health is a key factor in determining whether population ageing means more opportunities or challenges to society.[2] Although the global population is living longer, it is unclear whether these extended years of life are spent in good or bad health.[2–9] It is therefore essential to measure the interactions between ageing and health for a clearer understanding of the health burden of ageing. Numerous metrics have been developed to measure population ageing and health for resource planning. Traditionally, the change of chronological age and age structure have been used to assess ageing based on demographic metrics such as life expectancy or the percentage of the population over a certain age threshold.[10] Another set of metrics assesses functional status through indicators such as frailty,[11,12] disability,[13–15] cognitive function,[16] and intrinsic capacity.[17,18] Health status is typically assessed based on biomarkers, self-reported health status[19,20] and epidemiological indicators of different diseases such as incidence, prevalence, mortality, and disability-adjusted life years (DALYs). Some metrics measure ageing while also taking health status into account. These metrics include healthy life years (HLYs), healthy life expectancy (HALE), and biological age. However, these metrics either measure ageing and health separately or cannot provide disease-specific burden information to guide resource planning.

To complement the existing metrics and measure the population-level health burden of ageing, Chang et al. developed a novel metric called the age-related disease (ARD) burden.[21] They defined ARDs as diseases with incidence rates that increased quadratically with age. The researchers used estimates from the Global Burden of Diseases, Injuries, and Risk Factors Study (GBD) 2017 to measure and compare ARD burdens of 195 countries from 1990 to 2017. They found non-communicable diseases (NCDs) were the primary contributor to ARDs, and there were large variations in ARD burdens across countries. Of the 195 countries, Switzerland had the lowest ARD burden, while China ranked 75th and Vanuatu had the highest. The researchers found that the age-standardised ARD burden dropped globally between 1990 and 2017 due to lower disease severity and case fatality rates. Hu et al. assessed China's ARD burden based on the GBD 2017 study estimates at national level. The researchers consistently found that NCDs are the primary contributor to ARDs in China. The age-standardised ARD burdens also decreased between 1990 and 2017, and the magnitude of ARD decrease was larger among women than men.[22] These findings emphasise the inadequacy of using chronological age alone to inform resource planning and policy design. They confirm the importance of considering health status and disease severity within the context of ageing and the need for concerted efforts to address regional disparities, especially for regions or countries with large development inequalities.[21,22]

Many previous studies have assessed whether and how health resource allocation can impact health outcomes.[23–29] Health workforce density and total health expenditures per capita have been widely used as key proxies for health resources. A large body of high-quality evidence supports the view that a higher density of health workforce can help improve health outcomes such as life expectancy, infant mortality rate, under-five mortality rate, and maternal mortality rate.[23,24] Evidence further shows heterogeneity among different types of health workforce in terms of how they impact health outcomes.[23,24] The impact of health expenditures on health outcomes is mixed.[26–29] For example, evidence shows that an increase in health expenditures per capita can positively impact health outcomes, including life expectancy, under-five mortality, and maternal mortality in Sub-Saharan African countries,[29] though a similar study did not find supporting evidence in Organisation for Economic Co-operation and Development (OECD) countries.[26] Another study found a significant association between higher health expenditures per capita and lower infant mortality in 15 European Union (EU) countries, but only marginal increases in life expectancy.[28]

For peer review only - http://bmjopen.bmj.com/site/about/guidelines.xhtml

China is a large, rapidly ageing country with uneven economic and health development across provinces. In 2020, 264 million of China's inhabitants (18.7%) were over 60 years old, ranking first globally by this metric, and this number is projected to double by 2050.[1,30] Generally, in China, the economy is most developed in eastern provinces followed by the central and western provinces. For example, in 2020, the gross domestic product (GDP) per capita in Shanghai, a municipality located on China's east coast, was 4·3 times higher than that of Gansu, a province in western China.[30] Regional disparities in health development are similar in scale; provinces in the eastern region generally perform best, followed by the central and western provinces.[31] For example, among males in 2015, the average healthy life expectancy was estimated to be around 78 years in Beijing, Tianjin, and Shanghai, compared to 69 years in Qinghai, Tibet, and Yunnan located in western China.[32] China is estimated to attain 12 of the 28 health-related Sustainable Development Goals (SDGs) indicators by 2030, and the country's eastern provinces are estimated to achieve a higher number.[33] Therefore, understanding the disparities in health burden of ageing across provinces and the impact of health resource allocation on the burden is crucial for China's central and provincial governments to plan for an ageing society. Nevertheless, no study has yet been conducted for this purpose. To fill the evidence gap, this study aims to: 1) measure the burden of ARDs in China at the subnational level, examining disparities across provinces and by sex, 2) assess changes in ARD burden from 1990–2016, and 3) explore how health resource allocation impacts the ARD burden. We also examined the difference by disease group whenever possible.

Methods

Overview

This study used estimates from the GBD study to calculate and analyse the ARD burden in China. The GBD team have updated their estimates annually since 2015 (except for 2018), with improvements on disease causes, risk factors, data input sources and modelling strategies to obtain updated and robust estimates. The most recent estimates released publicly were GBD 2019.[34,35] We used GBD 2019 study estimates to select ARDs and calculate the total national burden considering its timeliness. We conducted analysis of the ARD burden at subnational level based on estimates of the GBD 2016 study, as they were the most recent subnational estimates of China our team could access. However, we did not find significant changes on the causes and estimates that could impact the robustness of the study findings after careful comparison. The detailed methodology of the GBD 2016 and 2019 studies has been published elsewhere.[35,36] The GBD study classified the causes hierarchies into four levels, each with increased specificity.[37] Level One consists of three general causes: communicable diseases, NCDs, and injuries. Levels Two, Three, and Four further divide these causes into subgroups.[37] The data related to provincial health expenditures, human resources for health, and economic and demographic indicators were obtained from China Statistical and Health Statistical Yearbooks, providing data from 2010 to 2016 for regression analysis.

The geographical units of analysis are provincial-level jurisdictions in mainland China, including 22 provinces, five autonomous regions, and four municipalities (hereafter referred to as 31 provinces).[30] Our study complies with the guidelines for Accurate and Transparent Health Estimates Reporting (known as GATHER) statement and we provided the GATHER checklist in the appendix.

ARD selection and burden measurement

Following the definition developed by Chang et al., ARDs were defined as diseases with incidence rates that increased quadratically with age among the adult population.[21] Our selection focused on GBD Level Three causes, and the detailed selection methods have been published elsewhere.[21] To include a comprehensive list of diseases, we defined the adult population as people aged 15 years and older.

For peer review only - http://bmjopen.bmj.com/site/about/guidelines.xhtml

The ARD burden was measured by calculating the sum of the DALYs of the identified ARDs in the entire population. DALYs are defined as the sum of the years of life lost due to premature mortality and the years lived with a disability due to a disease or health condition. We calculated the provincial ARD burden of non-communicable diseases (NCDs) to approximate the total ARD burden to analyse regional disparities, as previous studies showed NCDs contributed to over 80% of the ARD burden,[21,22] and we could only access the subnational estimates of NCDs. We reported the ARD burden rate, measured as DALYs per 100,000 population, to facilitate comparisons. To explore the burden composition by disease category, the ARD burden was further stratified and analysed at the second level of GBD causes within NCDs and by sex when appropriate. We also calculated the percentage decrease in the ARD burden to assess the historical change and clustered the results by disease category and region for comparative analysis (please see appendix Table S1 for the administrative divisions).

We estimated the ARD burden-adjusted age of each province to facilitate an intuitive assessment and comparison across provinces. We first identified the national ARD burden-adjusted age based on its age-specific burden rate. We selected the two five-year age groups with closest burden rate as the national average, and calculated the national average age by assuming a linear increase of the ARD burden within each five-year age group. Afterwards, the provincial ARD burden-adjusted age was calculated, assuming each province shared the same burden rate per age as the national average (appendix for calculation details). A younger ARD burden-adjusted age, therefore, implied a lower ARD burden.

To compare historical change in the ARD burden, we applied age standardisation to the calculation using the Institute for Health Metrics and Evaluation (IHME) standard population age structure when necessary (appendix Table S2 for the standard population structure). The 95% uncertainty intervals (UI), generated by the GBD study team as the 25th and 975th ordered 1000 random draw values of the posterior distribution,[36,38] were also presented for the ARD burden estimates.

Analysing the impact of health resource allocation on the ARD burden

We explored the impact of health resource allocation on the ARD burden using a panel data analysis approach. All equations were estimated with a log-linear functional form to enable unit-free comparisons of coefficients. To understand the underlying reasons for burden shifts over time, we used the age-standardised ARD burden rate as the dependent variable. Health expenditures and health workforce density were adopted as proxy measures for health resources. Total health expenditure per capita was the key independent variable used to measure health expenditures.[26,27] The key independent variables used to measure health workforce density were three separate sets of indicators: total health professional density, licensed doctor density, and licensed nurse density, all per 1,000 population. Health professionals included licensed doctors (clinical, dental, public health, and traditional Chinese medicine), licensed nurses, pharmacists, clinical laboratory technicians, and radiologists.[39] We chose three sets of indicators to measure human resources for health because 1) together, they accurately represent the distribution of China's health personnel resources; 2) they are widely used in published literature and heterogeneity exists in terms of their impact on health outcomes;[23,24] 3) data stratified by province and by urban and rural areas are available for all three indicators. We ran three separate regression models to include the three health workforce density indicators respectively.

We included GDP per capita, sex, and education as covariates in the regression models to account for the major socio-economic determinants of the population health burden. To account for the large gaps in urban-rural development and health across China, we included the percentage of people residing in urban areas (to measure the process of urbanization) and the ratio of urban-rural health workforce density in

For peer review only - http://bmjopen.bmj.com/site/about/guidelines.xhtml

the model as covariates. The model also included time dummies (to control period effects), province fixed effects and an error term (see appendix for model details). Standard errors were clustered at the provincial level. Province was the unit of analysis, and a fixed-effects estimator was used to remove the potential endogeneity from time-invariant omitted variables.

Since provincial-level data on health expenditures per capita and health workforce density were only largely available from 2010 onwards, our panel dataset included data from 2010 to 2016. We performed log-linear interpolation to obtain annual estimates of the ARD rate from 2010 to 2016 as the GBD study only provides estimates in five-year intervals.[38] The health expenditures per capita in 2010 are missing for seven provinces: Shanghai, Hainan, Sichuan, Tibet, Shaanxi, Qinghai, and Ningxia. Only data of Tibet is missing in 2011. Therefore, after testing the robustness of the linear increase assumption, we imputed the missing data, assuming a linear increase in per capita health expenditures between 2010 and 2016. All analyses were performed in Stata 16·0 (Stata Corp LLP, College Station, TX, USA).

Patient and public involvement statement

Patients or the public were not involved in the design, or conduct, or reporting, or dissemination plans of our research.

Results

A total of 58 causes out of 169 causes (34·3%) at level three were selected as ARDs in China (Table 1). Nine of the 58 causes were infectious diseases (15·5%), 47 were NCDs (81·0%), and two were injuries (3·5%). In 2016, the total burden of ARDs in China was 15703.7 DALYs (95% UI: 12628.5, 18406.2) per 100,000 population. NCDs accounted for 91·9% of the total burden. Among the NCDs, cardiovascular diseases, neoplasms, chronic respiratory diseases, sense organ diseases and neurological disorders were the five leading contributors to the ARD burden.

Table 1. Summary of age-related diseases in China by category

GBD Causes at Level 1	GBD Causes at Level 2	GBD Causes at Level 3
**Infectious diseases**	Respiratory infections and Tuberculosis	Tuberculosis, diarrheal diseases, lower respiratory diseases
	Other infectious diseases	Meningitis, encephalitis, tetanus, varicella, and herpes zoster
	Neglected tropical diseases and malaria	Cysticercosis, trachoma
**NCDs**	Neoplasms	Oesophageal cancer, stomach cancer, liver cancer, tracheal, bronchus, and lung cancer, prostate cancer, colon and rectum cancer, lip and oral cavity cancer, other pharynx cancer, gallbladder and biliary tract cancer, pancreatic cancer, malignant skin melanoma, non-melanoma skin cancer, bladder cancer, brain and central nervous system cancer, mesothelioma, Hodgkin lymphoma, non-Hodgkin lymphoma, multiple myeloma, leukaemia, other malignant neoplasms
	Cardiovascular Diseases	Ischemic heart disease, stroke, hypertensive heart disease, cardiomyopathy, myocarditis, atrial fibrillation and flutter, peripheral artery disease, endocarditis

For peer review only - http://bmjopen.bmj.com/site/about/guidelines.xhtml

	Chronic respiratory diseases	Chronic obstructive pulmonary disease, asthma, and interstitial lung disease
	Digestive Diseases	Paralytic ileus and intestinal obstruct, vascular intestinal disorders, pancreatitis
	Neurological disorders	Alzheimer's disease and other dementias, Parkinson's disease, idiopathic epilepsy, other neurological disorders
	Diabetes and kidney diseases	Acute glomerulonephritis, chronic kidney disease
	Skin and subcutaneous diseases	Fungal skin diseases, pruritus, decubitus ulcer, and other skin and subcutaneous diseases
	Sense organ diseases	Age-related and other hearing loss, other sense organ diseases, blindness, and vision loss
	Musculoskeletal disorders	Low back pain
**Injuries**	Unintentional injuries	Falls, drowning

Data source: GBD 2019

Significant regional and sex disparities in ARD burdens existed in China. Generally, the eastern coast provinces had the lowest crude burden rates, while north-eastern and several western provinces bore the highest ones (Figure 1). After standardizing the age structure, we noted tiered gaps in the age-standardised ARD burden among the eastern, central, and western provinces. Shanghai, Beijing, Zhejiang, and Jiangsu had the lowest burdens, while the burden of Tibet, Qinghai, Guizhou, and Xinjiang were the highest. There were salient disparities between the sexes, with age-standardised ARD burden rates being 61.2% higher among males than females on average. This disparity was most apparent in neoplasms, where the burden rate was 1·5 times higher among males. We observed an inversion of this trend only among neurological disorders, where the burden rate was 10.9% higher among females.

In 2016, China's crude ARD burden-adjusted age was 50·38 years (95% UI: 49.91. 50.91), following the same regional disparity pattern as measured by DALYs (Figure 2). Beijing, Guangdong, Shanghai, Zhejiang and Fujian, all located in eastern coast of China, were youngest with a crude ARD burden-adjusted age below 40. In comparison, Sichuan, Heilongjiang, Chongqing, Jilin and Liaoning were oldest, with the crude age close to or above 60. However, after standardising the age structure across provinces, there were notable changes in the ARD burden-adjusted ages among several western provinces, especially for Tibet, Qinghai, Guizhou and Xinjiang. These four provinces became the oldest provinces upon age structure standardisation, at 72.70 (95% UI:60.53, 85.55), 66.82 (95% UI:59.36, 74.03), 60.53 (95% UI: 53.76, 63.56) and 60.06 (95% UI: 53.00, 66.88) respectively. Shanghai, Beijing, and Zhejiang still had the youngest age-standardised ARD burden-adjusted ages, all of which were below 40.

Between 1990 and 2016, China's age-standardised ARD burden had decreased despite the increasing life expectancy, with a national average decline of 34·6% compared to the 1990 burden. We found regional disparities in the magnitude of this decline. The largest decline was seen in the eastern provinces, followed by the central and western provinces, with the average ARD burdens declining by 35·2%, 33·9%, and 32·7%, respectively (Figure 3). Breaking this down by province, Fujian experienced the largest decline in the age-standardised ARD burden (42·2%), followed by Zhejiang (42·0%), and Beijing (40·7%). Guangxi, Qinghai, and Guizhou experienced the smallest ARD burden decline in the same period, at 27·8%, 27·6%, 27·5%, respectively. The ARD burden declined more sharply in females (41·4%) than males (29·8%). Between 1990 and 2016, the ARD burden had decreased for CVDs, neoplasms, and CRDs, stayed almost

For peer review only - http://bmjopen.bmj.com/site/about/guidelines.xhtml

unchanged for sense organ diseases and increased for neurological disorders (Figure 4). CRDs experienced the sharpest decline (67.9%), followed by neoplasms (30.5%) and CVDs (26·8%). However, the burden of neurological disorders increased by 5% during the same period.

China's health resources grew between 2010 and 2016. The country's total health expenditures per capita increased from 233·9 USD (exchange rate, 1 USD≈6·37 CNY) in 2010 to 526·2 USD in 2016. The total health professional density (number of total health professionals per 1,000 population) increased by 38·6% from 4·4 in 2010 to 6·1 in 2016. In the same period, the licensed doctor density increased by 27·8%, while the licensed nurse density increased by 66·7% (Appendix for Table S3).[39,40] We report the regression results in Table 2. All coefficients of the health resource variables had the expected sign. The model results showed neither higher health expenditures per capita nor higher health workforce density could significantly lead to lower ARD burden, ceteris paribus. However, we found that the existing urban-rural gap in health workforce density was positively associated with the ARD burden, significant at the 10% level for all the three indicators. A 100% increase in the urban-rural ratio in total health professional density, licensed doctor density, and licensed nurse density led to 2·37% (95% CI: -0·41, 5·15), 2·10% (95% CI: -0·27, 4·46), and 2.02% (95% CI: -0·34, 4·39) increases in the ARD burden respectively, ceteris paribus.

For peer review only - http://bmjopen.bmj.com/site/about/guidelines.xhtml

Table 2. Regression model results: Assessing the impact of health resources on age-standardised age-related diseases burden in mainland China, 2010–2016

Independent Variables	Model 1 (Total health professional density)		Model 2 (Licensed doctor density)		Model 3 (Licensed nurse density)	
	Coefficient (β)	95% CI	Coefficient (β)	95% CI	Coefficient (β)	95% CI
Total health expenditures per capita	-2.29	(-6.46, 1.88)	-2.02	(-6.18, 2.14)	-2.32	(-6.56, 1.91)
Health professional density	-1.34	(-3.48, 0.79)	··	··	··	··
Health professional density: urban-rural ratio	2.37*	(-0.41, 5.15)	··	··	··	··
Licensed doctor density	··	··	-0.93	(-3.30, 1.44)	··	··
Licensed doctor density: urban-rural ratio	··	··	2.10*	(-0.27, 4.46)	··	··
Licensed nurse density	··	··	··	··	-1.33	(-3.61, 0.94)
Licensed nurse density: urban-rural ratio	··	··	··	··	2.02*	(-0.34, 4.39)
GDP per capita	-0.16	(-5.09, 4.77)	-1.00	(-6.34, 4.33)	-0.34	(-5.57, 4.89)
Population living in urban areas (%)	1.82	(-15.60, 19.24)	1.83	(-15.13, 18.80)	3.03	(-14.95, 21.01)
Female (%)	-0.60	(-2.50, 1.30)	-0.87	(-2.66, 0.92)	-0.83	(-2.80, 1.14)
≥ junior middle school education (%)	3.25	(-1.66, 8.16)	2.30	(-2.25, 6.84)	3.03	(-1.72, 7.79)
adj. R^2	0.93	··	0.93	··	0.93	··

Data sources: GBD 2016, Statistical Yearbook of China (2011–2017), and the Health Statistical Yearbook of China (2011–2017)

Notes:

All dependent and independent variables were transformed into natural logarithms for regressions except for the time dummies. The independent variables of interest and covariates were rescaled by divided by 100. The coefficients can be interpreted as follows: every 100% increase in X can lead to a β% increase on Y.

Model 1 used total health professional density (per 1,000 population) as a proxy for human resources for health.

Model 2 used licensed doctor density as a proxy (per 1,000 population) for human resources for health.

Model 3 used licensed nurse density as a proxy (per 1,000 population) for human resources for health.

Significance level: *(p<0·10); **(p<0·05); ***(p<0·01)

For peer review only - http://bmjopen.bmj.com/site/about/guidelines.xhtml

Discussion

Healthy ageing has become a focal discussion topic in today's fast-ageing world, as good health in advanced age can provide continued opportunities for social and personal development.[2] The World Health Organisation officially proposed the concept of healthy ageing in 2015 and published the Global Strategy and Action Plan on Ageing and Health (2016–2020) in 2016. [2,41] The Decade of Healthy Ageing: Plan of Action (2020–2030) was issued in 2020 and asked for a whole-of-government and whole-of-society response to healthy ageing.[42] In response to this international call and the needs of the world's largest ageing population, China has actively worked to address the shifts catalysed by an ageing society and promote healthy ageing.[43–45] We conducted this study to generate high-quality evidence to help China and similar countries be better equipped to face health challenges in ageing societies. To our knowledge, this is the first study that assesses the health burden of ageing and its longitudinal changes in 31 provinces of mainland China from 1990 to 2016. The analysis explored disparities in ARD burdens across regions, sexes, and disease categories and used panel regression models to examine the impact of health resource indicators on the ARD burden.

Our findings underscore several key messages on the health burden of ageing in China. First, NCDs account for over 90% of the country's total ARD burden. CVDs, neoplasms, CRDs, sense organ diseases and neurological disorders were the top five contributors to the ARD burden. Second, there are significant regional disparities in ARD burdens. The results show tiered differences in the ARD burden, with the lowest rates in the eastern coast provinces, followed by the central provinces, and the highest rates in the north-eastern and western provinces. Notably, the crude ARD burden rate in Sichuan was 2·1 times that of Beijing in 2016, equal to a 34·14-year age gap measured by the ARD burden-adjusted age defined in this study. Third, several western provinces will face daunting ARD-induced challenges as their populations begin to age. After age structure standardisation, Tibet, Qinghai, Guizhou, and Xinjiang had the four highest ARD burdens. However, in 2016, less than 10% of each of these provinces' populations were aged 65 and older, compared to a national average of 10.8%.[30] Fourth, males are disproportionally affected by ARDs except for neurological disorders. Fifth, the overall age-standardised ARD burden has been decreasing since 1990, though the largest decline was observed in the eastern provinces, followed by the central and western provinces. By disease category, CRDs experienced the largest decline in age-standardised burden, followed by neoplasms and CVDs. The burden rate of sense organ diseases had stayed almost unchanged since 1990. The burden of neurological disorders has increased since 1990, albeit by a small magnitude. Lastly, our results suggest that greater investment in health to reduce the urban-rural gap in human resources for health could help lower China's ARD burden.

Our study findings are consistent with previous studies that assessed the ARD burden globally and in China.[21,22] There is overall support for the idea that NCDs are the chief contributor to ARDs. As has been the case across the globe, the ARD burden has continuously decreased since 1990, declining most sharply in developed countries and regions. Disparities by sex are also similar; males are more negatively affected by the ARD burden and have experienced a lower historical decline. The magnitude of the burden measured in this study is slightly different than those in other studies due to differences across GBD datasets and the populations and diseases included. Our findings are novel in that we were able to measure the ARD burden and disparities across provinces in China, and examine the impact of health resource indicators on the burden. Nevertheless, the regional disparities of age-standardised ARD burdens are in line with their health development status and similar to the disparities in estimated healthy life expectancy.[46–33] The rank of NCD burdens among the ARDs is consistent with that of the disease burden among older people in China.[34] Interestingly, we found that increasing health expenditures or workforce density does not significantly decrease the ARD burden; rather, the key is to reduce the urban-rural gap in the health workforce density. Previous evidence has indicated that increasing health workforce density

For peer review only - http://bmjopen.bmj.com/site/about/guidelines.xhtml

can improve health outcomes.[23,24] However, these studies focus on measuring health outcomes such as maternal mortality, infant, and under-five mortality as opposed to ageing-related outcomes. In addition, these studies did not control for geographic resource differences between rural and urban areas and were conducted in countries where resources were scarce. Thus, the context was quite different from that of the present study.

The Chinese State Council published *The Opinions on Strengthening Aged Care Work in the New Era* in November 2021, outlining eight important action domains for addressing ageing. This document serves as a comprehensive, strategic plan to achieve healthy and active ageing in China.[45] The findings from the current study provide timely input into policy development for the governments of China to promote healthy ageing, by reducing the health burden of ageing. First, to reiterate the findings from previous studies, chronological age alone does not provide a strong enough basis for appropriate ageing resource planning or policymaking.[21,22] In China, provinces or municipalities with older populations, such as Shanghai, Beijing, and Zhejiang, have much lower ARD burdens than some younger provinces such as Tibet and Xinjiang. This phenomenon calls for more careful consideration of underlying health burdens and potential threats to resource planning and policy design to address future ageing. Second, it is crucial to continue and strengthen NCD prevention and control efforts, especially for CVDs, neoplasms, CRDs, sense organ diseases and neurological disorders. To prevent the continued increase in the ARD burden from neurological disorders, it is imperative to allocate additional health resources for the prevention, treatment, and management of conditions such as Alzheimer's disease, dementia, Parkinson's disease, and idiopathic epilepsy. Sense organ diseases cannot be ignored, especially those leading to blindness and vision loss that can significantly damage the quality of life for the elderly. Third, the central government should continue to provide all-around support to the regions that face the greatest threat of high ARD burdens due to future ageing, especially the western provinces of Tibet, Qinghai, Guizhou, and Xinjiang to reduce regional disparities. Notably, that support should entail the establishment of a strong health workforce that can serve the local people. Fourth, aligned with the Rural Revitalization Strategic Plan issued by the State Council (2018–2022) in 2018,[47] both central and local governments should work collaboratively to strengthen the health system in rural areas, particularly increasing the health workforce density to reduce the urban-rural gap.

Our team have carefully compared the difference between estimates from GBD 2016 and 2019 study to ensure the robustness of the findings from the current study. Methodologically, the GBD 2019 study generally takes a similar approach as used in the GBD 2017 study, which has enriched data input sources, modelling parameters, diseases categories and upgraded calibration methods to get more accurate results as compared to the GBD 2016 study.[35,36] Our comparison results show that the crude ARD burden rate in 2016 based on estimates from GBD 2019 is slightly higher than estimates from GBD 2016, though the difference is within 8% and as low as 2% for neoplasms. Regarding the diseases included, we did not find significant changes at level three that would impact the results of the ARD burden. The findings are consistent with the comparison conducted by a previous study on the estimates generated by the GBD 2016 and 2017 study for subnational level of China.[33] Therefore, subnational estimates of GBD 2016 can well represent the regional disparities of ARD burden in China.

Our study has several limitations. First, the subnational analysis was based on the GBD 2016 study estimates, which was not updated to 2019, as we do not have access to the GBD 2019 estimates at subnational level of China. However, this was the best data source we could access and as stated above, the results from the GBD 2016 study are robust to show regional disparities after careful comparison. Second, our provincial analysis of the ARD burden focused on NCDs due to data availability. However, as NCDs accounted for over 90% of the ARD burden in China, these results can accurately represent existing

For peer review only - http://bmjopen.bmj.com/site/about/guidelines.xhtml

regional disparities. Third, the study findings tend to underestimate the ARD burden, as GBD study estimates fail to model the interactions between diseases, ignoring the burden caused by multimorbidity. Fourth, we had to rely on interpolation to obtain annual estimates of burden rates from 2010 to 2016 for panel data analysis, though the GBD research team used the same methods in their analysis.[38] In addition, the indicators to measure health resources limit to total health expenditures and health workforce density, largely due to data availability. Other important indicators, such as efficiency of health funds utilization, quality of human resources for health, could be explored in future research when data become available.

Conclusion

In conclusion, our study provides valuable and original insights into the health burden of ageing in China and the existing regional disparities. Our results consistently show that an older demographic structure does not necessarily mean a heavier health burden, and therefore chronological age alone does not provide a strong enough basis for appropriate ageing resource planning or policymaking. Continuing to invest in population health through reducing the urban-rural gap in health workforce density would be helpful to decrease the health burden of ageing in China. The governments of China, or other countries with similar demographic and disease profiles and development contexts, need to take urgent action to decrease the health burden of ageing to create healthy ageing societies.

Contributors

SC designed the study under the supervision of KH, BL, and HB. SC extracted the data, conducted the statistical analysis in Stata, developed the tables and figures, and drafted the manuscript. SC and YS verified the data in the study. ST obtained the GBD data. All authors contributed to commenting, editing, and approval of the final manuscript. All accept responsibility to submit for publication.

Declaration of interests

All co-authors declare no conflicts of interest for this study.

Acknowledgement

We thank the Institute for Health Metrics and Evaluation (IHME) for making this study possible by sharing the GBD 2016 study estimates from China at the subnational level. We also appreciate the technical instructions from Dr. Cheng Wan (UNSW) and Mr. Lei Guo (Duke Global Health Institute) on drawing the maps.

Funding statement

The study received funding support from the Bill & Melinda Gates Foundation (OPP1148464), the UNSW, and the ARC Centre of Excellence in Population Ageing Research (CEPAR), SHARP Fund UNSW (SHARP001). The funders had no role in study design, data collection, data analysis, data interpretation, or manuscript writing.

Data availability statement

China's subnational-level data from GBD 2016 were obtained from the IHME under a private agreement. Researchers can apply for data access at https://www.healthdata.org/about/contact-us if interested in using the data. Other inputs data were obtained from open resources, including China Statistical Yearbook (http://www.stats.gov.cn/tjsj/ndsj/) and China Health Statistical Yearbook (http://www.nhc.gov.cn/wjw/tjnj/list.shtml). Researchers can access and download the data from these websites.

For peer review only - http://bmjopen.bmj.com/site/about/guidelines.xhtml

Ethics statement

Ethics approval

This study received ethical approval from the University of South Wales (UNSW) Ethics Committee (HC210794).

Patient consent for publication

Noa applicable. The study does not collect data at individual level.

For peer review only

For peer review only - http://bmjopen.bmj.com/site/about/guidelines.xhtml

References

1 UN Department of Economic and Social Affairs, Population Division. World population ageing, 2019 highlights. 2020.

2 Beard JR, Officer A, de Carvalho IA, *et al.* The World report on ageing and health: a policy framework for healthy ageing. *The Lancet* 2016; **387**: 2145–54.

3 Manton KG, Gu X, Lamb VL. Change in chronic disability from 1982 to 2004/2005 as measured by long-term changes in function and health in the U.S. elderly population. *Proc Natl Acad Sci* 2006; **103**: 18374–9.

4 Chatterji S, Byles J, Cutler D, Seeman T, Verdes E. Health, functioning, and disability in older adults—present status and future implications. *The Lancet* 2015; **385**: 563–75.

5 Seeman TE, Merkin SS, Crimmins EM, Karlamangla AS. Disability Trends Among Older Americans: National Health and Nutrition Examination Surveys, 1988–1994 and 1999–2004. *Am J Public Health* 2010; **100**: 100–7.

6 Crimmins EM, Beltran-Sanchez H. Mortality and Morbidity Trends: Is There Compression of Morbidity? *J Gerontol B Psychol Sci Soc Sci* 2011; **66B**: 75–86.

7 Kyu HH, Abate D, Abate KH, *et al.* Global, regional, and national disability-adjusted life-years (DALYs) for 359 diseases and injuries and healthy life expectancy (HALE) for 195 countries and territories, 1990–2017: a systematic analysis for the Global Burden of Disease Study 2017. *The Lancet* 2018; **392**: 1859–922.

8 Lafortune G, Balestat G. Trends in Severe Disability Among Elderly People: Assessing the Evidence in 12 OECD Countries and the Future Implications. Paris, 2007 DOI:10.1787/217072070078.

9 Jagger C, Gillies C, Moscone F, *et al.* Inequalities in healthy life years in the 25 countries of the European Union in 2005: a cross-national meta-regression analysis. 2008; **372**: 8.

10 UN Department of Economic and Social Affairs Population Division. World Population Ageing: 1950-2050. New York, 2017 http://globalag.igc.org/ruralaging/world/ageingo.htm (accessed Aug 12, 2021).

11 Fried LP, Tangen CM, Walston J, *et al.* Frailty in Older Adults: Evidence for a Phenotype. *J Gerontol A Biol Sci Med Sci* 2001; **56**: M146–57.

12 Searle SD, Mitnitski A, Gahbauer EA, Gill TM, Rockwood K. A standard procedure for creating a frailty index. *BMC Geriatr* 2008; **8**: 24.

13 Edemekong PF, Bomgaars DL, Sukumaran S, Levy SB. Activities of Daily Living. In: StatPearls. Treasure Island (FL): StatPearls Publishing, 2021. http://www.ncbi.nlm.nih.gov/books/NBK470404/ (accessed Aug 11, 2021).

14 Kempen GIJM, Suurmeijer TPBM. The Development of a Hierarchical Polychotomous ADL-IADL Scale for Noninstitutionalized Elders. *The Gerontologist* 1990; **30**: 497–502.

For peer review only - http://bmjopen.bmj.com/site/about/guidelines.xhtml

15 Mahoney F, Barthel D. FUNCTIONAL EVALUATION: THE BARTHEL INDEX - PubMed. *Md State Med J* 1965; **14**: 61–5.

16 Skirbekk V, Loichinger E, Weber D. Variation in cognitive functioning as a refined approach to comparing aging across countries. *Proc Natl Acad Sci* 2012; **109**: 770–4.

17 Beard JR, Jotheeswaran AT, Cesari M, Araujo de Carvalho I. The structure and predictive value of intrinsic capacity in a longitudinal study of ageing. *BMJ Open* 2019; **9**: e026119.

18 Beard J, Si Y, Chenoweth L, Hanewald K. Intrinsic Capacity: Validation of a New WHO Concept for Healthy Ageing in a Longitudinal Chinese Study. *J Gerontol A Biol Sci Med Sci* 2021; published online Aug 3. DOI:10.1093/gerona/glab226.

19 Brooks R. EuroQol: the current state of play. *Health Policy Amst Neth* 1996; **37**. DOI:10.1016/0168-8510(96)00822-6.

20 EuroQol Group. EuroQol - a new facility for the measurement of health-related quality of life. *Health Policy* 1990; **16**: 199–208.

21 Chang AY, Skirbekk VF, Tyrovolas S, Kassebaum NJ, Dieleman JL. Measuring population ageing: an analysis of the Global Burden of Disease Study 2017. *Lancet Public Health* 2019; **4**: e159–67.

22 Hu D, Yan W, Zhu J, Zhu Y, Chen J. Age-Related Disease Burden in China, 1997–2017: Findings From the Global Burden of Disease Study. *Front Public Health* 2021; **0**. DOI:10.3389/fpubh.2021.638704.

23 Anand S, Bärnighausen T. Human resources and health outcomes: cross-country econometric study. *The Lancet* 2004; **364**: 1603–9.

24 Speybroeck N, KINFU Y, Dal Poz M, Evans D. Reassessing the Relationship Between Human Resources for Health, Intervention Coverage and Health Outcomes. *Reassessing Relatsh Hum Resour Health Interv Cover Health Outcomes* 2006; : 1–14.

25 Nguyen MP, Mirzoev T, Le TM. Contribution of health workforce to health outcomes: empirical evidence from Vietnam. *Hum Resour Health* 2016; **14**: 68.

26 Panopoulou E, Pantelidis T. Convergence in per capita health expenditures and health outcomes in the OECD countries. *Appl Econ* 2012; **44**: 3909–20.

27 Bokhari FAS, Gai Y, Gottret P. Government health expenditures and health outcomes. *Health Econ* 2007; **16**: 257–73.

28 Nixon J, Ulmann P. The relationship between health care expenditure and health outcomes. Evidence and caveats for a causal link. *Eur J Health Econ HEPAC Health Econ Prev Care* 2006; **7**: 7–18.

29 Nketiah-Amponsah E. The Impact of Health Expenditures on Health Outcomes in Sub-Saharan Africa. *J Dev Soc* 2019; **35**: 134–52.

30 National Bureau of Statistics, China. National data. 2020. https://data.stats.gov.cn/easyquery.htm?cn=E0103 (accessed Aug 12, 2021).

For peer review only - http://bmjopen.bmj.com/site/about/guidelines.xhtml

For peer review only

31 Zhou M, Wang H, Zeng X, *et al.* Mortality, morbidity, and risk factors in China and its provinces, 1990–2017: a systematic analysis for the Global Burden of Disease Study 2017. *The Lancet* 2019; **394**: 1145–58.

32 Li H, Hanewald K, Wu S. Healthy life expectancy in China: Modelling and implications for public and private insurance. *Ann Actuar Sci* 2021; **15**: 40–56.

33 Chen S, Guo L, Wang Z, *et al.* Current situation and progress toward the 2030 health-related Sustainable Development Goals in China: A systematic analysis. *PLOS Med* 2019; **16**: e1002975.

34 Global Burden of Disease Collaborative Network. Global Burden of Disease Study 2019 (GBD 2019) Results. 2020. http://ghdx.healthdata.org/gbd-results-tool (accessed Aug 10, 2021).

35 Vos T, Lim SS, Abbafati C, *et al.* Global burden of 369 diseases and injuries in 204 countries and territories, 1990–2019: a systematic analysis for the Global Burden of Disease Study 2019. *The Lancet* 2020; **396**: 1204–22.

36 Vos T, Abajobir AA, Abate KH, *et al.* Global, regional, and national incidence, prevalence, and years lived with disability for 328 diseases and injuries for 195 countries, 1990–2016: a systematic analysis for the Global Burden of Disease Study 2016. *The Lancet* 2017; **390**: 1211–59.

37 IHME. Frequently Asked Questions. Inst. Health Metr. Eval. 2014; published online May 9. http://www.healthdata.org/gbd/faq (accessed Nov 15, 2021).

38 Vos T, Lim SS, Abbafati C, *et al.* Global burden of 369 diseases and injuries in 204 countries and territories, 1990–2019: a systematic analysis for the Global Burden of Disease Study 2019. *The Lancet* 2020; **396**: 1204–22.

39 National Health and Family Planning Commission. China Health and Family Planning Statistical Yearbook 2017. Beijing: Peking Union Medical College Press, 2017.

40 Ministry of Health, China. China Health Statistical Yearbook 2011. Beijing: Peking Union Medical College Press, 2011.

41 Global strategy and action plan on ageing and health. https://www.who.int/publications-detail-redirect/9789241513500 (accessed Dec 3, 2021).

42 World Health Organization. UN Decade of Healthy Ageing. https://www.who.int/initiatives/decade-of-healthy-ageing (accessed Dec 3, 2021).

43 Yang W, Chang S, Zhang W, *et al.* An Initial Analysis of the Effects of a Long-Term Care Insurance on Equity and Efficiency: A Case Study of Qingdao City in China. *Res Aging* 2021; **43**: 156–65.

44 Feng Z, Glinskaya E, Chen H, *et al.* Long-term care system for older adults in China: policy landscape, challenges, and future prospects. *The Lancet* 2020; **396**: 1362–72.

45 The State Council, China. The Opinions on Strengthening Aged Care Work in the New Era. 2021; published online Nov 24. http://www.gov.cn/zhengce/2021-11/24/content_5653181.htm (accessed Dec 3, 2021).

For peer review only

For peer review only - http://bmjopen.bmj.com/site/about/guidelines.xhtml

46 Li H, Hanewald K, Wu S. Estimating Healthy Life Expectancy: A Province-by-Province Study for China. Rochester, NY: Social Science Research Network, 2017 DOI:10.2139/ssrn.3027825.

47 The State Council, China. Rural Revitalization Strategic Plan (2018-2022). 2018; published online Sept 26. http://www.moa.gov.cn/ztzl/xczx/xczxzlgh/201811/t20181129_6163953.htm (accessed Dec 3, 2021).

For peer review only

For peer review only - http://bmjopen.bmj.com/site/about/guidelines.xhtml

Figure legend and footnote

Figure 1. The crude and age-standardised age-related diseases burden in 31 provinces of mainland China, 2016

Data source: GBD 2016
Note: The age-related diseases burden was standardized using the IHME standard population age structure (appendix)

Figure 2. The crude and age-standardised ARD burden-adjusted ages of 31 provinces in mainland China, 2016

Data source: GBD 2016
Note: The provinces were grouped into eastern, central and western provinces from top down.
The age-related diseases burden was standardized using the IHME standard population age structure (appendix)

Figure 3. Change of the age-standardised age-related diseases burden rates by administrative region in mainland China, 1990-2016

Data source: GBD 2016
Note: The age-related diseases burden was standardized using the IHME standard population age structure (appendix)

Figure 4. Change of the age-standardised age-related diseases burden rates of the five leading ARD contributors in China, 1990-2016

Data source: GBD 2016
Note: The age-related diseases burden was standardized using the IHME standard population age structure (appendix)

For peer review only - http://bmjopen.bmj.com/site/about/guidelines.xhtml

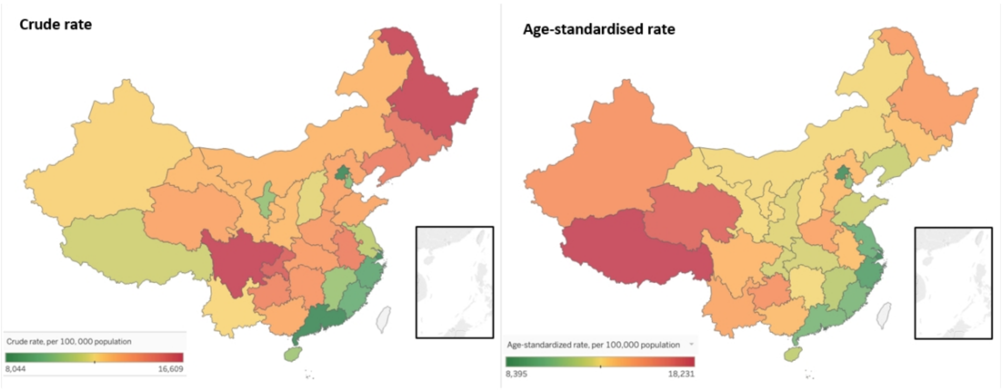

The crude and age-standardised age-related diseases burden in 31 provinces of mainland China, 2016

832x322mm (38 x 38 DPI)

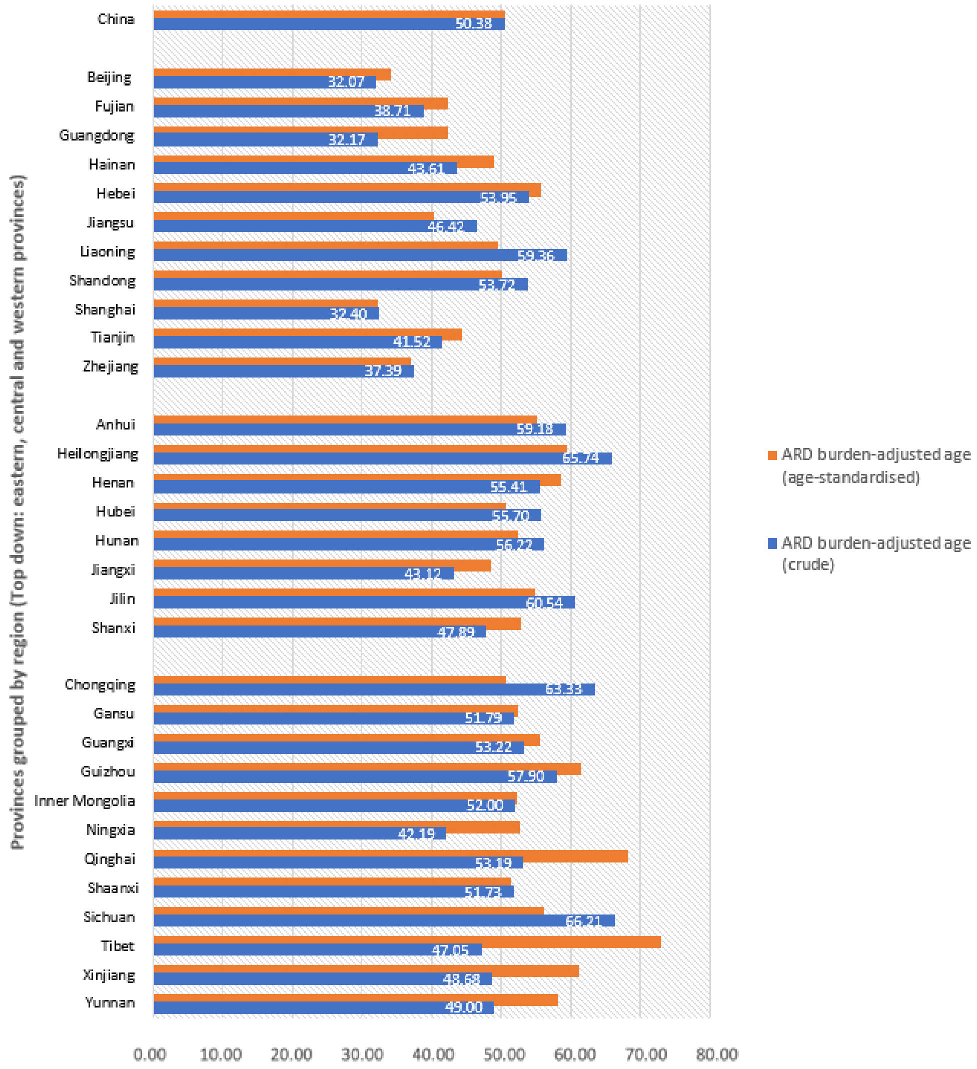

The crude and age-standardised ARD burden-adjusted ages of 31 provinces in mainland China, 2016

437x482mm (38 x 38 DPI)

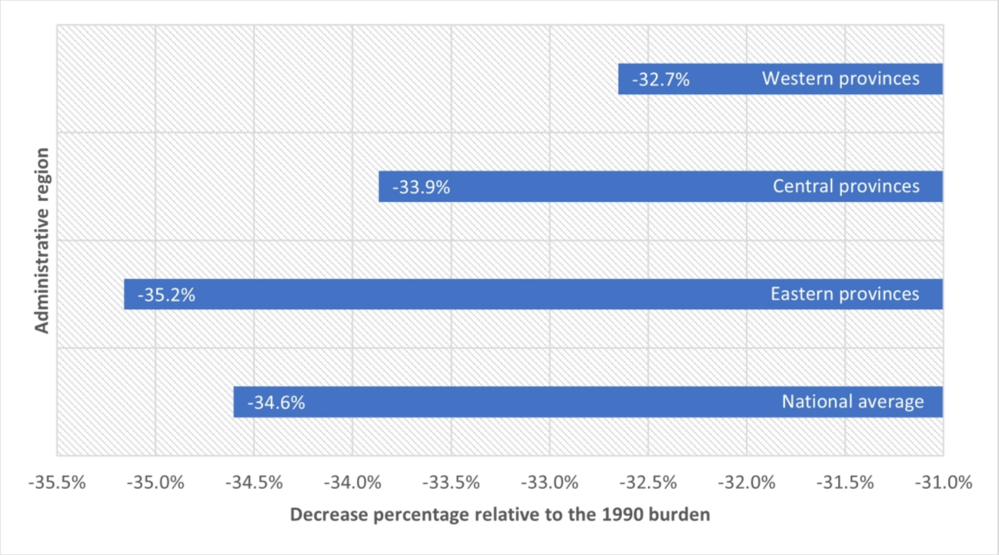

Change of the age-standardised age-related diseases burden rates by administrative region in mainland China, 1990-2016

389x216mm (130 x 130 DPI)

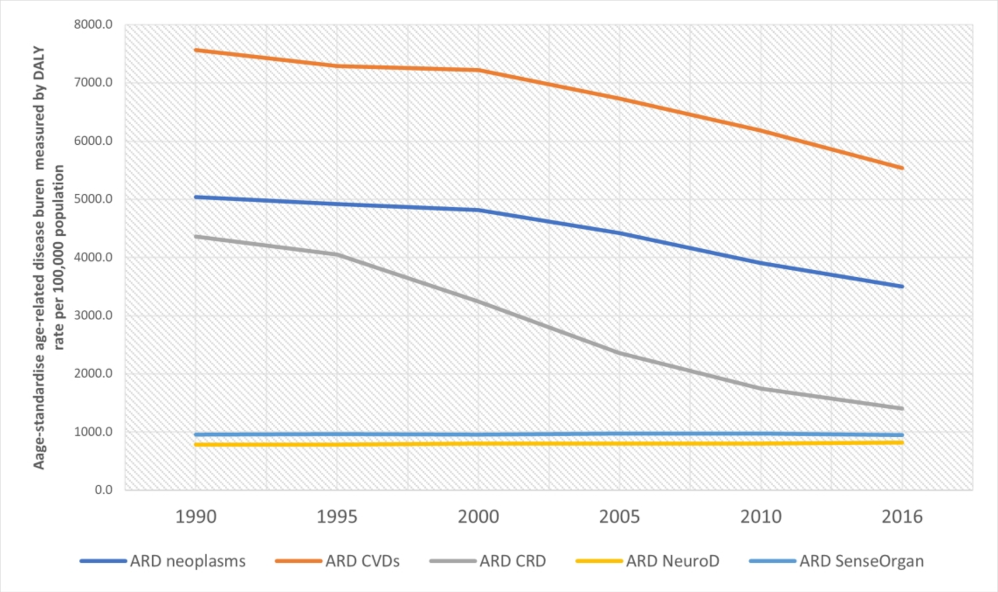

Change of the age-standardised age-related diseases burden rates of the five leading ARD contributors in China, 1990-2016

391x232mm (130 x 130 DPI)

Appendix

Table S1. Administrative division of provinces in mainland China

**Administrative division (No.)**	**Province**
Eastern provinces (11)	Beijing, Fujian, Guangdong, Hainan, Hebei, Jiangsu, Liaoning, Shandong Shanghai, Tianjin, Zhejiang
Central provinces (8)	Anhui, Heilongjiang, Henan, Hubei, Hunan, Jiangxi, Jilin, Shanxi
Western provinces (12)	Inner Mongolia, Guangxi, Chongqing, Gansu, Guizhou, Ningxia, Qinghai, Shaanxi, Sichuan, Tibet, Xinjiang, Yunnan

Table S2. IHME standard age weight

Age group	Population percentage
<5	7.2%
5–9	8.7%
10–14	8.4%
15–19	8.1%
20–24	7.8%
25–29	7.6%
30–34	7.2%
35–39	6.9%
40–44	6.4%
45–49	5.8%
50–54	5.3%
55–59	4.7%
60–64	4.1%
65–69	3.4%
70 +	8.6%

ARD burden-adjusted age calculation

First, we identified the ARD burden-adjusted age of China by comparing the national average burden rate with its burden rates by age group. We selected the two five-year age groups with closest burden rate as the national average, and calculated the national average age by assuming linear increase of the ARD burden within each five-year age group. For example, the age-related disease burden of China was 12637.2 DALYs per 100,000 population in 2016, closest to burden rates for age group of 45–49 (9316.3) and 50–54 (14163.7). Hence, the national age was calculated as:
45+12637.2/((9316.3+14163.7)/10)=50.38.

For peer review only - http://bmjopen.bmj.com/site/about/guidelines.xhtml

For peer review only

Second, we calculated the ARD burden-adjusted age of each province by dividing the provincial age-related disease burden rate by the unit share of the national average burden rate per age. The unit share of the national average burden rate per age was calculated by dividing the national average burden rate by the national equivalent age. For example, the age-related disease burden of Shanghai was 8,127·4 DALYs per 100,000 population. Hence, Shanghai's ARD burden-adjusted age was calculated as: 8,127·4/(12637·2/50.38)=32·40.

Regression model

$$Y_{it} = \alpha + \beta_0 healthexp_{it} + \beta_1 healthwf_{it} + \beta_2 X_{it} + \beta_3 D_t + \eta_i + \varepsilon_{it},$$

where Y_{it} is the age-standardised age-related disease burden of Province i in Year t, β_0 is the coefficient of interest, $healthexp_{it}$ is the total health expenditures per capita, and $healthwf_{it}$ is the total health professional density, licensed doctor density, or licensed nurse density (per 1,000 population) of the Province i in Year t (three separate models). X_{it} is the set of covariates controlled in the model, including GDP per capita, education, the proportion of females, the proportion of people living in urban areas, and the urban-rural health workforce density ratio. D_t represents the time dummies, η_i the province fixed effects, and ε_{it} is the error term. All variables are in log form except for the time dummies.

For peer review only - http://bmjopen.bmj.com/site/about/guidelines.xhtml

Table S3. Selected statistics of the variables in the regression model, 2010 and 2016

Variables	Health expenditures per capita (CNY)*		Total health professionals density (per 1,000 population)		Urban-rural ratio in total health professionals density		GDP per capita (CNY)*		Living in urban areas (%)		Female (%)		Received at least middle school education (%)	
Province	2010	2016	2010	2016	2010	2016	2010	2016	2010	2016	2010	2016	2010	2016
Anhui	1210·5	2652·17	3·1	4·7	2·4	2·3	20888·0	39561·0	44.8%	52.0%	49·2%	48·7%	62·8%	65·5%
Beijing	4147·2	9429·73	13·6	10·8	1·9	··	73856·0	118198·0	86.2%	86.5%	48·4%	48·6%	87·6%	88·6%
Chongqing	1501·0	3492·19	3·4	5·9	1·4	2·0	27596·0	58502·0	55.0%	62.6%	49·4%	49·2%	61·0%	64·1%
Fujian	1280·1	3226·83	4·1	5·7	2·8	2·5	40025·0	74707·0	58.1%	63.6%	48·6%	49·1%	63·0%	61·7%
Gansu	1153·9	2889·18	3·7	5·2	2·0	2·2	16113·0	27643·0	37.2%	44.7%	48·9%	49·3%	56·5%	57·9%
Guangdong	1445·9	3812·46	5·3	6·0	3·2	3·2	44736·0	74016·0	66.5%	69.2%	47·8%	46·9%	73·2%	74·3%
Guangxi	1116·9	2557·03	3·6	6·0	2·2	2·2	20219·0	38027·0	41.8%	48.1%	48·0%	48·0%	62·5%	67·4%
Guizhou	946·6	2472·37	2·5	5·8	4·2	3·9	13119·0	33246·0	35.0%	44.2%	48·3%	48·4%	48·8%	54·6%
Hainan	1193·0	3306·78	4·4	6·3	2·3	3·3	23831·0	44347·0	50.5%	56.8%	47·4%	47·3%	72·1%	73·0%
Hebei	1253·8	2710·58	4·0	5·3	3·2	2·7	28668·0	43062·0	45.6%	53.3%	49·3%	48·9%	69·9%	69·7%
Heilongjiang	1580·2	3133·43	5·0	5·8	2·3	2·3	27076·0	40432·0	56.5%	59.2%	49·2%	49·5%	73·0%	73·3%
Henan	1134·0	2594·0	3·5	5·7	3·0	3·2	24446·0	42575·0	40.6%	48.5%	49·5%	49·0%	68·2%	69·8%
Hubei	1191·1	3270·56	4·2	6·5	2·1	2·2	27906·0	55665·0	51.8%	58.1%	48·6%	48·7%	69·8%	70·5%
Hunan	1042·1	2820·97	3·8	5·8	2·9	2·9	24719·0	46382·0	45.1%	52.8%	48·6%	48·9%	67·1%	71·2%

For peer review only

For peer review only - http://bmjopen.bmj.com/site/about/guidelines.xhtml

Inner Mongolia	1767·5	3599·7	5·1	6·8	2·7	2·6	47347·0	72064·0	56.6%	61.2%	48·1%	49·5%	69·9%	73·3%
Jiangsu	1566·0	4200·21	4·4	6·5	2·0	2·1	52840·0	96887·0	61.9%	67.7%	49·6%	49·6%	70·9%	71·5%
Jiangxi	992·0	2374·79	5·1	6·1	1·8	2·1	21253·0	40400·0	45.7%	53.1%	48·2%	48·0%	65·6%	64·1%
Jilin	1653·9	3501·19	5·5	6·3	2·5	2·9	31599·0	53868·0	53.4%	56.0%	49·3%	49·2%	69·8%	73·6%
Liaoning	1765·9	3390·9	4·7	6·6	3·0	2·6	42355·0	50791·0	64.0%	67.4%	49·4%	49·5%	75·5%	78·3%
Ningxia	1190·1	3730·50	4·5	6·2	4·4	5·4	26860·0	47194·0	49.8%	56.3%	48·8%	48·4%	61·3%	66·5%
Qinghai	1472·0	4043·05	4·7	7·6	2·0	2·0	24115·0	43531·0	46.3%	51.6%	48·2%	48·6%	49·8%	51·8%
Shaanxi	2040·7	3535·66	4·7	6·5	1·7	2·2	27133·0	51015·0	47.3%	55.3%	48·3%	49·5%	69·8%	71·5%
Shandong	1403·1	3372·70	9·7	7·4	1·3	1·6	41106·0	68733·0	50.9%	59.0%	49·4%	49·0%	67·5%	69·0%
Shanghai	2828·1	7596·0	5·6	6·1	2·6	3·2	76074·0	116562·0	89.3%	87.9%	48·5%	48·6%	83·6%	83·5%
Shanxi	1297·5	2650·33	3·4	4·8	2·7	3·0	26283·0	35532·0	49.7%	56.2%	48·6%	48·5%	74·1%	77·5%
Sichuan	1019·1	3238·64	3·6	6·0	2·0	2·0	21182·0	40003·0	41.8%	49.2%	49·2%	50·1%	57·2%	58·9%
Tianjin	2737·3	5294·21	7·1	6·1	1·4	1·1	72994·0	115053·0	80.5%	82.9%	46·6%	46·6%	80·5%	82·0%
Tibet	1472·0	3780·9	3·4	4·5	4·9	4·1	17027·0	35184·0	22.8%	29.6%	48·6%	49·5%	26·4%	29·6%
Xinjiang	1676·8	4012·9	5·7	7·1	3·2	2·4	25034·0	40564·0	43.5%	48.3%	48·7%	48·9%	66·1%	65·5%
Yunnan	1107·2	2754·1	3·2	5·2	3·2	3·3	15752·0	31093·0	36.8%	45.0%	48·1%	49·5%	48·3%	52·5%
Zhejiang	2099·0	4603·84	6·1	7·7	1·8	1·8	51711·0	84916·0	62.3%	67.0%	48·6%	47·9%	64·7%	66·0%

*CNY=Chinese Yuan, exchange rate: 1USD≈6.37CNY (Dec 3, 2020)

Data source: National Statistical Yearbook of China (2011, 2017) and National Health Statistical Yearbook of China (2011,2017)

For peer review only - http://bmjopen.bmj.com/site/about/guidelines.xhtml

GATHER
Guidelines for Accurate and Transparent Health Estimates Reporting

Checklist of information that should be included in new reports of global health estimates

Item #	Checklist item	Reported on page #
**Objectives and funding**		
**1**	Define the indicator(s), populations (including age, sex, and geographic entities), and time period(s) for which estimates were made.	4
**2**	List the funding sources for the work.	6
**Data Inputs**		
For all data inputs from multiple sources that are synthesized as part of the study:		
**3**	Describe how the data were identified and how the data were accessed.	4 & 9
**4**	Specify the inclusion and exclusion criteria. Identify all ad-hoc exclusions.	4
**5**	Provide information on all included data sources and their main characteristics. For each data source used, report reference information or contact name/institution, population represented, data collection method, year(s) of data collection, sex and age range, diagnostic criteria or measurement method, and sample size, as relevant.	4 & 5
**6**	Identify and describe any categories of input data that have potentially important biases (e.g., based on characteristics listed in item 5).	4 & 5 & 9
For data inputs that contribute to the analysis but were not synthesized as part of the study:		
**7**	Describe and give sources for any other data inputs.	4 & 5 & 9
For all data inputs:		
**8**	Provide all data inputs in a file format from which data can be efficiently extracted (e.g., a spreadsheet rather than a PDF), including all relevant meta-data listed in item 5. For any data inputs that cannot be shared because of ethical or legal reasons, such as third-party ownership, provide a contact name or the name of the institution that retains the right to the data.	9 and appendix
**Data analysis**		
**9**	Provide a conceptual overview of the data analysis method. A diagram may be helpful.	4 & 5
**10**	Provide a detailed description of all steps of the analysis, including mathematical formulae. This description should cover, as relevant, data cleaning, data pre-processing, data adjustments and weighting of data sources, and mathematical or statistical model(s).	4 & 5
**11**	Describe how candidate models were evaluated and how the final model(s) were selected.	4 & 5
**12**	Provide the results of an evaluation of model performance, if done, as well as the results of any relevant sensitivity analysis.	7 & 15 & 22 & 23
**13**	Describe methods for calculating uncertainty of the estimates. State which sources of uncertainty were, and were not, accounted for in the uncertainty analysis.	5
**14**	State how analytic or statistical source code used to generate estimates can be accessed.	5 & 6
**Results and Discussion**		
**15**	Provide published estimates in a file format from which data can be efficiently extracted.	12-15
**16**	Report a quantitative measure of the uncertainty of the estimates (e.g. uncertainty intervals).	6,7,15
**17**	Interpret results in light of existing evidence. If updating a previous set of estimates, describe the reasons for changes in estimates.	6-9
**18**	Discuss limitations of the estimates. Include a discussion of any modelling assumptions or data limitations that affect interpretation of the estimates.	8-9

This checklist should be used in conjunction with the GATHER statement and Explanation and Elaboration document, found on gather-statement.org

For peer review only - http://bmjopen.bmj.com/site/about/guidelines.xhtml

BMJ Open

Disease Burden of Ageing, Sex and Regional Disparities, and Health Resources Allocation: A Longitudinal Analysis of 31 Provinces in Mainland China

Journal:	*BMJ Open*
Manuscript ID	bmjopen-2022-064641.R1
Article Type:	Original research
Date Submitted by the Author:	25-Aug-2022
Complete List of Authors:	Chen, Shu; UNSW, School of Risk and Actuarial Studies; UNSW, ARC Centre of Excellence in Population Ageing Research (CEPAR)<br>Si, Yafei; University of New South Wales, School of Risk and Actuarial Studies; University of New South Wales, ARC Centre of Excellence in Population Ageing Research (CEPAR)<br>Hanewald, Katja; University of New South Wales, School of Risk and Actuarial Studies; University of New South Wales, ARC Centre of Excellence in Population Ageing Research (CEPAR)<br>Li, Bingqin; University of New South Wales, Social Policy Research Center<br>Bateman, Hazel; University of New South Wales, School of Risk and Actuarial Studies; University of New South Wales, ARC Centre of Excellence in Population Ageing Research (CEPAR)<br>Dai, Xiaochen; Institute of Health Metrics and Evaluation<br>wu, chenkai; Duke Kunshan University, Global Health Research Center<br>Tang, Shenglan; Duke Kunshan University, Global Health Research Center; Duke University, Duke Global Health Institute
**Primary Subject Heading**:	Health policy
Secondary Subject Heading:	Public health, Epidemiology, Geriatric medicine
Keywords:	Health policy < HEALTH SERVICES ADMINISTRATION & MANAGEMENT, EPIDEMIOLOGY, Health economics < HEALTH SERVICES ADMINISTRATION & MANAGEMENT, PUBLIC HEALTH

SCHOLARONE™
Manuscripts

I, the Submitting Author has the right to grant and does grant on behalf of all authors of the Work (as defined in the below author licence), an exclusive licence and/or a non-exclusive licence for contributions from authors who are: i) UK Crown employees; ii) where BMJ has agreed a CC-BY licence shall apply, and/or iii) in accordance with the terms applicable for US Federal Government officers or employees acting as part of their official duties; on a worldwide, perpetual, irrevocable, royalty-free basis to BMJ Publishing Group Ltd ("BMJ") its licensees and where the relevant Journal is co-owned by BMJ to the co-owners of the Journal, to publish the Work in this journal and any other BMJ products and to exploit all rights, as set out in our licence.

The Submitting Author accepts and understands that any supply made under these terms is made by BMJ to the Submitting Author unless you are acting as an employee on behalf of your employer or a postgraduate student of an affiliated institution which is paying any applicable article publishing charge ("APC") for Open Access articles. Where the Submitting Author wishes to make the Work available on an Open Access basis (and intends to pay the relevant APC), the terms of reuse of such Open Access shall be governed by a Creative Commons licence – details of these licences and which Creative Commons licence will apply to this Work are set out in our licence referred to above.

Other than as permitted in any relevant BMJ Author's Self Archiving Policies, I confirm this Work has not been accepted for publication elsewhere, is not being considered for publication elsewhere and does not duplicate material already published. I confirm all authors consent to publication of this Work and authorise the granting of this licence.

For peer review only

Disease Burden of Ageing, Sex and Regional Disparities, and Health Resources Allocation: A Longitudinal Analysis of 31 Provinces in Mainland China

Title page

Shu Chen MSc [1,2], Yafei Si MA [1,2], Katja Hanewald PhD [1,2], Bingqin Li PhD [3], Hazel Bateman PhD [1,2], Xiaochen Dai MSc, MPH, PhD [4], Chenkai Wu PhD [5], Shenglan Tang MD, PhD * [5,6,7]

1 Australian Research Council Centre of Excellence in Population Ageing Research (CEPAR), University of New South Wales, Sydney, Australia.
2 School of Risk & Actuarial Studies, University of New South Wales, Sydney, Australia.
3 Social Policy Research Centre, University of New South Wales, Sydney, Australia.
4 The Institute of Health Metrics and Evaluation, Seattle, US.
5 Global Health Research Center, Duke Kunshan University, Kunshan, China.
6 Duke Global Health Institute, Duke University, Durham, US.
7 SingHealth Duke-NUS Global Health Institute, Duke-NUS, Singapore.

Corresponding to:

Shenglan Tang, MD, PhD
Email: shenglan.tang@duke.edu

Address:
Duke Global Health Institute
310 Trent Drive, Durham
NC 27710, United States

For peer review only - http://bmjopen.bmj.com/site/about/guidelines.xhtml

Abstract

Objectives

To measure the disease burden of ageing based on age-related diseases (ARD), the sex and regional disparities, and the impact of health resources allocation on the burden in China.

Design

A national comparative study based on Global Burden of Diseases (GBD) study estimates and China's routine official statistics.

Setting and participants

Thirty-one provinces of mainland China were included for analysis in the study. No individuals were involved.

Methods

We first identified the ARDs and calculated the disability-adjusted life-years (DALYs) of ARDs in 2016. We assessed the ARD burden disparities by province and sex, and calculated the provincial ARD burden-adjusted age. We assessed historical changes between 1990 and 2016. Fixed-effects regression models were adopted to assess the impact of health expenditures and health workforce indicators on the ARD burden in 2010-2016.

Results

In 2016, China's total burden of ARDs was 15703.7 DALYs (95% UI: 12628.5, 18406.2) per 100,000 population. Non-communicable diseases (NCDs) accounted for 91.9% of the burden. There were significant regional disparities. The leading five youngest provinces were Beijing, Guangdong, Shanghai, Zhejiang and Fujian, located in east coast of China with an ARD burden-adjusted age below 40. After standardising the age structure, western provinces including Tibet, Qinghai, Guizhou, and Xinjiang had the highest burden of ARDs. Males were disproportionately affected by ARDs. China's overall age-standardised ARD burden had decreased since 1990, females and eastern provinces experienced the largest decline. Regression results showed the urban-rural gap in health workforce density was positively associated with the ARD burdens.

Conclusion

Chronological age alone does not provide a strong enough basis for appropriate ageing resource planning or policymaking. In China, concerted efforts should be made to reduce the ARDs burden and its disparities. Health resources should be deliberately allocated to western provinces which face the greatest health challenges due to future ageing.

Strengthens and limitations of the study

- This is the first study that assesses the age-related diseases burden and its longitudinal changes in 31 provinces of mainland China from 1990 to 2016.
- The study generates high-quality evidence on disparities in ARD burdens across provinces, sexes, and disease categories in China.
- The study adopts robust fixed-effects regression models to assess the impact of health expenditures and human resources for health indicators on the ARD burden.
- Although the study has used the best data sources available for the analysis, future research could generate more up-to-date results once more recent data become available.

For peer review only - http://bmjopen.bmj.com/site/about/guidelines.xhtml

Introduction

The world is rapidly ageing due to increased life expectancy and decreased fertility rates.[1] Health is a key factor in determining whether population ageing means more opportunities or challenges to society.[2] Although lifespans have increased substantially worldwide, it is unclear whether healthspans, i.e. healthy and morbidity-free lifespans, have improved similarly.[2–9] It is therefore essential to measure the interactions between ageing and health for a clearer understanding of the disease burden of ageing. Numerous metrics have been developed to measure population ageing and health for resource planning. Traditionally, the change of chronological age and age structure have been used to assess ageing based on demographic metrics such as life expectancy or the percentage of the population over a certain age threshold.[10] Another set of metrics assesses functional status through indicators such as frailty,[11,12] disability,[13–15] cognitive function,[16] and intrinsic capacity.[17,18] Health status is typically assessed based on biomarkers, self-reported health status[19,20] and epidemiological indicators of different diseases such as incidence, prevalence, mortality, and disability-adjusted life years (DALYs). Some metrics measure ageing while also taking health status into account. These metrics include healthy life years (HLYs), healthy life expectancy (HALE), and biological age. However, these metrics either measure ageing and health separately or cannot provide disease-specific burden information to guide resource planning.

To complement the existing metrics and measure the population-level disease burden of ageing, Chang et al. developed a novel metric called the age-related disease (ARD) burden.[21] They defined ARDs as diseases with incidence rates that increased quadratically with age. The researchers used estimates from the Global Burden of Diseases, Injuries, and Risk Factors Study (GBD) 2017 to measure and compare ARD burdens of 195 countries from 1990 to 2017. They found that non-communicable diseases (NCDs) were the primary contributor to ARDs, and there were large variations in ARD burdens across countries. Of the 195 countries, Switzerland had the lowest ARD burden, while China ranked 75th and Vanuatu had the highest. The researchers found that the age-standardised ARD burden dropped globally between 1990 and 2017 due to lower disease severity and case fatality rates. Hu et al. assessed China's ARD burden based on the GBD 2017 study estimates at the national level. The researchers consistently found that NCDs are the primary contributor to ARDs in China. The age-standardised ARD burdens also decreased between 1990 and 2017, and the magnitude of ARD decrease was larger among women than men.[22] These findings emphasise the inadequacy of using chronological age alone to inform resource planning and policy design. They confirm the importance of considering health status and disease severity within the context of ageing and the need for concerted efforts to address regional disparities, especially for regions or countries with large development inequalities.[21,22]

Many previous studies have assessed whether and how health resource allocation can impact health outcomes.[23–29] Health workforce density and total health expenditures per capita have been widely used as key proxies for health resources. A large body of high-quality evidence supports the view that a higher density of health workforce can help improve health outcomes such as life expectancy, infant mortality rate, under-five mortality rate, and maternal mortality rate.[23,24] Evidence further shows heterogeneity among different types of health workforce in terms of how they impact health outcomes.[23,24] The impact of health expenditures on health outcomes is mixed.[26–29] For example, evidence shows that an increase in health expenditures per capita can positively impact health outcomes, including life expectancy, under-five mortality, and maternal mortality in Sub-Saharan African countries,[29] though a similar study did not find supporting evidence in Organisation for Economic Co-operation and Development (OECD) countries.[26] Another study found a significant association between higher health expenditures per capita and lower infant mortality in 15 European Union (EU) countries, but only marginal increases in life expectancy.[28]

For peer review only - http://bmjopen.bmj.com/site/about/guidelines.xhtml

China is a large, rapidly ageing country with uneven economic and health development across provinces. In 2020, 264 million of China's inhabitants (18.7%) were over 60 years old, ranking first globally by this metric, and this number is projected to double by 2050.[1,30] Generally, in China, the economy is most developed in eastern provinces, followed by the central and western provinces. For example, in 2020, the gross domestic product (GDP) per capita in Shanghai, a municipality located on China's east coast, was 4.3 times higher than that of Gansu, a province in western China.[30] Regional disparities in health development are similar in scale; provinces in the eastern region generally perform best, followed by the central and western provinces.[31] For example, among males in 2015, the average healthy life expectancy was estimated to be around 78 years in Beijing, Tianjin, and Shanghai, compared to 69 years in Qinghai, Tibet, and Yunnan, located in western China.[32] China is estimated to attain 12 of the 28 health-related Sustainable Development Goals (SDGs) indicators by 2030, and the country's eastern provinces are estimated to achieve a higher number.[33] Therefore, understanding the disparities in disease burden of ageing across provinces and the impact of health resource allocation on the burden is crucial for China's central and provincial governments to plan for an ageing society. Nevertheless, no study has yet been conducted for this purpose. To fill the evidence gap, this study aims to: 1) measure the burden of ARDs in China at the subnational level, examining disparities across provinces and by sex, 2) assess changes in ARD burden from 1990–2016, and 3) explore how health resource allocation impacts the ARD burden. We also examined the difference by disease group whenever possible.

Methods

Overview

This study used estimates from the GBD study to calculate and analyse the ARD burden in China. The GBD team have updated their estimates annually since 2015 (except for 2018), with improvements on disease causes, risk factors, data input sources and modelling strategies to obtain updated and robust estimates. The most recent publicly released estimates were GBD 2019.[34,35] We used GBD 2019 study estimates to select ARDs and calculate the total national burden considering its timeliness. We conducted an analysis of the ARD burden at the subnational level based on estimates of the GBD 2016 study, as they were the most recent subnational estimates of China our team could access. However, we did not find significant changes in the causes and estimates that could impact the robustness of the study findings after careful comparison. The detailed methodology of the GBD 2016 and 2019 studies has been published elsewhere.[35,36] The GBD study classified the causes hierarchies into four levels, each with increased specificity.[37] Level One consists of three general causes: communicable diseases, NCDs, and injuries. Levels Two, Three, and Four further divide these causes into subgroups.[37] The data related to provincial health expenditures, human resources for health, and economic and demographic indicators were obtained from China Statistical and Health Statistical Yearbooks, providing data from 2010 to 2016 for regression analysis.

The geographical units of analysis are provincial-level jurisdictions in mainland China, including 22 provinces, five autonomous regions, and four municipalities (hereafter referred to as 31 provinces).[30] Our study complies with the guidelines for Accurate and Transparent Health Estimates Reporting (known as GATHER) statement and we provided the GATHER checklist in the appendix.

ARD selection and burden measurement

Considering data availability, we followed the definition developed by Chang et al. to measure ARDs as diseases with incidence rates that increased quadratically with age among the adult population.[21] Our selection of diseases focused on GBD Level Three causes, and the detailed selection methods have been

For peer review only - http://bmjopen.bmj.com/site/about/guidelines.xhtml

published elsewhere.[21] To include a comprehensive list of diseases, we focused on the population aged 15 years and older.

The ARD burden was measured by calculating the sum of the DALYs of the identified ARDs in the entire population. DALYs are defined as the sum of the years of life lost due to premature mortality and the years lived with a disability due to a disease or health condition. We calculated the provincial ARD burden of non-communicable diseases (NCDs) to approximate the total ARD burden to analyse regional disparities, as previous studies showed NCDs contributed to over 80% of the ARD burden,[21,22] and we could only access the subnational estimates of NCDs. We reported the ARD burden rate, measured as DALYs per 100,000 population, to facilitate comparisons. To explore the burden composition by disease category, the ARD burden was further stratified and analysed at the second level of GBD causes within NCDs and by sex when appropriate. We also calculated the percentage decrease in the ARD burden to assess the historical change and clustered the results by disease category and region for comparative analysis (please see appendix Table S1 for the administrative divisions).

The ARD burden-adjusted age was defined as the equivalent age measured by the ARD burden. We estimated the ARD burden-adjusted age of each province to facilitate an intuitive assessment and comparison across provinces. We first identified the national ARD burden-adjusted age based on its age-specific burden rate. We selected the two five-year age groups with closest burden rate as the national average, and calculated the national average age by assuming a linear increase of the ARD burden within each five-year age group. Afterwards, the provincial ARD burden-adjusted age was calculated, assuming each province shared the same burden rate per age as the national average (appendix for calculation details). A younger ARD burden-adjusted age, therefore, implied a lower ARD burden.

To compare the historical change in the ARD burden, we applied age standardisation to the calculation using the Institute for Health Metrics and Evaluation (IHME) standard population age structure when necessary (appendix Table S2 for the standard population structure). The 95% uncertainty intervals (UI), generated by the GBD study team as the 25th and 975th ordered 1000 random draw values of the posterior distribution,[36,38] were also presented for the ARD burden estimates.

Analysing the impact of health resource allocation on the ARD burden

We explored the impact of health resource allocation on the ARD burden using a panel data analysis approach. All equations were estimated with a log-linear functional form to enable unit-free comparisons of coefficients. To understand the underlying reasons for burden shifts over time, we used the age-standardised ARD burden rate as the dependent variable. Health expenditures and health workforce density were adopted as proxy measures for health resources. Total health expenditure per capita was the key independent variable used to measure health expenditures.[26,27] The key independent variables used to measure health workforce density were three separate sets of indicators: total health professional density, licensed doctor density, and licensed nurse density, all per 1,000 population. Health professionals included licensed doctors (clinical, dental, public health, and traditional Chinese medicine), licensed nurses, pharmacists, clinical laboratory technicians, and radiologists.[39] We chose three sets of indicators to measure health workforce density because 1) together, they accurately represent the distribution of China's health personnel resources; 2) they are widely used in published literature and heterogeneity exists in terms of their impact on health outcomes;[23,24] 3) data stratified by province and by urban and rural areas are available for all three indicators. We ran three separate regression models to include the three health workforce density indicators respectively.

For peer review only - http://bmjopen.bmj.com/site/about/guidelines.xhtml

We included GDP per capita, sex, and education as covariates in the regression models to account for the major socio-economic determinants of the population health burden. China has made remarkable achievements over the past decade in reducing the health disparities between urban and rural residents, especially through improving maternal and child health and extending health insurance coverage, among others, for its rural residents.[40] However, there is still a noticeable urban-rural gap in health development, including access to quality health services, health workforce quantity and quality, and health outcomes.[41–43] Therefore, to account for the large gaps in urban-rural development and health across China, we included the percentage of people residing in urban areas (to measure the process of urbanization) and the ratio of urban-rural health workforce density in the model as covariates. The model also included time dummies (to control period effects), province fixed effects and an error term (see appendix for model details). We further explored the correlation of key variables to assess whether there was multicollinearity that could undermine the robustness of the estimates for specific variables in the regression model (appendix, Table S3 and S4). Standard errors were clustered at the provincial level. Province was the unit of analysis, and a fixed-effects estimator was used to remove the potential endogeneity from time-invariant omitted variables.

Since provincial-level data on health expenditures per capita and health workforce density were only largely available from 2010 onwards, our panel dataset included data from 2010 to 2016. We performed log-linear interpolation to obtain annual estimates of the ARD rate from 2010 to 2016 as the GBD study only provides estimates in five-year intervals.[38] The health expenditures per capita in 2010 are missing for seven provinces: Shanghai, Hainan, Sichuan, Tibet, Shaanxi, Qinghai, and Ningxia. Only data of Tibet is missing in 2011. Therefore, after testing the robustness of the linear increase assumption, we imputed the missing data, assuming a linear increase in per capita health expenditures between 2010 and 2016. All analyses were performed in Stata 16·0 (Stata Corp LLP, College Station, TX, USA).

Patient and public involvement statement

Patients or the public were not involved in the design, conduct, reporting, or dissemination of this research.

Results

A total of 58 causes out of 169 causes (34·3%) at level three were selected as ARDs in China (Table 1). Nine of the 58 causes were infectious diseases (15·5%), 47 were NCDs (81·0%), and two were injuries (3·5%). In 2016, the total burden of ARDs in China was 15703.7 DALYs (95% UI: 12628.5, 18406.2) per 100,000 population. NCDs accounted for 91·9% of the total burden. Among the NCDs, cardiovascular diseases (CVDs), neoplasms, chronic respiratory diseases (CRDs), sense organ diseases and neurological disorders were the five leading contributors to the ARD burden.

Table 1. Summary of age-related diseases in China by category

GBD Causes at Level 1	GBD Causes at Level 2	GBD Causes at Level 3
**Infectious diseases**	Respiratory infections and Tuberculosis	Tuberculosis, diarrheal diseases, lower respiratory diseases
	Other infectious diseases	Meningitis, encephalitis, tetanus, varicella, and herpes zoster
	Neglected tropical diseases and malaria	Cysticercosis, trachoma

For peer review only - http://bmjopen.bmj.com/site/about/guidelines.xhtml

**NCDs**	Neoplasms	Oesophageal cancer, stomach cancer, liver cancer, tracheal, bronchus, and lung cancer, prostate cancer, colon and rectum cancer, lip and oral cavity cancer, other pharynx cancer, gallbladder and biliary tract cancer, pancreatic cancer, malignant skin melanoma, non-melanoma skin cancer, bladder cancer, brain and central nervous system cancer, mesothelioma, Hodgkin lymphoma, non-Hodgkin lymphoma, multiple myeloma, leukaemia, other malignant neoplasms
	Cardiovascular Diseases	Ischemic heart disease, stroke, hypertensive heart disease, cardiomyopathy, myocarditis, atrial fibrillation and flutter, peripheral artery disease, endocarditis
	Chronic respiratory diseases	Chronic obstructive pulmonary disease, asthma, and interstitial lung disease
	Digestive Diseases	Paralytic ileus and intestinal obstruct, vascular intestinal disorders, pancreatitis
	Neurological disorders	Alzheimer's disease and other dementias, Parkinson's disease, idiopathic epilepsy, other neurological disorders
	Diabetes and kidney diseases	Acute glomerulonephritis, chronic kidney disease
	Skin and subcutaneous diseases	Fungal skin diseases, pruritus, decubitus ulcer, and other skin and subcutaneous diseases
	Sense organ diseases	Age-related and other hearing loss, other sense organ diseases, blindness, and vision loss
	Musculoskeletal disorders	Low back pain
**Injuries**	Unintentional injuries	Falls, drowning

Data source: GBD 2019

Significant regional and sex disparities in ARD burdens existed in China. Generally, the eastern provinces had the lowest crude burden rates, while north-eastern and several western provinces bore the highest ones (Figure 1). After standardizing the age structure, we noted tiered gaps in the age-standardised ARD burden among the eastern, central, and western provinces. Shanghai, Beijing, Zhejiang, and Jiangsu had the lowest burdens, while the burden of Tibet, Qinghai, Guizhou, and Xinjiang were the highest. There were salient disparities between the sexes, with age-standardised ARD burden rates being 61.2% higher among males than females on average. This disparity was most apparent in neoplasms, where the burden rate was 1·5 times higher among males. We observed an inversion of this trend only among neurological disorders, where the burden rate was 10.9% higher among females.

In 2016, China's crude ARD burden-adjusted age was 50·38 years (95% UI: 49.91. 50.91), following the same regional disparity pattern as measured by DALYs (Figure 2). Beijing, Guangdong, Shanghai, Zhejiang and Fujian, all located in eastern China, were the youngest, with a crude ARD burden-adjusted age below 40 years. In comparison, Sichuan, Heilongjiang, Chongqing, Jilin and Liaoning were the oldest, with the crude age close to or above 60 years. However, after the age structure across provinces was standarised, there were notable changes in the ARD burden-adjusted ages among several western provinces, especially for Tibet, Qinghai, Guizhou and Xinjiang. These four regions were the oldest by the standardised age , at

For peer review only - http://bmjopen.bmj.com/site/about/guidelines.xhtml

72.70 (95% UI:60.53, 85.55), 66.82 (95% UI:59.36, 74.03), 60.53 (95% UI: 53.76, 63.56) and 60.06 (95% UI: 53.00, 66.88) years respectively. Shanghai, Beijing, and Zhejiang still had the youngest age-standardised ARD burden-adjusted ages, all of which were below 40 years.

Between 1990 and 2016, China's age-standardised ARD burden had decreased despite the increasing life expectancy, with a national average decline of 34·6% compared to the 1990 burden. We found regional disparities in the magnitude of this decline. The largest decline was seen in the eastern provinces, followed by the central and western provinces, with the average ARD burdens declining by 35·2%, 33·9%, and 32·7%, respectively (Figure 3). Breaking this down by province, Fujian experienced the largest decline in the age-standardised ARD burden (42·2%), followed by Zhejiang (42·0%), and Beijing (40·7%). Guangxi, Qinghai, and Guizhou experienced the smallest ARD burden decline in the same period, at 27·8%, 27·6%, 27·5%, respectively. The ARD burden declined more sharply in females (41·4%) than males (29·8%). Between 1990 and 2016, the ARD burden had decreased for CVDs, neoplasms, and CRDs, stayed almost unchanged for sense organ diseases and increased for neurological disorders (Figure 4). CRDs experienced the sharpest decline (67.9%), followed by neoplasms (30.5%) and CVDs (26·8%). However, the burden of neurological disorders increased by 5% during the same period.

China's health investment grew between 2010 and 2016. The country's total health expenditures per capita increased from 233·9 USD (exchange rate, 1 USD≈6·37 CNY) in 2010 to 526·2 USD in 2016. The total health professional density (the number of total health professionals per 1,000 people) increased by 38·6% from 4·4 in 2010 to 6·1 in 2016. In the same period, the licensed doctor density increased by 27·8%, while the licensed nurse density increased by 66·7% (Appendix for Table S5).[39,44] We report the regression results in Table 2. All coefficients of the health resource variables had the expected sign. The model results showed neither higher health expenditures per capita nor higher health workforce density could significantly lead to lower ARD burden, ceteris paribus. However, we found that the existing urban-rural gap in health workforce density was positively associated with the ARD burden, significant at the 10% level for all three indicators. A 100% increase in the urban-rural ratio in total health professional density, licensed doctor density, and licensed nurse density led to 2·37% (95% CI: -0·41, 5·15), 2·10% (95% CI: -0·27, 4·46), and 2.02% (95% CI: -0·34, 4·39) increases in the ARD burden respectively, ceteris paribus.

For peer review only

For peer review only - http://bmjopen.bmj.com/site/about/guidelines.xhtml

Table 2. Regression model results: Assessing the impact of health resources on age-standardised age-related diseases burden in mainland China, 2010–2016

Independent Variables	Model 1 (Total health professional density)		Model 2 (Licensed doctor density)		Model 3 (Licensed nurse density)	
	Coefficient (β)	95% CI	Coefficient (β)	95% CI	Coefficient (β)	95% CI
Total health expenditures per capita	-2.29	(-6.46, 1.88)	-2.02	(-6.18, 2.14)	-2.32	(-6.56, 1.91)
Health professional density	-1.34	(-3.48, 0.79)	..	..	..	..
Health professional density: urban-rural ratio	2.37*	(-0.41, 5.15)	..	..	..	..
Licensed doctor density	..	..	-0.93	(-3.30, 1.44)	..	..
Licensed doctor density: urban-rural ratio	..	..	2.10*	(-0.27, 4.46)	..	..
Licensed nurse density	..	..	..	..	-1.33	(-3.61, 0.94)
Licensed nurse density: urban-rural ratio	..	..	..	..	2.02*	(-0.34, 4.39)
GDP per capita	-0.16	(-5.09, 4.77)	-1.00	(-6.34, 4.33)	-0.34	(-5.57, 4.89)
Population living in urban areas (%)	1.82	(-15.60, 19.24)	1.83	(-15.13, 18.80)	3.03	(-14.95, 21.01)
Female (%)	-0.60	(-2.50, 1.30)	-0.87	(-2.66, 0.92)	-0.83	(-2.80, 1.14)
≥ junior middle school education (%)	3.25	(-1.66, 8.16)	2.30	(-2.25, 6.84)	3.03	(-1.72, 7.79)
adj. R^2	0.93	..	0.93	..	0.93	..

Data sources: GBD 2016, Statistical Yearbook of China (2011–2017), and the Health Statistical Yearbook of China (2011–2017)

Notes:
All dependent and independent variables were transformed into natural logarithms for regressions except for the time dummies. The independent variables of interest and covariates were rescaled by divided by 100. The coefficients can be interpreted as follows: every 100% increase in X can lead to a β% increase on Y.
Model 1 used total health professional density (per 1,000 population) as a proxy for human resources for health.
Model 2 used licensed doctor density as a proxy (per 1,000 population) for human resources for health.
Model 3 used licensed nurse density as a proxy (per 1,000 population) for human resources for health.
Significance level: *(p<0·10); **(p<0·05); ***(p<0·01)

For peer review only - http://bmjopen.bmj.com/site/about/guidelines.xhtml

Discussion

Healthy ageing has become a focal discussion topic in today's fast-ageing world, as good health in advanced age can provide continued opportunities for social and personal development.[2] The World Health Organisation officially proposed the concept of healthy ageing in 2015 and published the Global Strategy and Action Plan on Ageing and Health (2016–2020) in 2016. [2,45] The Decade of Healthy Ageing: Plan of Action (2020–2030) was issued in 2020 and asked for a whole-of-government and whole-of-society response to healthy ageing.[46] In response to this international call and the needs of the world's largest ageing population, China has actively worked to address the shifts catalysed by an ageing society and promote healthy ageing.[47–49] We conducted this study to generate high-quality evidence to help China and similar countries be better equipped to face health challenges in ageing societies. To our knowledge, this is the first study that assesses the disease burden of ageing and its longitudinal changes in 31 provinces of mainland China from 1990 to 2016. The analysis explored disparities in ARD burdens across regions, sexes, and disease categories and used panel regression models to examine the impact of health resource indicators on the ARD burden.

Our findings underscore several key messages on the disease burden of ageing in China. First, NCDs account for over 90% of the country's total ARD burden. CVDs, neoplasms, CRDs, sense organ diseases and neurological disorders were the top five contributors to the ARD burden. Second, there are significant regional disparities in ARD burdens. The results show tiered differences in the ARD burden, with the lowest rates in the eastern provinces, followed by the central provinces, and the highest rates in the north-eastern and western provinces. Notably, the crude ARD burden rate in Sichuan was 2.1 times that of Beijing in 2016, equal to a 34·14-year age gap measured by the ARD burden-adjusted age defined in this study. Third, several western provinces will face daunting ARD-induced challenges as their populations begin to age. After age structure standardisation, Tibet, Qinghai, Guizhou, and Xinjiang had the four highest ARD burdens. However, in 2016, less than 10% of each of these provinces' populations were aged 65 years and older, compared to a national average of 10.8%.[30] Fourth, males are disproportionally affected by ARDs except for neurological disorders. Fifth, the overall age-standardised ARD burden has been decreasing since 1990, though the largest decline was observed in the eastern provinces, followed by the central and western provinces. CRDs experienced the largest decline in age-standardised burden by disease category, followed by neoplasms and CVDs. The burden rate of sense organ diseases had stayed almost unchanged since 1990. The burden of neurological disorders had increased since 1990, albeit by a small magnitude. Lastly, our results suggest that greater investment in health to reduce the urban-rural gap in health workforce could help lower China's ARD burden.

Our study findings are consistent with previous studies that assessed the ARD burden globally and in China.[21,22] There is overall support for the idea that NCDs are the chief contributor to ARDs. As has been the case across the globe, the ARD burden has continuously decreased since 1990, declining most sharply in developed countries and regions. Disparities by sex are also similar; males are more negatively affected by the ARD burden and have experienced a lower historical decline. The magnitude of the burden measured in this study is slightly different from those in other studies due to differences across GBD datasets and the populations and diseases included. Our findings are novel in that we were able to measure the ARD burden and disparities across provinces in China, and examine the impact of health resource indicators on the burden. Nevertheless, the regional disparities of age-standardised ARD burdens are in line with their health development status and similar to the disparities in estimated healthy life expectancy.[50–33] The rank of NCD burdens among the ARDs is consistent with that of the disease burden among older people in China.[34] Interestingly, we found that increasing health expenditures or workforce density does not significantly decrease the ARD burden; rather, the key is to reduce the urban-rural gap in the health workforce density. Previous evidence has indicated that increasing health workforce density

For peer review only - http://bmjopen.bmj.com/site/about/guidelines.xhtml

can improve health outcomes.[23,24] However, these studies focus on measuring health outcomes such as maternal mortality, infant, and under-five mortality as opposed to ageing-related outcomes. In addition, these studies did not control for geographic resource differences between rural and urban areas and were conducted in countries where resources were scarce. Thus, the context was quite different from that of the present study.

The Chinese State Council published *The Opinions on Strengthening Aged Care Work in the New Era* in November 2021, outlining eight important action domains for addressing ageing. This document serves as a comprehensive, strategic plan to achieve healthy and active ageing in China.[49] The findings from the current study provide timely input into policy development for the governments of China to promote healthy ageing by reducing the disease burden of ageing. First, to reiterate the findings from previous studies, chronological age alone does not provide a strong enough basis for appropriate ageing resource planning or policymaking.[21,22] In China, provinces or municipalities with older populations, such as Shanghai, Beijing, and Zhejiang, have much lower ARD burdens than some younger provinces such as Tibet and Xinjiang. This phenomenon calls for more careful consideration of underlying health burdens and potential threats to resource planning and policy design to address future ageing. Second, it is crucial to continue and strengthen NCD prevention and control efforts, especially for CVDs, neoplasms, CRDs, sense organ diseases and neurological disorders. To prevent the continued increase in the ARD burden from neurological disorders, it is imperative to allocate additional health resources for the prevention, treatment, and management of conditions such as Alzheimer's disease, dementia, Parkinson's disease, and idiopathic epilepsy. Sense organ diseases cannot be ignored, especially those leading to blindness and vision loss that can significantly damage the quality of life for the elderly. Third, the central government should continue to provide all-around support to the regions that face the greatest threat of high ARD burdens due to future ageing, especially the western provinces of Tibet, Qinghai, Guizhou, and Xinjiang to reduce regional disparities. Notably, that support should entail the establishment of a strong health workforce that can serve the local people. Fourth, aligned with the Rural Revitalization Strategic Plan issued by the State Council (2018–2022) in 2018,[51] the central and local governments should work collaboratively to strengthen the rural health system, in particular, by increasing the health workforce density to reduce the urban-rural gap.

Our team have carefully compared the difference between estimates from GBD 2016 and 2019 study to ensure the robustness of the findings from the current study. Methodologically, the GBD 2019 study generally takes a similar approach as used in the GBD 2017 study, which has enriched data input sources, modelling parameters, disease categories and upgraded calibration methods to get more accurate results as compared to the GBD 2016 study.[35,36] Our comparison results show that the crude ARD burden rate in 2016 based on estimates from GBD 2019 is slightly higher than estimates from GBD 2016, though the difference is within 8% and as low as 2% for neoplasms. Regarding the diseases included, we did not find significant changes at level three that would impact the results of the ARD burden. The findings are consistent with the comparison conducted by a previous study on the estimates generated by the GBD 2016 and 2017 study for the subnational level of China.[33] Therefore, subnational estimates of GBD 2016 can well represent the regional disparities of ARD burden in China.

Our study has several limitations. First, the subnational analysis was based on the GBD 2016 study estimates, which was not updated to 2019, as we do not have access to the GBD 2019 estimates at subnational level of China. However, this was the best data source we could access and as stated above, the results from the GBD 2016 study are robust to show regional disparities after careful comparison. Second, our provincial analysis of the ARD burden focused on NCDs due to data availability, and we did not explore the burden of specific diseases. However, as NCDs accounted for over 90% of the ARD burden

For peer review only - http://bmjopen.bmj.com/site/about/guidelines.xhtml

in China, these results can accurately represent existing regional disparities. Third, the study findings tend to underestimate the ARD burden, as GBD study estimates fail to model the interactions between diseases, ignoring the burden caused by multimorbidity. Forth, though the definition used in the current study to measure the burden of age-related diseases is novel and easy to operationalize, it is only one of the many ways to measure it.[21,52–54] Future studies can gauge the pros and cons of different measurements and choose the most appropriate one for analysis. Fifth, we had to rely on interpolation to obtain annual estimates of burden rates from 2010 to 2016 for panel data analysis, though the GBD research team used the same methods in their analysis.[38] In addition, the indicators for health resources are limited to total health expenditures and health workforce density, largely due to data availability. Other important indicators, such as the efficiency of health funds utilization, the quality of health workforce, could be explored in future research when data become available.

Conclusion

In conclusion, our study provides valuable and original insights into the disease burden of ageing in China and the existing regional disparities. Our results consistently show that an older demographic structure does not necessarily mean a heavier disease burden, and therefore chronological age alone does not provide a strong enough basis for appropriate ageing resource planning or policymaking. Continuing to invest in population health through reducing the urban-rural gap in health workforce density would be helpful in reducing the disease burden of ageing in China. In so doing, the Government of China needs to develop an effective policy and mechanism that can attract more health professionals to work in the rural and less developed areas. The governments of China, or other countries with similar demographic and disease profiles and development contexts, need to take urgent action to decrease the disease burden of ageing to create healthy ageing societies.

Contributors

SC designed the study under the supervision of KH, BL, and HB. SC extracted the data, conducted the statistical analysis in Stata, developed the tables and figures, and drafted the manuscript. SC and YS verified the data in the study. All co-authors (SC, YS, KH, BL, HB, XD, CW and ST) contributed to commenting, editing, and approval of the final manuscript. All accept responsibility for submission to the journal for publication.

Declaration of interests

All co-authors declare no conflicts of interest for this study.

Acknowledgement

We thank the Institute for Health Metrics and Evaluation (IHME) for making this study possible by sharing the GBD 2016 study estimates from China at the subnational level. We also appreciate the technical instructions from Dr. Cheng Wan (UNSW) and Mr. Lei Guo (Duke Global Health Institute) on drawing the maps.

Funding statement

The study received funding support from the Bill & Melinda Gates Foundation (OPP1148464), the UNSW, and the ARC Centre of Excellence in Population Ageing Research (CEPAR), SHARP Fund UNSW (SHARP001). The funders had no role in study design, data collection, data analysis, data interpretation, or manuscript writing.

Data availability statement

For peer review only - http://bmjopen.bmj.com/site/about/guidelines.xhtml

China's subnational-level data from GBD 2016 were obtained from the IHME under a private agreement. Researchers can apply for data access at https://www.healthdata.org/about/contact-us if interested in using the data. Other inputs data were obtained from open resources, including China Statistical Yearbook (http://www.stats.gov.cn/tjsj/ndsj/) and China Health Statistical Yearbook (http://www.nhc.gov.cn/wjw/tjnj/list.shtml). Researchers can access and download the data from these websites.

Ethics statement

Ethics approval

Not applicable.

Patient consent for publication

Not applicable.

For peer review only

For peer review only - http://bmjopen.bmj.com/site/about/guidelines.xhtml

References

1 UN Department of Economic and Social Affairs, Population Division. World population ageing, 2019 highlights. 2020.

2 Beard JR, Officer A, de Carvalho IA, *et al.* The World report on ageing and health: a policy framework for healthy ageing. *The Lancet* 2016; **387**: 2145–54.

3 Manton KG, Gu X, Lamb VL. Change in chronic disability from 1982 to 2004/2005 as measured by long-term changes in function and health in the U.S. elderly population. *Proc Natl Acad Sci* 2006; **103**: 18374–9.

4 Chatterji S, Byles J, Cutler D, Seeman T, Verdes E. Health, functioning, and disability in older adults—present status and future implications. *The Lancet* 2015; **385**: 563–75.

5 Seeman TE, Merkin SS, Crimmins EM, Karlamangla AS. Disability Trends Among Older Americans: National Health and Nutrition Examination Surveys, 1988–1994 and 1999–2004. *Am J Public Health* 2010; **100**: 100–7.

6 Crimmins EM, Beltran-Sanchez H. Mortality and Morbidity Trends: Is There Compression of Morbidity? *J Gerontol B Psychol Sci Soc Sci* 2011; **66B**: 75–86.

7 Kyu HH, Abate D, Abate KH, *et al.* Global, regional, and national disability-adjusted life-years (DALYs) for 359 diseases and injuries and healthy life expectancy (HALE) for 195 countries and territories, 1990–2017: a systematic analysis for the Global Burden of Disease Study 2017. *The Lancet* 2018; **392**: 1859–922.

8 Lafortune G, Balestat G. Trends in Severe Disability Among Elderly People: Assessing the Evidence in 12 OECD Countries and the Future Implications. Paris, 2007 DOI:10.1787/217072070078.

9 Jagger C, Gillies C, Moscone F, *et al.* Inequalities in healthy life years in the 25 countries of the European Union in 2005: a cross-national meta-regression analysis. 2008; **372**: 8.

10 UN Department of Economic and Social Affairs Population Division. World Population Ageing: 1950-2050. New York, 2017 http://globalag.igc.org/ruralaging/world/ageingo.htm (accessed Aug 12, 2021).

11 Fried LP, Tangen CM, Walston J, *et al.* Frailty in Older Adults: Evidence for a Phenotype. *J Gerontol A Biol Sci Med Sci* 2001; **56**: M146–57.

12 Searle SD, Mitnitski A, Gahbauer EA, Gill TM, Rockwood K. A standard procedure for creating a frailty index. *BMC Geriatr* 2008; **8**: 24.

13 Edemekong PF, Bomgaars DL, Sukumaran S, Levy SB. Activities of Daily Living. In: StatPearls. Treasure Island (FL): StatPearls Publishing, 2021. http://www.ncbi.nlm.nih.gov/books/NBK470404/ (accessed Aug 11, 2021).

14 Kempen GIJM, Suurmeijer TPBM. The Development of a Hierarchical Polychotomous ADL-IADL Scale for Noninstitutionalized Elders. *The Gerontologist* 1990; **30**: 497–502.

For peer review only - http://bmjopen.bmj.com/site/about/guidelines.xhtml

15 Mahoney F, Barthel D. FUNCTIONAL EVALUATION: THE BARTHEL INDEX - PubMed. *Md State Med J* 1965; **14**: 61–5.

16 Skirbekk V, Loichinger E, Weber D. Variation in cognitive functioning as a refined approach to comparing aging across countries. *Proc Natl Acad Sci* 2012; **109**: 770–4.

17 Beard JR, Jotheeswaran AT, Cesari M, Araujo de Carvalho I. The structure and predictive value of intrinsic capacity in a longitudinal study of ageing. *BMJ Open* 2019; **9**: e026119.

18 Beard J, Si Y, Chenoweth L, Hanewald K. Intrinsic Capacity: Validation of a New WHO Concept for Healthy Ageing in a Longitudinal Chinese Study. *J Gerontol A Biol Sci Med Sci* 2021; published online Aug 3. DOI:10.1093/gerona/glab226.

19 Brooks R. EuroQol: the current state of play. *Health Policy Amst Neth* 1996; **37**. DOI:10.1016/0168-8510(96)00822-6.

20 EuroQol Group. EuroQol - a new facility for the measurement of health-related quality of life. *Health Policy* 1990; **16**: 199–208.

21 Chang AY, Skirbekk VF, Tyrovolas S, Kassebaum NJ, Dieleman JL. Measuring population ageing: an analysis of the Global Burden of Disease Study 2017. *Lancet Public Health* 2019; **4**: e159–67.

22 Hu D, Yan W, Zhu J, Zhu Y, Chen J. Age-Related Disease Burden in China, 1997–2017: Findings From the Global Burden of Disease Study. *Front Public Health* 2021; **0**. DOI:10.3389/fpubh.2021.638704.

23 Anand S, Bärnighausen T. Human resources and health outcomes: cross-country econometric study. *The Lancet* 2004; **364**: 1603–9.

24 Speybroeck N, KINFU Y, Dal Poz M, Evans D. Reassessing the Relationship Between Human Resources for Health, Intervention Coverage and Health Outcomes. *Reassessing Relatsh Hum Resour Health Interv Cover Health Outcomes* 2006; : 1–14.

25 Nguyen MP, Mirzoev T, Le TM. Contribution of health workforce to health outcomes: empirical evidence from Vietnam. *Hum Resour Health* 2016; **14**: 68.

26 Panopoulou E, Pantelidis T. Convergence in per capita health expenditures and health outcomes in the OECD countries. *Appl Econ* 2012; **44**: 3909–20.

27 Bokhari FAS, Gai Y, Gottret P. Government health expenditures and health outcomes. *Health Econ* 2007; **16**: 257–73.

28 Nixon J, Ulmann P. The relationship between health care expenditure and health outcomes. Evidence and caveats for a causal link. *Eur J Health Econ HEPAC Health Econ Prev Care* 2006; **7**: 7–18.

29 Nketiah-Amponsah E. The Impact of Health Expenditures on Health Outcomes in Sub-Saharan Africa. *J Dev Soc* 2019; **35**: 134–52.

[dataset] 30 National Bureau of Statistics, China. Data from: National data, National Bureau of Statistics, China. 2020. https://data.stats.gov.cn/easyquery.htm?cn=E0103. Aug 12, 2021.

For peer review only - http://bmjopen.bmj.com/site/about/guidelines.xhtml

For peer review only

31 Zhou M, Wang H, Zeng X, *et al.* Mortality, morbidity, and risk factors in China and its provinces, 1990–2017: a systematic analysis for the Global Burden of Disease Study 2017. *The Lancet* 2019; **394**: 1145–58.

32 Li H, Hanewald K, Wu S. Healthy life expectancy in China: Modelling and implications for public and private insurance. *Ann Actuar Sci* 2021; **15**: 40–56.

33 Chen S, Guo L, Wang Z, *et al.* Current situation and progress toward the 2030 health-related Sustainable Development Goals in China: A systematic analysis. *PLOS Med* 2019; **16**: e1002975.

[dataset] 34 Global Burden of Disease Collaborative Network. Data from: Global Burden of Disease Study 2019 (GBD 2019) Results. 2020. http://ghdx.healthdata.org/gbd-results-tool. Aug 10, 2021.

35 Vos T, Lim SS, Abbafati C, *et al.* Global burden of 369 diseases and injuries in 204 countries and territories, 1990–2019: a systematic analysis for the Global Burden of Disease Study 2019. *The Lancet* 2020; **396**: 1204–22.

36 Vos T, Abajobir AA, Abate KH, *et al.* Global, regional, and national incidence, prevalence, and years lived with disability for 328 diseases and injuries for 195 countries, 1990–2016: a systematic analysis for the Global Burden of Disease Study 2016. *The Lancet* 2017; **390**: 1211–59.

37 IHME. Frequently Asked Questions. Inst. Health Metr. Eval. 2014; published online May 9. http://www.healthdata.org/gbd/faq (accessed Nov 15, 2021).

38 Vos T, Lim SS, Abbafati C, *et al.* Global burden of 369 diseases and injuries in 204 countries and territories, 1990–2019: a systematic analysis for the Global Burden of Disease Study 2019. *The Lancet* 2020; **396**: 1204–22.

[dataset] 39 Statistics and Information Center, National Health Commission, China. Data from: China Health Statistical Yearbook 2019. Beijing: Peking Union Medical College Press, 2019. http://www.nhc.gov.cn/mohwsbwstjxxzx/tjtjnj/202106/04bd2ba9592f4a70b78d80ea50bfe96e.shtml . Aug 12, 2021.

40 Meng Q, Mills A, Wang L, Han Q. What can we learn from China's health system reform? *BMJ* 2019; **365**: l2349.

41 Ma X, He Y, Xu J. Urban–rural disparity in prevalence of multimorbidity in China: a cross-sectional nationally representative study. *BMJ Open* 2020; **10**: e038404.

42 Yang D, Acharya Y, Liu X. Social health insurance consolidation and urban-rural inequality in utilization and financial risk protection in China. *Soc Sci Med* 2022; **308**: 115200.

43 Health TLP. Public health in China: achievements and future challenges. *Lancet Public Health* 2018; **3**: e456.

[dataset] 44 Statistics and Information Center, National Health Commission, China. Data from: China Health Statistical Yearbook 2011. Beijing: Peking Union Medical College Press, 2011. http://www.nhc.gov.cn/mohwsbwstjxxzx/s7967/201301/f34860a7bdb64aad9029eb860a6cf4d2.shtml. Aug 12, 2021.

For peer review only - http://bmjopen.bmj.com/site/about/guidelines.xhtml

45 Global strategy and action plan on ageing and health. https://www.who.int/publications-detail-redirect/9789241513500 (accessed Dec 3, 2021).

46 World Health Organization. UN Decade of Healthy Ageing. https://www.who.int/initiatives/decade-of-healthy-ageing (accessed Dec 3, 2021).

47 Yang W, Chang S, Zhang W, *et al.* An Initial Analysis of the Effects of a Long-Term Care Insurance on Equity and Efficiency: A Case Study of Qingdao City in China. *Res Aging* 2021; **43**: 156–65.

48 Feng Z, Glinskaya E, Chen H, *et al.* Long-term care system for older adults in China: policy landscape, challenges, and future prospects. *The Lancet* 2020; **396**: 1362–72.

49 The State Council, China. The Opinions on Strengthening Aged Care Work in the New Era. 2021; published online Nov 24. http://www.gov.cn/zhengce/2021-11/24/content_5653181.htm (accessed Dec 3, 2021).

50 Li H, Hanewald K, Wu S. Estimating Healthy Life Expectancy: A Province-by-Province Study for China. Rochester, NY: Social Science Research Network, 2017 DOI:10.2139/ssrn.3027825.

51 The State Council, China. Rural Revitalization Strategic Plan (2018-2022). 2018; published online Sept 26. http://www.moa.gov.cn/ztzl/xczx/xczxzlgh/201811/t20181129_6163953.htm (accessed Dec 3, 2021).

52 Brody JA, Schneider EL. Diseases and disorders of aging: an hypothesis. *J Chronic Dis* 1986; **39**: 871–6.

53 Brody JA, Grant MD. Age- associated diseases and conditions: Implications for decreasing late life morbidity. *Aging Clin Exp Res* 2001; **13**: 64–7.

54 Kuan V, Fraser HC, Hingorani M, *et al.* Data-driven identification of ageing-related diseases from electronic health records. *Sci Rep* 2021; **11**: 2938.

For peer review only

For peer review only - http://bmjopen.bmj.com/site/about/guidelines.xhtml

Figure legend and footnote

Figure 1. The crude and age-standardised age-related diseases burden in 31 provinces of mainland China, 2016

Data source: GBD 2016
Note: The age-related diseases burden was standardized using the IHME standard population age structure (appendix)

Figure 2. The crude and age-standardised ARD burden-adjusted ages of 31 provinces in mainland China, 2016

Data source: GBD 2016
Note: The provinces were grouped into eastern, central and western provinces from top down.
The age-related diseases burden was standardized using the IHME standard population age structure (appendix)

Figure 3. Change of the age-standardised age-related diseases burden rates by administrative region in mainland China, 1990-2016

Data source: GBD 2016
Note: The age-related diseases burden was standardized using the IHME standard population age structure (appendix)

Figure 4. Change of the age-standardised age-related diseases burden rates of the five leading ARD contributors in China, 1990-2016

Data source: GBD 2016
Note: The age-related diseases burden was standardized using the IHME standard population age structure (appendix)

For peer review only - http://bmjopen.bmj.com/site/about/guidelines.xhtml

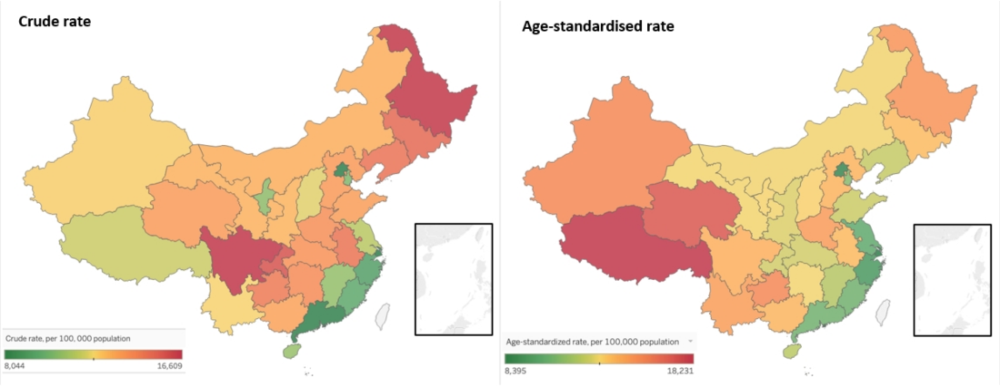

The crude and age-standardised age-related diseases burden in 31 provinces of mainland China, 2016

832x322mm (38 x 38 DPI)

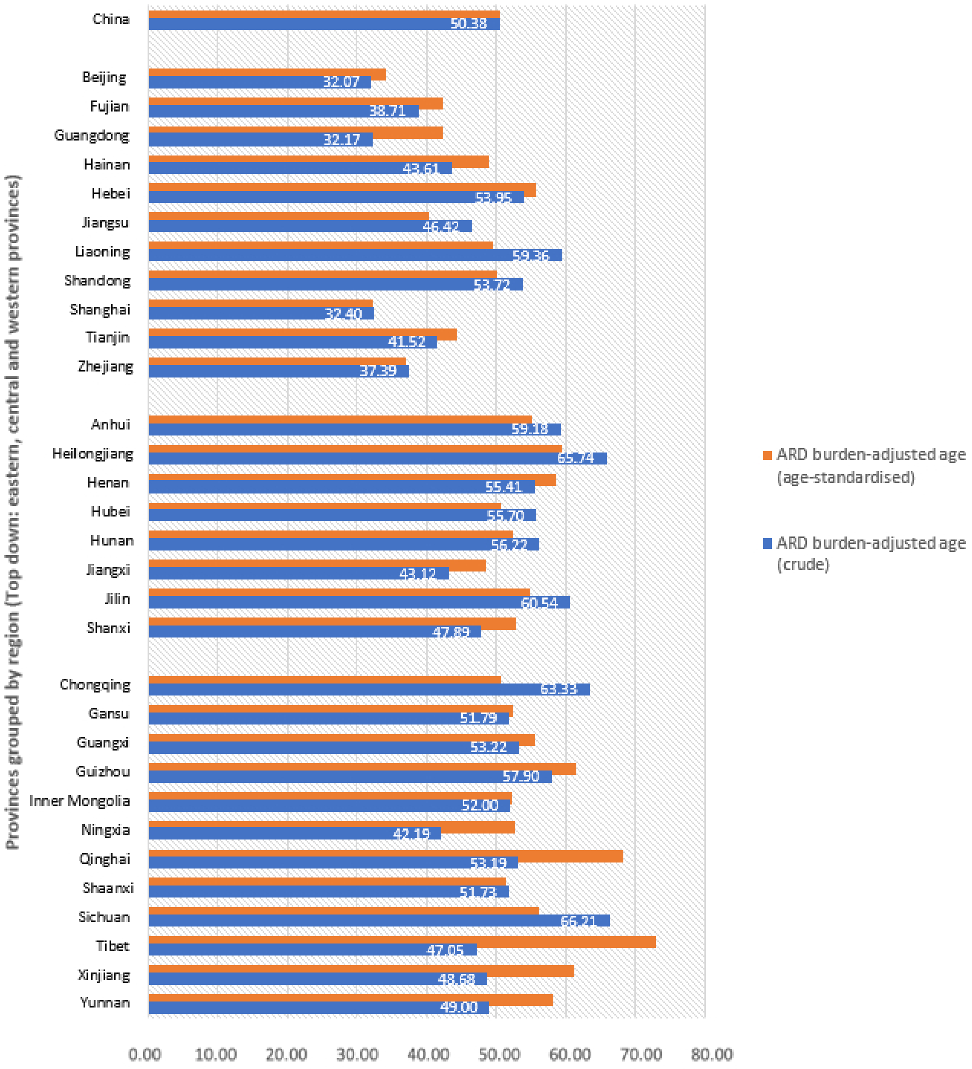

The crude and age-standardised ARD burden-adjusted ages of 31 provinces in mainland China, 2016

437x482mm (38 x 38 DPI)

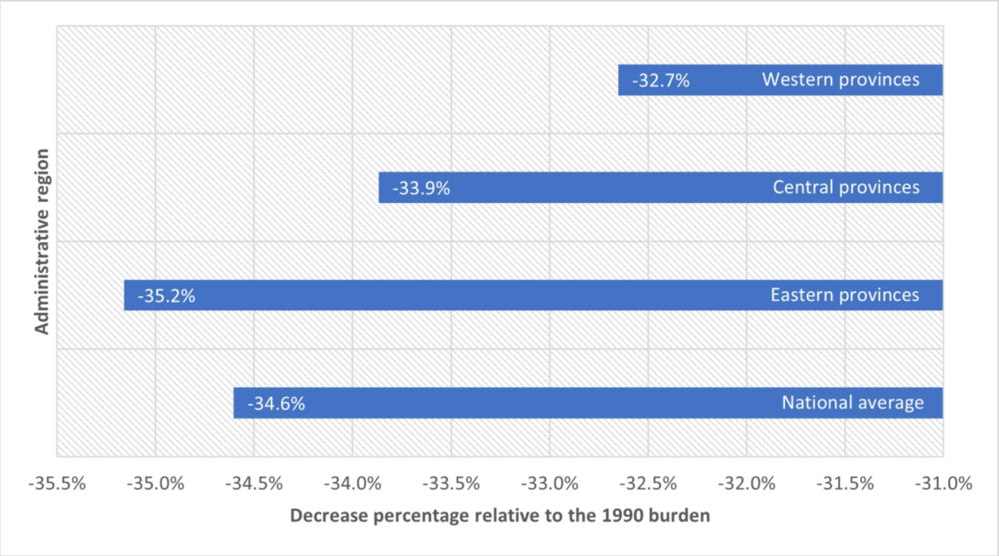

Change of the age-standardised age-related diseases burden rates by administrative region in mainland China, 1990-2016

389x216mm (130 x 130 DPI)

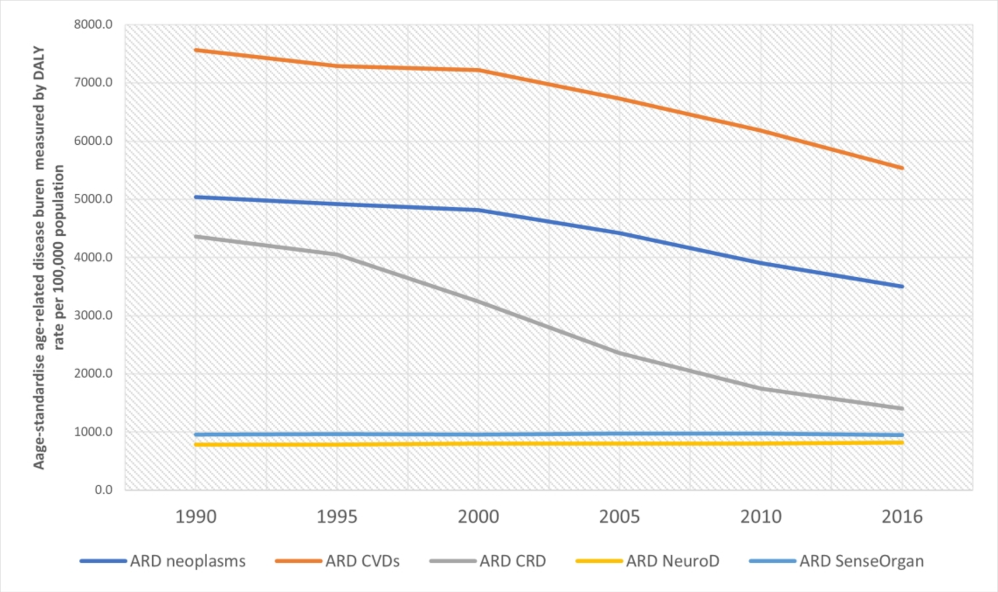

Change of the age-standardised age-related diseases burden rates of the five leading ARD contributors in China, 1990-2016

391x232mm (130 x 130 DPI)

Appendix

Table S1. Administrative division of provinces in mainland China

Administrative division (No.)	Province
Eastern provinces (11)	Beijing, Fujian, Guangdong, Hainan, Hebei, Jiangsu, Liaoning, Shandong Shanghai, Tianjin, Zhejiang
Central provinces (8)	Anhui, Heilongjiang, Henan, Hubei, Hunan, Jiangxi, Jilin, Shanxi
Western provinces (12)	Inner Mongolia, Guangxi, Chongqing, Gansu, Guizhou, Ningxia, Qinghai, Shaanxi, Sichuan, Tibet, Xinjiang, Yunnan

Table S2. IHME standard age weight

Age group	Population percentage
<5	7.2%
5–9	8.7%
10–14	8.4%
15–19	8.1%
20–24	7.8%
25–29	7.6%
30–34	7.2%
35–39	6.9%
40–44	6.4%
45–49	5.8%
50–54	5.3%
55–59	4.7%
60–64	4.1%
65–69	3.4%
70 +	8.6%

ARD burden-adjusted age calculation

First, we identified the ARD burden-adjusted age of China by comparing the national average burden rate with its burden rates by age group. We selected the two five-year age groups with closest burden rate as the national average, and calculated the national average age by assuming linear increase of the ARD burden within each five-year age group. For example, the age-related disease burden of China was 12637.2 DALYs per 100,000 population in 2016, closest to burden rates for age group of 45–49 (9316.3) and 50–54 (14163.7). Hence, the national age was calculated as:
45+12637.2/((9316.3+14163.7)/10)=50.38.

For peer review only - http://bmjopen.bmj.com/site/about/guidelines.xhtml

Second, we calculated the ARD burden-adjusted age of each province by dividing the provincial age-related disease burden rate by the unit share of the national average burden rate per age. The unit share of the national average burden rate per age was calculated by dividing the national average burden rate by the national equivalent age. For example, the age-related disease burden of Shanghai was 8,127·4 DALYs per 100,000 population. Hence, Shanghai's ARD burden-adjusted age was calculated as: 8,127·4/(12637·2/50.38)=32·40.

Regression model

$$Y_{it} = \alpha + \beta_0 healthexp_{it} + \beta_1 healthwf_{it} + \beta_2 X_{it} + \beta_3 D_t + \eta_i + \varepsilon_{it},$$

where Y_{it} is the age-standardised age-related disease burden of Province i in Year t, β_0 is the coefficient of interest, $healthexp_{it}$ is the total health expenditures per capita, and $healthwf_{it}$ is the total health professional density, licensed doctor density, or licensed nurse density (per 1,000 population) of the Province i in Year t (three separate models). X_{it} is the set of covariates controlled in the model, including GDP per capita, education, the proportion of females, the proportion of people living in urban areas, and the urban-rural health workforce density ratio. D_t represents the time dummies, η_i the province fixed effects, and ε_{it} is the error term. All variables are in log form except for the time dummies.

We also assessed the correlation between 1) the total health expenditures per capita and health workforce density and 2) the rural/urban ratio in health workforce density and the proportion of rural/urban population to check multicollinearity, before and after controlling for covariates including GDP per capita, sex and education. The results are presented below in Table S3 and Table S4.

For peer review only

Table S3. Regression model results: assessing correlation between health workforce density and total health expenditures per capita

Independent Variables	Model 1		Model 2		Model 3		Model 4		Model 5		Model 6	
	Coef (β)	P value	Coef (β)	P value	Coef (β)	P value	Coef (β)	P value	Coef (β)	P value	Coef (β)	P value
Health prof density	0.09	0.10	-0.04	0.34	··	··	··	··	··	··	··	··
Licensed doc density	··	··	··	··	0.11	0.06	0.01	0.89	··	··	··	··
Licensed nurse density	··	··	··	··	··	··	··	··	0.08	0.09	-0.02	0.51
GDP per capita	··	··	0.37	0.00	··	··	0.33	0.00	··	··	0.36	0.00
Female (%)	··	··	0.08	0.32	··	··	0.08	0.35	··	··	0.08	0.33
≥ junior mid sch educ (%)	··	··	0.08	0.50	··	··	0.06	0.58	··	··	0.08	0.50
adj. R^2	0.96	··	0.94	··	0.96	··	0.94	··	0.96	··	0.94	··

Note:
Model 1: Total health professional density as independent variable, not controlling for covariates except for time dummies
Model 2: Total health professional density as independent variable, controlling for covariates except for time dummies
Model 3: Licensed doctor density as independent variable, not controlling for covariates except for time dummies
Model 4: Licensed doctor density as independent variable, controlling for covariates except for time dummies
Model 5: Licensed nurse density as independent variable, not controlling for covariates except for time dummies
Model 6: Licensed nurse density as independent variable, controlling for covariates except for time dummies

Table S4. Regression model results: assessing correlation between urban residency and urban-rural ratio in health workforce density

Independent Variables	Model 1		Model 2	
	Coef (β)	P value	Coef (β)	P value
Population living in urban areas (%)	-0.44	0.23	-0.06	0.90
GDP per capita	··	··	-0.01	0.95
Female (%)	··	··	-0.19	0.02
≥ junior mid sch educ (%)	··	··	-0.28	0.11
adj. R^2	0.84	··	0.94	··

Note:
Model 1: Population living in urban areas as independent variable, not controlling for covariates except for time dummies
Model 2: Population living in urban areas as independent variable, controlling for covariates except for time dummies

For peer review only - http://bmjopen.bmj.com/site/about/guidelines.xhtml

Table S5. Selected statistics of the variables in the regression model, 2010 and 2016

Variables	Health expenditures per capita (CNY)*		Total health professionals density (per 1,000 population)		Urban-rural ratio in total health professionals density		GDP per capita (CNY)*		Living in urban areas (%)		Female (%)		Received at least middle school education (%)	
Province	2010	2016	2010	2016	2010	2016	2010	2016	2010	2016	2010	2016	2010	2016
Anhui	1210·5	2652·2	3·1	4·7	2·4	2·3	20888·0	39561·0	44.8%	52.0%	49·2%	48·7%	62·8%	65·5%
Beijing	4147·2	9429·7	13·6	10·8	1·9	··	73856·0	118198·0	86.2%	86.5%	48·4%	48·6%	87·6%	88·6%
Chongqing	1501·0	3492·2	3·4	5·9	1·4	2·0	27596·0	58502·0	55.0%	62.6%	49·4%	49·2%	61·0%	64·1%
Fujian	1280·1	3226·8	4·1	5·7	2·8	2·5	40025·0	74707·0	58.1%	63.6%	48·6%	49·1%	63·0%	61·7%
Gansu	1153·9	2889·2	3·7	5·2	2·0	2·2	16113·0	27643·0	37.2%	44.7%	48·9%	49·3%	56·5%	57·9%
Guangdong	1445·9	3812·5	5·3	6·0	3·2	3·2	44736·0	74016·0	66.5%	69.2%	47·8%	46·9%	73·2%	74·3%
Guangxi	1116·9	2557·0	3·6	6·0	2·2	2·2	20219·0	38027·0	41.8%	48.1%	48·0%	48·0%	62·5%	67·4%
Guizhou	946·6	2472·4	2·5	5·8	4·2	3·9	13119·0	33246·0	35.0%	44.2%	48·3%	48·4%	48·8%	54·6%
Hainan	1193·0	3306·8	4·4	6·3	2·3	3·3	23831·0	44347·0	50.5%	56.8%	47·4%	47·3%	72·1%	73·0%
Hebei	1253·8	2710·6	4·0	5·3	3·2	2·7	28668·0	43062·0	45.6%	53.3%	49·3%	48·9%	69·9%	69·7%
Heilongjiang	1580·2	3133·4	5·0	5·8	2·3	2·3	27076·0	40432·0	56.5%	59.2%	49·2%	49·5%	73·0%	73·3%
Henan	1134·0	2594·0	3·5	5·7	3·0	3·2	24446·0	42575·0	40.6%	48.5%	49·5%	49·0%	68·2%	69·8%
Hubei	1191·1	3270·6	4·2	6·5	2·1	2·2	27906·0	55665·0	51.8%	58.1%	48·6%	48·7%	69·8%	70·5%
Hunan	1042·1	2821.0	3·8	5·8	2·9	2·9	24719·0	46382·0	45.1%	52.8%	48·6%	48·9%	67·1%	71·2%

**Inner Mongolia**	1767·5	3599·7	5·1	6·8	2·7	2·6	47347·0	72064·0	56.6%	61.2%	48·1%	49·5%	69·9%	73·3%
**Jiangsu**	1566·0	4200·2	4·4	6·5	2·0	2·1	52840·0	96887·0	61.9%	67.7%	49·6%	49·6%	70·9%	71·5%
**Jiangxi**	992·0	2374·8	5·1	6·1	1·8	2·1	21253·0	40400·0	45.7%	53.1%	48·2%	48·0%	65·6%	64·1%
**Jilin**	1653·9	3501·19	5·5	6·3	2·5	2·9	31599·0	53868·0	53.4%	56.0%	49·3%	49·2%	69·8%	73·6%
**Liaoning**	1765·9	3390·9	4·7	6·6	3·0	2·6	42355·0	50791·0	64.0%	67.4%	49·4%	49·5%	75·5%	78·3%
**Ningxia**	1190·1	3730·5	4·5	6·2	4·4	5·4	26860·0	47194·0	49.8%	56.3%	48·8%	48·4%	61·3%	66·5%
**Qinghai**	1472·0	4043·1	4·7	7·6	2·0	2·0	24115·0	43531·0	46.3%	51.6%	48·2%	48·6%	49·8%	51·8%
**Shaanxi**	2040·7	3535·7	4·7	6·5	1·7	2·2	27133·0	51015·0	47.3%	55.3%	48·3%	49·5%	69·8%	71·5%
**Shandong**	1403·1	3372·7	9·7	7·4	1·3	1·6	41106·0	68733·0	50.9%	59.0%	49·4%	49·0%	67·5%	69·0%
**Shanghai**	2828·1	7596·0	5·6	6·1	2·6	3·2	76074·0	116562·0	89.3%	87.9%	48·5%	48·6%	83·6%	83·5%
**Shanxi**	1297·5	2650·3	3·4	4·8	2·7	3·0	26283·0	35532·0	49.7%	56.2%	48·6%	48·5%	74·1%	77·5%
**Sichuan**	1019·1	3238·6	3·6	6·0	2·0	2·0	21182·0	40003·0	41.8%	49.2%	49·2%	50·1%	57·2%	58·9%
**Tianjin**	2737·3	5294·2	7·1	6·1	1·4	1·1	72994·0	115053·0	80.5%	82.9%	46·6%	46·6%	80·5%	82·0%
**Tibet**	1472·0	3780·9	3·4	4·5	4·9	4·1	17027·0	35184·0	22.8%	29.6%	48·6%	49·5%	26·4%	29·6%
**Xinjiang**	1676·8	4012·9	5·7	7·1	3·2	2·4	25034·0	40564·0	43.5%	48.3%	48·7%	48·9%	66·1%	65·5%
**Yunnan**	1107·2	2754·1	3·2	5·2	3·2	3·3	15752·0	31093·0	36.8%	45.0%	48·1%	49·5%	48·3%	52·5%
**Zhejiang**	2099·0	4603·8	6·1	7·7	1·8	1·8	51711·0	84916·0	62.3%	67.0%	48·6%	47·9%	64·7%	66·0%

*CNY=Chinese Yuan, exchange rate: 1USD≈6.37CNY (Dec 3, 2020)

Data source: National Statistical Yearbook of China (2011, 2017) and National Health Statistical Yearbook of China (2011,2017)

For peer review only - http://bmjopen.bmj.com/site/about/guidelines.xhtml

Checklist of information that should be included in new reports of global health estimates

Item #	Checklist item	Reported on page #
**Objectives and funding**		
**1**	Define the indicator(s), populations (including age, sex, and geographic entities), and time period(s) for which estimates were made.	4
**2**	List the funding sources for the work.	6
**Data Inputs**		
For all data inputs from multiple sources that are synthesized as part of the study:		
**3**	Describe how the data were identified and how the data were accessed.	4 & 9
**4**	Specify the inclusion and exclusion criteria. Identify all ad-hoc exclusions.	4
**5**	Provide information on all included data sources and their main characteristics. For each data source used, report reference information or contact name/institution, population represented, data collection method, year(s) of data collection, sex and age range, diagnostic criteria or measurement method, and sample size, as relevant.	4 & 5
**6**	Identify and describe any categories of input data that have potentially important biases (e.g., based on characteristics listed in item 5).	4 & 5 & 9
For data inputs that contribute to the analysis but were not synthesized as part of the study:		
**7**	Describe and give sources for any other data inputs.	4 & 5 & 9
For all data inputs:		
**8**	Provide all data inputs in a file format from which data can be efficiently extracted (e.g., a spreadsheet rather than a PDF), including all relevant meta-data listed in item 5. For any data inputs that cannot be shared because of ethical or legal reasons, such as third-party ownership, provide a contact name or the name of the institution that retains the right to the data.	9 and appendix
**Data analysis**		
**9**	Provide a conceptual overview of the data analysis method. A diagram may be helpful.	4 & 5
**10**	Provide a detailed description of all steps of the analysis, including mathematical formulae. This description should cover, as relevant, data cleaning, data pre-processing, data adjustments and weighting of data sources, and mathematical or statistical model(s).	4 & 5
**11**	Describe how candidate models were evaluated and how the final model(s) were selected.	4 & 5
**12**	Provide the results of an evaluation of model performance, if done, as well as the results of any relevant sensitivity analysis.	7 & 15 & 22 & 23
**13**	Describe methods for calculating uncertainty of the estimates. State which sources of uncertainty were, and were not, accounted for in the uncertainty analysis.	5
**14**	State how analytic or statistical source code used to generate estimates can be accessed.	5 & 6
**Results and Discussion**		
**15**	Provide published estimates in a file format from which data can be efficiently extracted.	12-15
**16**	Report a quantitative measure of the uncertainty of the estimates (e.g. uncertainty intervals).	6,7,15
**17**	Interpret results in light of existing evidence. If updating a previous set of estimates, describe the reasons for changes in estimates.	6-9
**18**	Discuss limitations of the estimates. Include a discussion of any modelling assumptions or data limitations that affect interpretation of the estimates.	8-9

For peer review only - http://bmjopen.bmj.com/site/about/guidelines.xhtml

This checklist should be used in conjunction with the GATHER statement and Explanation and Elaboration document, found on gather-statement.org

For peer review only - http://bmjopen.bmj.com/site/about/guidelines.xhtml

BMJ Open

Disease Burden of Ageing, Sex and Regional Disparities, and Health Resources Allocation: A Longitudinal Analysis of 31 Provinces in Mainland China

Journal:	*BMJ Open*
Manuscript ID	bmjopen-2022-064641.R2
Article Type:	Original research
Date Submitted by the Author:	14-Oct-2022
Complete List of Authors:	Chen, Shu; UNSW, School of Risk and Actuarial Studies; UNSW, ARC Centre of Excellence in Population Ageing Research (CEPAR)<br>Si, Yafei; University of New South Wales, School of Risk and Actuarial Studies; University of New South Wales, ARC Centre of Excellence in Population Ageing Research (CEPAR)<br>Hanewald, Katja; University of New South Wales, School of Risk and Actuarial Studies; University of New South Wales, ARC Centre of Excellence in Population Ageing Research (CEPAR)<br>Li, Bingqin; University of New South Wales, Social Policy Research Center<br>Bateman, Hazel; University of New South Wales, School of Risk and Actuarial Studies; University of New South Wales, ARC Centre of Excellence in Population Ageing Research (CEPAR)<br>Dai, Xiaochen; Institute of Health Metrics and Evaluation<br>wu, chenkai; Duke Kunshan University, Global Health Research Center<br>Tang, Shenglan; Duke Kunshan University, Global Health Research Center; Duke University, Duke Global Health Institute
**Primary Subject Heading**:	Health policy
Secondary Subject Heading:	Public health, Epidemiology, Geriatric medicine
Keywords:	Health policy < HEALTH SERVICES ADMINISTRATION & MANAGEMENT, EPIDEMIOLOGY, Health economics < HEALTH SERVICES ADMINISTRATION & MANAGEMENT, PUBLIC HEALTH

SCHOLARONE™
Manuscripts

I, the Submitting Author has the right to grant and does grant on behalf of all authors of the Work (as defined in the below author licence), an exclusive licence and/or a non-exclusive licence for contributions from authors who are: i) UK Crown employees; ii) where BMJ has agreed a CC-BY licence shall apply, and/or iii) in accordance with the terms applicable for US Federal Government officers or employees acting as part of their official duties; on a worldwide, perpetual, irrevocable, royalty-free basis to BMJ Publishing Group Ltd ("BMJ") its licensees and where the relevant Journal is co-owned by BMJ to the co-owners of the Journal, to publish the Work in this journal and any other BMJ products and to exploit all rights, as set out in our licence.

The Submitting Author accepts and understands that any supply made under these terms is made by BMJ to the Submitting Author unless you are acting as an employee on behalf of your employer or a postgraduate student of an affiliated institution which is paying any applicable article publishing charge ("APC") for Open Access articles. Where the Submitting Author wishes to make the Work available on an Open Access basis (and intends to pay the relevant APC), the terms of reuse of such Open Access shall be governed by a Creative Commons licence – details of these licences and which Creative Commons licence will apply to this Work are set out in our licence referred to above.

Other than as permitted in any relevant BMJ Author's Self Archiving Policies, I confirm this Work has not been accepted for publication elsewhere, is not being considered for publication elsewhere and does not duplicate material already published. I confirm all authors consent to publication of this Work and authorise the granting of this licence.

For peer review only

Disease Burden of Ageing, Sex and Regional Disparities, and Health Resources Allocation: A Longitudinal Analysis of 31 Provinces in Mainland China

Title page

Shu Chen MSc [1,2], Yafei Si MA [1,2], Katja Hanewald PhD [1,2], Bingqin Li PhD [3], Hazel Bateman PhD [1,2], Xiaochen Dai MSc, MPH, PhD [4], Chenkai Wu PhD [5], Shenglan Tang MD, PhD * [5,6,7]

1 Australian Research Council Centre of Excellence in Population Ageing Research (CEPAR), University of New South Wales, Sydney, Australia.
2 School of Risk & Actuarial Studies, University of New South Wales, Sydney, Australia.
3 Social Policy Research Centre, University of New South Wales, Sydney, Australia.
4 The Institute of Health Metrics and Evaluation, Seattle, US.
5 Global Health Research Center, Duke Kunshan University, Kunshan, China.
6 Duke Global Health Institute, Duke University, Durham, US.
7 SingHealth Duke-NUS Global Health Institute, Duke-NUS, Singapore.

Corresponding to:

Shenglan Tang, MD, PhD
Email: shenglan.tang@duke.edu

Address:
Duke Global Health Institute
310 Trent Drive, Durham
NC 27710, United States

For peer review only - http://bmjopen.bmj.com/site/about/guidelines.xhtml

Abstract

Objectives

To measure the disease burden of ageing based on age-related diseases (ARD), the sex and regional disparities, and the impact of health resources allocation on the burden in China.

Design

A national comparative study based on Global Burden of Diseases (GBD) study estimates and China's routine official statistics.

Setting and participants

Thirty-one provinces of mainland China were included for analysis in the study. No individuals were involved.

Methods

We first identified the ARDs and calculated the disability-adjusted life-years (DALYs) of ARDs in 2016. We assessed the ARD burden disparities by province and sex and calculated the provincial ARD burden-adjusted age. We assessed historical changes between 1990 and 2016. Fixed-effects regression models were adopted to evaluate the impact of health expenditures and health workforce indicators on the ARD burden in 2010-2016.

Results

In 2016, China's total burden of ARDs was 15703.7 DALYs (95% UI: 12628.5, 18406.2) per 100,000 population. Non-communicable diseases (NCDs) accounted for 91.9% of the burden. There were significant regional disparities. The leading five youngest provinces were Beijing, Guangdong, Shanghai, Zhejiang, and Fujian, located on the east coast of China with an ARD burden-adjusted age below 40. After standardising the age structure, western provinces, including Tibet, Qinghai, Guizhou, and Xinjiang, had the highest burden of ARDs. Males were disproportionately affected by ARDs. China's overall age-standardised ARD burden has decreased since 1990, and females and eastern provinces experienced the largest decline. Regression results showed that the urban-rural gap in health workforce density was positively associated with the ARD burdens.

Conclusion

Chronological age alone does not provide a strong enough basis for appropriate ageing resource planning or policymaking. In China, concerted efforts should be made to reduce the ARDs burden and its disparities. Health resources should be deliberately allocated to western provinces facing the greatest health challenges due to future ageing.

Strengthens and limitations of the study

- This study uses longitudinal data from 1990 to 2016 in 31 provinces of mainland China to assess the ARD burdens.
- The study generates high-quality evidence on disparities in ARD burdens across provinces, sexes, and disease categories in China.
- The study adopts robust fixed-effects regression models to assess the impact of health expenditures and human resources for health indicators on the ARD burden.
- Although the study has used the best data sources available for the analysis, future research could generate more up-to-date results once more recent data become available.

For peer review only - http://bmjopen.bmj.com/site/about/guidelines.xhtml

Introduction

The world is rapidly ageing due to increased life expectancy and decreased fertility rates.[1] Health is a critical factor in determining whether population ageing means more opportunities or challenges to society.[2] Although lifespans have increased substantially worldwide, it is unclear whether healthspans, i.e. healthy and morbidity-free lifespans, have improved similarly.[2–9] It is, therefore, essential to measure the interactions between ageing and health for a clearer understanding of the disease burden of ageing. Numerous metrics have been developed to measure population ageing and health for resource planning. Traditionally, the change of chronological age and age structure have been used to assess ageing based on demographic metrics such as life expectancy or the percentage of the population over a certain age threshold.[10] Another set of metrics assesses functional status through indicators such as frailty,[11,12] disability,[13–15] cognitive function,[16] and intrinsic capacity.[17,18] Health status is typically estimated based on biomarkers, self-reported health status[19,20], and epidemiological indicators of different diseases such as incidence, prevalence, mortality, and disability-adjusted life years (DALYs). Some metrics measure ageing while also taking health status into account. These metrics include healthy life years (HLYs), healthy life expectancy (HALE), and biological age. However, these metrics either measure ageing and health separately or cannot provide disease-specific burden information to guide resource planning.

To complement the existing metrics and measure the population-level disease burden of ageing, Chang et al. developed a novel metric called the age-related disease (ARD) burden.[21] They defined ARDs as diseases with incidence rates that increased quadratically with age. The researchers used estimates from the Global Burden of Diseases, Injuries, and Risk Factors Study (GBD) 2017 to measure and compare ARD burdens of 195 countries from 1990 to 2017. They found that non-communicable diseases (NCDs) were the primary contributor to ARDs, and there were significant variations in ARD burdens across countries. Of the 195 countries, Switzerland had the lowest ARD burden, while China ranked 75th and Vanuatu had the highest. The researchers found that the age-standardised ARD burden dropped globally between 1990 and 2017 due to lower disease severity and case fatality rates. Hu et al. assessed China's ARD burden based on the GBD 2017 study estimates at the national level. The researchers consistently found that NCDs are the primary contributor to ARDs in China. The age-standardised ARD burdens also decreased between 1990 and 2017, and the magnitude of ARD decrease was larger among women than men.[22] These findings emphasise the inadequacy of using chronological age alone to inform resource planning and policy design. They confirm the importance of considering health status and disease severity within the context of ageing and the need for concerted efforts to address regional disparities, especially for regions or countries with significant development inequalities.[21,22]

Many previous studies have assessed whether and how health resource allocation can impact health outcomes.[23–29] Health workforce density and total health expenditures per capita have been widely used as key proxies for health resources. A large body of high-quality evidence supports the view that a higher health workforce density can help improve health outcomes such as life expectancy, infant mortality rate, under-five mortality rate, and maternal mortality rate.[23,24] Evidence further shows heterogeneity among different types of the health workforce in terms of how they impact health outcomes.[23,24] The impact of health expenditures on health outcomes is mixed.[26–29] For example, evidence shows that an increase in health expenditures per capita can positively impact health outcomes, including life expectancy, under-five mortality, and maternal mortality in Sub-Saharan African countries.[29] However, a similar study did not find supporting evidence in Organisation for Economic Co-operation and Development (OECD) countries.[26] Another study found a significant association between higher health expenditures per capita and lower infant mortality in 15 European Union (EU) countries, but only marginal increases in life expectancy.[28]

For peer review only - http://bmjopen.bmj.com/site/about/guidelines.xhtml

China is a large, rapidly ageing country with uneven economic and health development across provinces. In 2020, 264 million of China's inhabitants (18.7%) were over 60 years old, ranking first globally by this metric, and this number is projected to double by 2050.[1,30] Generally, in China, the economy is most developed in eastern provinces, followed by the central and western provinces. For example, in 2020, the gross domestic product (GDP) per capita in Shanghai, a municipality located on China's east coast, was 4.3 times higher than that of Gansu, a province in western China.[30] Regional disparities in health development are similar in scale; provinces in the eastern region generally perform best, followed by the central and western provinces.[31] For example, among males in 2015, the average healthy life expectancy was estimated to be around 78 years in Beijing, Tianjin, and Shanghai, compared to 69 years in Qinghai, Tibet, and Yunnan, located in western China.[32] China is estimated to attain 12 of the 28 health-related Sustainable Development Goals (SDGs) indicators by 2030, and the country's eastern provinces are estimated to achieve a higher number.[33] Therefore, understanding the disparities in the disease burden of ageing across provinces and the impact of health resource allocation on the burden is crucial for China's central and provincial governments to plan for an ageing society. Nevertheless, no study has yet been conducted for this purpose. To fill the evidence gap, this study aims to: 1) measure the burden of ARDs in China at the subnational level, examining disparities across provinces and by sex, 2) assess changes in ARD burden from 1990–2016, and 3) explore how health resource allocation impacts the ARD burden. We also examined the difference by disease group whenever possible.

Methods

Overview

This study used estimates from the GBD study to calculate and analyse the ARD burden in China. The GBD team have updated their estimates annually since 2015 (except for 2018), with improvements on disease causes, risk factors, data input sources, and modelling strategies to obtain updated and robust estimates. The most recent publicly released estimates were GBD 2019.[34,35] We used GBD 2019 study estimates to select ARDs and calculate the total national burden considering its timeliness. We analysed the ARD burden at the subnational level based on estimates of the GBD 2016 study, as they were the most recent subnational estimates of China our team could access. However, we did not find significant changes in the causes and estimates that could impact the robustness of the study findings after careful comparison. The detailed methodology of the GBD 2016 and 2019 studies has been published elsewhere.[35,36] The GBD study classified the causes hierarchies into four levels, each with increased specificity.[37] Level One consists of three general causes: communicable diseases, NCDs, and injuries. Levels Two, Three, and Four further divide these causes into subgroups.[37] The data related to provincial health expenditures, human resources for health, and economic and demographic indicators were obtained from China Statistical and Health Statistical Yearbooks, providing data from 2010 to 2016 for regression analysis.

The geographical units of analysis are provincial-level jurisdictions in mainland China, including 22 provinces, five autonomous regions, and four municipalities (hereafter referred to as 31 provinces).[30] Our study complies with the guidelines for Accurate and Transparent Health Estimates Reporting (known as GATHER) statement, and we provided the GATHER checklist in the appendix.

ARD selection and burden measurement

Considering data availability, we followed the definition developed by Chang et al. to measure ARDs as diseases with incidence rates that increased quadratically with age among the adult population.[21] Our selection of diseases focused on GBD Level Three causes, and the detailed selection methods have been

For peer review only - http://bmjopen.bmj.com/site/about/guidelines.xhtml

published elsewhere.[21] To include a comprehensive list of diseases, we focused on the population aged 15 years and older.

The ARD burden was measured by calculating the sum of the DALYs of the identified ARDs in the entire population. DALYs are defined as the sum of the years of life lost due to premature mortality and the years lived with a disability due to a disease or health condition. We calculated the provincial ARD burden of non-communicable diseases (NCDs) to approximate the total ARD burden to analyse regional disparities, as previous studies showed NCDs contributed to over 80% of the ARD burden,[21,22] and we could only access the subnational estimates of NCDs. We reported the ARD burden rate, measured as DALYs per 100,000 population, to facilitate comparisons. To explore the burden composition by disease category, the ARD burden was further stratified and analysed at the second level of GBD causes within NCDs and by sex when appropriate. We also calculated the percentage decrease in the ARD burden to assess the historical change and clustered the results by disease category and region for comparative analysis (please see appendix Table S1 for the administrative divisions).

The ARD burden-adjusted age was defined as the equivalent age measured by the ARD burden. We estimated the ARD burden-adjusted age of each province to facilitate an intuitive assessment and comparison across provinces. We first identified the national ARD burden-adjusted age based on its age-specific burden rate. We selected the two five-year age groups with the closest burden rate as the national average and calculated the national average age by assuming a linear increase in the ARD burden within each five-year age group. Afterwards, the provincial ARD burden-adjusted age was calculated, assuming each province shared the same burden rate per age as the national average (appendix for calculation details). A younger ARD burden-adjusted age, therefore, implied a lower ARD burden.

To compare the historical change in the ARD burden, we applied age standardisation to the calculation using the Institute for Health Metrics and Evaluation (IHME) standard population age structure when necessary (Appendix Table S2 for the standard population structure). The 95% uncertainty intervals (UI), generated by the GBD study team as the 25th and 975th ordered 1000 random draw values of the posterior distribution,[36,38] were also presented for the ARD burden estimates.

Analysing the impact of health resource allocation on the ARD burden

We explored the impact of health resource allocation on the ARD burden using a panel data analysis approach. All equations were estimated with a log-linear functional form to enable unit-free comparisons of coefficients. To understand the underlying reasons for burden shifts over time, we used the age-standardised ARD burden rate as the dependent variable. Health expenditures and workforce density were adopted as proxy measures for health resources. Total health expenditure per capita was the key independent variable used to measure health expenditures.[26,27] The key independent variables used to measure health workforce density were three separate sets of indicators: total health professional density, licensed doctor density, and licensed nurse density, all per 1,000 population. Health professionals included licensed doctors (clinical, dental, public health, and traditional Chinese medicine), licensed nurses, pharmacists, clinical laboratory technicians, and radiologists.[39] We chose three sets of indicators to measure health workforce density because 1) together, they accurately represent the distribution of China's health personnel resources; 2) they are widely used in published literature, and heterogeneity exists in terms of their impact on health outcomes;[23,24] 3) data stratified by province and by urban and rural areas are available for all three indicators. We ran three separate regression models to include the three health workforce density indicators.

For peer review only - http://bmjopen.bmj.com/site/about/guidelines.xhtml

We included GDP per capita, sex, and education as covariates in the regression models to account for the major socio-economic determinants of the population health burden. China has made remarkable achievements over the past decade in reducing the health disparities between urban and rural residents, primarily through improving maternal and child health and extending health insurance coverage, among others, for its rural residents.[40] However, there is still a noticeable urban-rural gap in health development, including access to quality health services, health workforce quantity and quality, and health outcomes.[41–43] Therefore, to account for the significant gaps in urban-rural development and health across China, we included the percentage of people residing in urban areas (to measure the process of urbanisation) and the ratio of urban-rural health workforce density in the model as covariates. The model also included time dummies (to control period effects), province fixed effects, and an error term (see appendix for model details). We further explored the correlation of key variables to assess whether multicollinearity could undermine the robustness of the estimates for specific variables in the regression model (appendix, Table S3, and S4). Standard errors were clustered at the provincial level. Province was the unit of analysis, and a fixed-effects estimator was used to remove the potential endogeneity from time-invariant omitted variables.

Since provincial-level data on health expenditures per capita and health workforce density were only broadly available from 2010 onwards, our panel dataset included data from 2010 to 2016. We performed log-linear interpolation to obtain annual estimates of the ARD rate from 2010 to 2016, as the GBD study only provides estimates in five-year intervals.[38] The health expenditures per capita in 2010 are missing for seven provinces: Shanghai, Hainan, Sichuan, Tibet, Shaanxi, Qinghai, and Ningxia. Only data on Tibet is missing in 2011. Therefore, after testing the robustness of the linear increase assumption, we imputed the missing data, assuming a linear increase in per capita health expenditures between 2010 and 2016. All analyses were performed in Stata 16·0 (Stata Corp LLP, College Station, TX, USA).

Patient and public involvement statement

Patients or the public were not involved in the design, conduct, reporting, or dissemination of this research.

Results

A total of 58 causes out of 169 causes (34·3%) at level three were selected as ARDs in China (Table 1). Nine of the 58 causes were infectious diseases (15·5%), 47 were NCDs (81·0%), and two were injuries (3·5%). In 2016, the total burden of ARDs in China was 15703.7 DALYs (95% UI: 12628.5, 18406.2) per 100,000 population. NCDs accounted for 91·9% of the total burden. Among the NCDs, cardiovascular diseases (CVDs), neoplasms, chronic respiratory diseases (CRDs), sense organ diseases, and neurological disorders were the five leading contributors to the ARD burden.

Table 1. Summary of age-related diseases in China by category

GBD Causes at Level 1	GBD Causes at Level 2	GBD Causes at Level 3
**Infectious diseases**	Respiratory infections and Tuberculosis	Tuberculosis, diarrheal diseases, lower respiratory diseases
	Other infectious diseases	Meningitis, encephalitis, tetanus, varicella, and herpes zoster
	Neglected tropical diseases and malaria	Cysticercosis, trachoma

For peer review only - http://bmjopen.bmj.com/site/about/guidelines.xhtml

**NCDs**	Neoplasms	Oesophageal cancer, stomach cancer, liver cancer, tracheal, bronchus, and lung cancer, prostate cancer, colon and rectum cancer, lip and oral cavity cancer, other pharynx cancer, gallbladder, and biliary tract cancer, pancreatic cancer, malignant skin melanoma, non-melanoma skin cancer, bladder cancer, brain and central nervous system cancer, mesothelioma, Hodgkin lymphoma, non-Hodgkin lymphoma, multiple myeloma, leukaemia, other malignant neoplasms
	Cardiovascular Diseases	Ischemic heart disease, stroke, hypertensive heart disease, cardiomyopathy, myocarditis, atrial fibrillation and flutter, peripheral artery disease, endocarditis
	Chronic respiratory diseases	Chronic obstructive pulmonary disease, asthma, and interstitial lung disease
	Digestive Diseases	Paralytic ileus and intestinal obstruct, vascular intestinal disorders, pancreatitis
	Neurological disorders	Alzheimer's disease and other dementias, Parkinson's disease, idiopathic epilepsy, other neurological disorders
	Diabetes and kidney diseases	Acute glomerulonephritis, chronic kidney disease
	Skin and subcutaneous diseases	Fungal skin diseases, pruritus, decubitus ulcer, and other skin and subcutaneous diseases
	Sense organ diseases	Age-related and other hearing loss, other sense [illegible]
**Injuries**	Unintentional injuries	Falls, drowning

Data source: GBD 2019

Significant regional and sex disparities in ARD burdens existed in China. Generally, the eastern provinces had the lowest crude burden rates, while the northeastern and several western provinces bore the highest ones (Figure 1). After standardising the age structure, we noted tiered gaps in the age-standardised ARD burden among the eastern, central, and western provinces. Shanghai, Beijing, Zhejiang, and Jiangsu had the lowest burdens, while the burden of Tibet, Qinghai, Guizhou, and Xinjiang were the highest. There were salient disparities between the sexes, with age-standardised ARD burden rates being 61.2% higher among males than females on average. This disparity was most apparent in neoplasms, where the burden rate was 1·5 times higher among males. We observed an inversion of this trend only among neurological disorders, where the burden rate was 10.9% higher among females.

In 2016, China's crude ARD burden-adjusted age was 50·38 years (95% UI: 49.91. 50.91), following the same regional disparity pattern as measured by DALYs (Figure 2). Beijing, Guangdong, Shanghai, Zhejiang, and Fujian, all located in eastern China, were the youngest, with a crude ARD burden-adjusted age below 40 years. In comparison, Sichuan, Heilongjiang, Chongqing, Jilin, and Liaoning were the oldest, with a crude age close to or above 60 years. However, after the age structure across provinces was standardised, there were notable changes in the ARD burden-adjusted ages among several western provinces, especially for Tibet, Qinghai, Guizhou, and Xinjiang. These four regions were the oldest by the standardised age , at

For peer review only - http://bmjopen.bmj.com/site/about/guidelines.xhtml

72.70 (95% UI:60.53, 85.55), 66.82 (95% UI:59.36, 74.03), 60.53 (95% UI: 53.76, 63.56) and 60.06 (95% UI: 53.00, 66.88) years respectively. Shanghai, Beijing, and Zhejiang still had the youngest age-standardised ARD burden-adjusted ages, all below 40 years.

Between 1990 and 2016, China's age-standardised ARD burden decreased despite the increasing life expectancy, with a national average decline of 34·6% compared to the 1990 burden. We found regional disparities in the magnitude of this decline. The largest reduction was seen in the eastern provinces, followed by the central and western provinces, with the average ARD burdens declining by 35·2%, 33·9%, and 32·7%, respectively (Figure 3). Breaking this down by province, Fujian experienced the most significant decline in the age-standardised ARD burden (42·2%), followed by Zhejiang (42·0%) and Beijing (40·7%). Guangxi, Qinghai, and Guizhou experienced the smallest ARD burden decline in the same period, at 27·8%, 27·6%, and 27·5%, respectively. The ARD burden declined more sharply in females (41·4%) than in males (29·8%). Between 1990 and 2016, the ARD burden had decreased for CVDs, neoplasms, and CRDs, stayed almost unchanged for sense organ diseases and increased for neurological disorders (Figure 4). CRDs experienced the sharpest decline (67.9%), followed by neoplasms (30.5%) and CVDs (26·8%). However, the burden of neurological disorders increased by 5% during the same period.

China's health investment grew between 2010 and 2016. The country's total health expenditures per capita increased from 233·9 USD (exchange rate, 1 USD≈6·37 CNY) in 2010 to 526·2 USD in 2016. The total health professional density (the number of total health professionals per 1,000 people) increased by 38·6% from 4·4 in 2010 to 6·1 in 2016. In the same period, the licensed doctor density increased by 27·8%, while the licensed nurse density increased by 66·7% (Appendix for Table S5).[39,44] We report the regression results in Table 2. All coefficients of the health resource variables had the expected sign. The model results showed that neither higher health expenditures per capita nor higher health workforce density could significantly lead to a lower ARD burden, ceteris paribus. However, we found that the existing urban-rural gap in health workforce density was positively associated with the ARD burden, significant at the 10% level for all three indicators. A 100% increase in the urban-rural ratio in total health professional density, licensed doctor density, and licensed nurse density led to 2·37% (95% CI: -0·41, 5·15), 2·10% (95% CI: -0·27, 4·46), and 2.02% (95% CI: -0·34, 4·39) increases in the ARD burden respectively, ceteris paribus.

For peer review only - http://bmjopen.bmj.com/site/about/guidelines.xhtml

Table 2. Regression model results: Assessing the impact of health resources on age-standardised age-related diseases burden in mainland China, 2010–2016

Independent Variables	Model 1 (Total health professional density)		Model 2 (Licensed doctor density)		Model 3 (Licensed nurse density)	
	Coefficient (β)	95% CI	Coefficient (β)	95% CI	Coefficient (β)	95% CI
Total health expenditures per capita	-2.29	(-6.46, 1.88)	-2.02	(-6.18, 2.14)	-2.32	(-6.56, 1.91)
Health professional density	-1.34	(-3.48, 0.79)	..	..	..	..
Health professional density: urban-rural ratio	2.37*	(-0.41, 5.15)	..	..	..	..
Licensed doctor density	..	..	-0.93	(-3.30, 1.44)	..	..
Licensed doctor density: urban-rural ratio	..	..	2.10*	(-0.27, 4.46)	..	..
Licensed nurse density	..	..	..	..	-1.33	(-3.61, 0.94)
Licensed nurse density: urban-rural ratio	..	..	..	..	2.02*	(-0.34, 4.39)
GDP per capita	-0.16	(-5.09, 4.77)	-1.00	(-6.34, 4.33)	-0.34	(-5.57, 4.89)
Population living in urban areas (%)	1.82	(-15.60, 19.24)	1.83	(-15.13, 18.80)	3.03	(-14.95, 21.01)
Female (%)	-0.60	(-2.50, 1.30)	-0.87	(-2.66, 0.92)	-0.83	(-2.80, 1.14)
≥ junior middle school education (%)	3.25	(-1.66, 8.16)	2.30	(-2.25, 6.84)	3.03	(-1.72, 7.79)
adj. R^2	0.93	..	0.93	..	0.93	..

Data sources: GBD 2016, Statistical Yearbook of China (2011–2017), and the Health Statistical Yearbook of China (2011–2017)

Notes:
All dependent and independent variables were transformed into natural logarithms for regressions except for the time dummies. The independent variables of interest and covariates were rescaled by dividing by 100. The coefficients can be interpreted as follows: every 100% increase in X can lead to a β% increase in Y.
Model 1 used total health professional density (per 1,000 population) as a proxy for human resources for health.
Model 2 used licensed doctor density as a proxy (per 1,000 population) for human resources for health.
Model 3 used licensed nurse density as a proxy (per 1,000 population) for human resources for health.
Significance level: *(p<0·10); **(p<0·05); ***(p<0·01)

For peer review only - http://bmjopen.bmj.com/site/about/guidelines.xhtml

Discussion

Healthy ageing has become a focal discussion topic in today's fast-ageing world, as good health in advanced age can provide continued opportunities for social and personal development.[2] The World Health Organisation officially proposed the concept of healthy ageing in 2015 and published the Global Strategy and Action Plan on Ageing and Health (2016–2020) in 2016. [2,45] The Decade of Healthy Ageing: Plan of Action (2020–2030) was issued in 2020 and asked for a whole-of-government and whole-of-society response to healthy ageing.[46] In response to this international call and the needs of the world's largest ageing population, China has actively worked to address the shifts catalysed by an ageing society and promote healthy ageing.[47–49] We conducted this study to generate high-quality evidence to help China and similar countries be better equipped to face health challenges in ageing societies. To our knowledge, this is the first study that assesses the disease burden of ageing and its longitudinal changes in 31 provinces of mainland China from 1990 to 2016. The analysis explored disparities in ARD burdens across regions, sexes, and disease categories and used panel regression models to examine the impact of health resource indicators on the ARD burden.

Our findings underscore several key messages on the disease burden of ageing in China. First, NCDs account for over 90% of the country's total ARD burden. CVDs, neoplasms, CRDs, sense organ diseases, and neurological disorders were the top five contributors to the ARD burden. Second, there are significant regional disparities in ARD burdens. The results show tiered differences in the ARD burden, with the lowest rates in the eastern provinces, followed by the central provinces, and the highest rates in the northeastern and western provinces. Notably, the crude ARD burden rate in Sichuan was 2.1 times that of Beijing in 2016, equal to a 34·14-year age gap measured by the ARD burden-adjusted age defined in this study. Third, several western provinces will face daunting ARD-induced challenges as their populations begin to age. After age structure standardisation, Tibet, Qinghai, Guizhou, and Xinjiang had the four highest ARD burdens. However, in 2016, less than 10% of these provinces' populations were aged 65 years and older, compared to a national average of 10.8%.[30] Fourth, males are disproportionally affected by ARDs except for neurological disorders. Fifth, the overall age-standardised ARD burden has decreased since 1990, though the most significant decline was observed in the eastern provinces, followed by the central and western provinces. CRDs experienced the most significant decline in age-standardised burden by disease category, followed by neoplasms and CVDs. The burden rate of sense organ diseases has stayed almost unchanged since 1990. The burden of neurological disorders has increased since 1990, albeit by a small magnitude. Lastly, our results suggest that greater investment in health to reduce the urban-rural gap in the health workforce could help lower China's ARD burden.

Our study findings are consistent with previous studies that assessed the ARD burden globally and in China.[21,22] There is overall support for the idea that NCDs are the chief contributor to ARDs. As has been the case across the globe, the ARD burden has continuously decreased since 1990, declining most sharply in developed countries and regions. Disparities by sex are also similar; males are more negatively affected by the ARD burden and have experienced a lower historical decline. The magnitude of the burden measured in this study is slightly different from those in other studies due to differences across GBD datasets and the populations and diseases included. Our findings are novel in that we were able to measure the ARD burden and disparities across provinces in China and examine the impact of health resource indicators on the burden. Nevertheless, the regional disparities of age-standardised ARD burdens align with their health development status and are similar to the disparities in estimated healthy life expectancy.[50–33] The rank of NCD burdens among the ARDs is consistent with that of the disease burden among older people in China.[34] Interestingly, we found that increasing health expenditures or workforce density does not significantly decrease the ARD burden; instead, the key is to reduce the urban-rural gap in the health workforce density. Previous evidence has indicated that increasing health workforce density

For peer review only - http://bmjopen.bmj.com/site/about/guidelines.xhtml

can improve health outcomes.[23,24] However, these studies focus on measuring health outcomes such as maternal mortality, infant and under-five mortality as opposed to ageing-related outcomes. In addition, these studies did not control for geographic resource differences between rural and urban areas and were conducted in countries where resources were scarce. Thus, the context was quite different from that of the present study.

The Chinese State Council published *The Opinions on Strengthening Aged Care Work in the New Era* in November 2021, outlining eight important action domains for addressing ageing. This document serves as a comprehensive, strategic plan to achieve healthy and active ageing in China.[49] The findings from the current study provide timely input into policy development for the governments of China to promote healthy ageing by reducing the disease burden of ageing. First, to reiterate the findings from previous studies, chronological age alone does not provide a strong enough basis for appropriate ageing resource planning or policymaking.[21,22] In China, provinces or municipalities with older populations, such as Shanghai, Beijing, and Zhejiang, have much lower ARD burdens than some younger provinces, such as Tibet and Xinjiang. This phenomenon calls for careful consideration of underlying health burdens and potential threats to resource planning and policy design to address future ageing. Second, it is crucial to continue and strengthen NCD prevention and control efforts, especially for CVDs, neoplasms, CRDs, sense organ diseases, and neurological disorders. To prevent the continued increase in the ARD burden from neurological disorders, it is imperative to allocate additional health resources for the prevention, treatment, and management of conditions such as Alzheimer's, dementia, Parkinson's, and idiopathic epilepsy. Sense organ diseases cannot be ignored, especially those leading to blindness and vision loss, which can significantly damage the quality of life for the elderly. Third, the central government should continue to provide all-around support to the regions that face the greatest threat of high ARD burdens due to future ageing, especially the western provinces of Tibet, Qinghai, Guizhou, and Xinjiang, to reduce regional disparities. Notably, that support should entail the establishment of a strong health workforce that can serve the local people. Fourth, aligned with the Rural Revitalization Strategic Plan issued by the State Council (2018–2022) in 2018,[51] the central and local governments should work collaboratively to strengthen the rural health system, particularly by increasing the health workforce density to reduce the urban-rural gap.

Our team have carefully compared the difference between estimates from the GBD 2016 and 2019 study to ensure the robustness of the findings from the current study. Methodologically, the GBD 2019 study generally takes a similar approach as used in the GBD 2017 study, which has enriched data input sources, modelling parameters, disease categories, and upgraded calibration methods to get more accurate results compared to the GBD 2016 study.[35,36] Our comparison results show that the crude ARD burden rate in 2016 based on estimates from GBD 2019 is slightly higher than estimates from GBD 2016, though the difference is within 8% and as low as 2% for neoplasms. Regarding the diseases included, we did not find significant changes at level three that would impact the results of the ARD burden. The findings are consistent with the comparison conducted by a previous study on the estimates generated by the GBD 2016 and 2017 study for the subnational level of China.[33] Therefore, subnational estimates of GBD 2016 can well represent the regional disparities of the ARD burden in China.

Our study has several limitations. First, the subnational analysis was based on the GBD 2016 study estimates, which were not updated to 2019, as we do not have access to the GBD 2019 estimates at the subnational level of China. However, this was the best data source we could access, and as stated above, the results from the GBD 2016 study are robust to show regional disparities after careful comparison. Second, our provincial analysis of the ARD burden focused on NCDs due to data availability, and we did not explore the burden of specific diseases. However, as NCDs accounted for over 90% of the ARD burden

For peer review only - http://bmjopen.bmj.com/site/about/guidelines.xhtml

in China, these results can accurately represent existing regional disparities. Third, the study findings tend to underestimate the ARD burden, as GBD study estimates fail to model the interactions between diseases, ignoring the burden caused by multimorbidity. Forth, though the definition used in the current study to measure the burden of age-related diseases is novel and easy to operationalise, it is only one of the many ways to measure it.[21,52–54] Future studies can gauge the pros and cons of different measurements and choose the most appropriate one for analysis. Fifth, we had to rely on interpolation to obtain annual estimates of burden rates from 2010 to 2016 for panel data analysis. However, the GBD research team used the same methods in their analysis.[38] In addition, the indicators for health resources are limited to total health expenditures and health workforce density, mainly due to data availability. Other important indicators, such as the efficiency of health funds utilisation and the quality of the health workforce, could be explored in future research when data become available.

Conclusion

In conclusion, our study provides valuable and original insights into the disease burden of ageing in China and the existing regional disparities. Our results consistently show that an older demographic structure does not necessarily mean a heavier disease burden. Therefore, chronological age alone does not provide a strong enough basis for appropriate ageing resource planning or policymaking. Continuing to invest in population health by reducing the urban-rural gap in health workforce density would help reduce the disease burden of ageing in China. In so doing, the Government of China needs to develop an effective policy and mechanism to attract more health professionals to work in rural and less developed areas. The governments of China, or other countries with similar demographic and disease profiles and development contexts, need to take urgent action to decrease the disease burden of ageing to create healthy ageing societies.

Contributors

SC designed the study under the supervision of KH, BL, and HB. SC extracted the data, conducted the statistical analysis in Stata, developed the tables and figures, and drafted the manuscript. SC and YS verified the data in the study. All co-authors (SC, YS, KH, BL, HB, XD, CW, and ST) contributed to commenting, editing, and approval of the final manuscript. All accept responsibility for submission to the journal for publication.

Declaration of interests

All co-authors declare no conflicts of interest for this study.

Acknowledgement

We thank the Institute for Health Metrics and Evaluation (IHME) for making this study possible by sharing the GBD 2016 study estimates from China at the subnational level. We also appreciate the technical instructions from Dr. Cheng Wan (UNSW) and Mr. Lei Guo (Duke Global Health Institute) on drawing the maps.

Funding statement

The study received funding support from the Bill & Melinda Gates Foundation (OPP1148464), the UNSW, the ARC Centre of Excellence in Population Ageing Research (CEPAR), SHARP Fund UNSW (SHARP001). The funders had no role in study design, data collection, data analysis, data interpretation, or manuscript writing.

Data availability statement

For peer review only

For peer review only - http://bmjopen.bmj.com/site/about/guidelines.xhtml

China's subnational-level data from GBD 2016 were obtained from the IHME under a private agreement. Researchers can apply for data access at https://www.healthdata.org/about/contact-us if interested in using the data. Other inputs data were obtained from open resources, including China Statistical Yearbook (http://www.stats.gov.cn/tjsj/ndsj/) and China Health Statistical Yearbook (http://www.nhc.gov.cn/wjw/tjnj/list.shtml). Researchers can access and download the data from these websites.

Ethics statement

Ethics approval

Not applicable.

Patient consent for publication

Not applicable.

For peer review only

For peer review only - http://bmjopen.bmj.com/site/about/guidelines.xhtml

References

1 UN Department of Economic and Social Affairs, Population Division. World population ageing, 2019 highlights. 2020.

2 Beard JR, Officer A, de Carvalho IA, *et al.* The World report on ageing and health: a policy framework for healthy ageing. *The Lancet* 2016; **387**: 2145–54.

3 Manton KG, Gu X, Lamb VL. Change in chronic disability from 1982 to 2004/2005 as measured by long-term changes in function and health in the U.S. elderly population. *Proc Natl Acad Sci* 2006; **103**: 18374–9.

4 Chatterji S, Byles J, Cutler D, Seeman T, Verdes E. Health, functioning, and disability in older adults—present status and future implications. *The Lancet* 2015; **385**: 563–75.

5 Seeman TE, Merkin SS, Crimmins EM, Karlamangla AS. Disability Trends Among Older Americans: National Health and Nutrition Examination Surveys, 1988–1994 and 1999–2004. *Am J Public Health* 2010; **100**: 100–7.

6 Crimmins EM, Beltran-Sanchez H. Mortality and Morbidity Trends: Is There Compression of Morbidity? *J Gerontol B Psychol Sci Soc Sci* 2011; **66B**: 75–86.

7 Kyu HH, Abate D, Abate KH, *et al.* Global, regional, and national disability-adjusted life-years (DALYs) for 359 diseases and injuries and healthy life expectancy (HALE) for 195 countries and territories, 1990–2017: a systematic analysis for the Global Burden of Disease Study 2017. *The Lancet* 2018; **392**: 1859–922.

8 Lafortune G, Balestat G. Trends in Severe Disability Among Elderly People: Assessing the Evidence in 12 OECD Countries and the Future Implications. Paris, 2007 DOI:10.1787/217072070078.

9 Jagger C, Gillies C, Moscone F, *et al.* Inequalities in healthy life years in the 25 countries of the European Union in 2005: a cross-national meta-regression analysis. 2008; **372**: 8.

10 UN Department of Economic and Social Affairs Population Division. World Population Ageing: 1950-2050. New York, 2017 http://globalag.igc.org/ruralaging/world/ageingo.htm (accessed Aug 12, 2021).

11 Fried LP, Tangen CM, Walston J, *et al.* Frailty in Older Adults: Evidence for a Phenotype. *J Gerontol A Biol Sci Med Sci* 2001; **56**: M146–57.

12 Searle SD, Mitnitski A, Gahbauer EA, Gill TM, Rockwood K. A standard procedure for creating a frailty index. *BMC Geriatr* 2008; **8**: 24.

13 Edemekong PF, Bomgaars DL, Sukumaran S, Levy SB. Activities of Daily Living. In: StatPearls. Treasure Island (FL): StatPearls Publishing, 2021. http://www.ncbi.nlm.nih.gov/books/NBK470404/ (accessed Aug 11, 2021).

14 Kempen GIJM, Suurmeijer TPBM. The Development of a Hierarchical Polychotomous ADL-IADL Scale for Noninstitutionalized Elders. *The Gerontologist* 1990; **30**: 497–502.

For peer review only - http://bmjopen.bmj.com/site/about/guidelines.xhtml

15 Mahoney F, Barthel D. FUNCTIONAL EVALUATION: THE BARTHEL INDEX - PubMed. *Md State Med J* 1965; **14**: 61–5.

16 Skirbekk V, Loichinger E, Weber D. Variation in cognitive functioning as a refined approach to comparing aging across countries. *Proc Natl Acad Sci* 2012; **109**: 770–4.

17 Beard JR, Jotheeswaran AT, Cesari M, Araujo de Carvalho I. The structure and predictive value of intrinsic capacity in a longitudinal study of ageing. *BMJ Open* 2019; **9**: e026119.

18 Beard J, Si Y, Chenoweth L, Hanewald K. Intrinsic Capacity: Validation of a New WHO Concept for Healthy Ageing in a Longitudinal Chinese Study. *J Gerontol A Biol Sci Med Sci* 2021; published online Aug 3. DOI:10.1093/gerona/glab226.

19 Brooks R. EuroQol: the current state of play. *Health Policy Amst Neth* 1996; **37**. DOI:10.1016/0168-8510(96)00822-6.

20 EuroQol Group. EuroQol - a new facility for the measurement of health-related quality of life. *Health Policy* 1990; **16**: 199–208.

21 Chang AY, Skirbekk VF, Tyrovolas S, Kassebaum NJ, Dieleman JL. Measuring population ageing: an analysis of the Global Burden of Disease Study 2017. *Lancet Public Health* 2019; **4**: e159–67.

22 Hu D, Yan W, Zhu J, Zhu Y, Chen J. Age-Related Disease Burden in China, 1997–2017: Findings From the Global Burden of Disease Study. *Front Public Health* 2021; **0**. DOI:10.3389/fpubh.2021.638704.

23 Anand S, Bärnighausen T. Human resources and health outcomes: cross-country econometric study. *The Lancet* 2004; **364**: 1603–9.

24 Speybroeck N, KINFU Y, Dal Poz M, Evans D. Reassessing the Relationship Between Human Resources for Health, Intervention Coverage and Health Outcomes. *Reassessing Relatsh Hum Resour Health Interv Cover Health Outcomes* 2006; : 1–14.

25 Nguyen MP, Mirzoev T, Le TM. Contribution of health workforce to health outcomes: empirical evidence from Vietnam. *Hum Resour Health* 2016; **14**: 68.

26 Panopoulou E, Pantelidis T. Convergence in per capita health expenditures and health outcomes in the OECD countries. *Appl Econ* 2012; **44**: 3909–20.

27 Bokhari FAS, Gai Y, Gottret P. Government health expenditures and health outcomes. *Health Econ* 2007; **16**: 257–73.

28 Nixon J, Ulmann P. The relationship between health care expenditure and health outcomes. Evidence and caveats for a causal link. *Eur J Health Econ HEPAC Health Econ Prev Care* 2006; **7**: 7–18.

29 Nketiah-Amponsah E. The Impact of Health Expenditures on Health Outcomes in Sub-Saharan Africa. *J Dev Soc* 2019; **35**: 134–52.

[dataset] 30 National Bureau of Statistics, China. Data from: National data, National Bureau of Statistics, China. 2020. https://data.stats.gov.cn/easyquery.htm?cn=E0103. Aug 12, 2021.

For peer review only - http://bmjopen.bmj.com/site/about/guidelines.xhtml

31 Zhou M, Wang H, Zeng X, *et al.* Mortality, morbidity, and risk factors in China and its provinces, 1990–2017: a systematic analysis for the Global Burden of Disease Study 2017. *The Lancet* 2019; **394**: 1145–58.

32 Li H, Hanewald K, Wu S. Healthy life expectancy in China: Modelling and implications for public and private insurance. *Ann Actuar Sci* 2021; **15**: 40–56.

33 Chen S, Guo L, Wang Z, *et al.* Current situation and progress toward the 2030 health-related Sustainable Development Goals in China: A systematic analysis. *PLOS Med* 2019; **16**: e1002975.

[dataset] 34 Global Burden of Disease Collaborative Network. Data from: Global Burden of Disease Study 2019 (GBD 2019) Results. 2020. http://ghdx.healthdata.org/gbd-results-tool. Aug 10, 2021.

35 Vos T, Lim SS, Abbafati C, *et al.* Global burden of 369 diseases and injuries in 204 countries and territories, 1990–2019: a systematic analysis for the Global Burden of Disease Study 2019. *The Lancet* 2020; **396**: 1204–22.

36 Vos T, Abajobir AA, Abate KH, *et al.* Global, regional, and national incidence, prevalence, and years lived with disability for 328 diseases and injuries for 195 countries, 1990–2016: a systematic analysis for the Global Burden of Disease Study 2016. *The Lancet* 2017; **390**: 1211–59.

37 IHME. Frequently Asked Questions. Inst. Health Metr. Eval. 2014; published online May 9. http://www.healthdata.org/gbd/faq (accessed Nov 15, 2021).

38 Vos T, Lim SS, Abbafati C, *et al.* Global burden of 369 diseases and injuries in 204 countries and territories, 1990–2019: a systematic analysis for the Global Burden of Disease Study 2019. *The Lancet* 2020; **396**: 1204–22.

[dataset] 39 Statistics and Information Center, National Health Commission, China. Data from: China Health Statistical Yearbook 2019. Beijing: Peking Union Medical College Press, 2019. http://www.nhc.gov.cn/mohwsbwstjxxzx/tjtjnj/202106/04bd2ba9592f4a70b78d80ea50bfe96e.shtml . Aug 12, 2021.

40 Meng Q, Mills A, Wang L, Han Q. What can we learn from China's health system reform? *BMJ* 2019; **365**: l2349.

41 Ma X, He Y, Xu J. Urban–rural disparity in prevalence of multimorbidity in China: a cross-sectional nationally representative study. *BMJ Open* 2020; **10**: e038404.

42 Yang D, Acharya Y, Liu X. Social health insurance consolidation and urban-rural inequality in utilisationand financial risk protection in China. *Soc Sci Med* 2022; **308**: 115200.

43 Health TLP. Public health in China: achievements and future challenges. *Lancet Public Health* 2018; **3**: e456.

[dataset] 44 Statistics and Information Center, National Health Commission, China. Data from: China Health Statistical Yearbook 2011. Beijing: Peking Union Medical College Press, 2011. http://www.nhc.gov.cn/mohwsbwstjxxzx/s7967/201301/f34860a7bdb64aad9029eb860a6cf4d2.shtml. Aug 12, 2021.

For peer review only

For peer review only - http://bmjopen.bmj.com/site/about/guidelines.xhtml

45 Global strategy and action plan on ageing and health. https://www.who.int/publications-detail-redirect/9789241513500 (accessed Dec 3, 2021).

46 World Health Organization. UN Decade of Healthy Ageing. https://www.who.int/initiatives/decade-of-healthy-ageing (accessed Dec 3, 2021).

47 Yang W, Chang S, Zhang W, *et al.* An Initial Analysis of the Effects of a Long-Term Care Insurance on Equity and Efficiency: A Case Study of Qingdao City in China. *Res Aging* 2021; **43**: 156–65.

48 Feng Z, Glinskaya E, Chen H, *et al.* Long-term care system for older adults in China: policy landscape, challenges, and future prospects. *The Lancet* 2020; **396**: 1362–72.

49 The State Council, China. The Opinions on Strengthening Aged Care Work in the New Era. 2021; published online Nov 24. http://www.gov.cn/zhengce/2021-11/24/content_5653181.htm (accessed Dec 3, 2021).

50 Li H, Hanewald K, Wu S. Estimating Healthy Life Expectancy: A Province-by-Province Study for China. Rochester, NY: Social Science Research Network, 2017 DOI:10.2139/ssrn.3027825.

51 The State Council, China. Rural Revitalization Strategic Plan (2018-2022). 2018; published online Sept 26. http://www.moa.gov.cn/ztzl/xczx/xczxzlgh/201811/t20181129_6163953.htm (accessed Dec 3, 2021).

52 Brody JA, Schneider EL. Diseases and disorders of aging: an hypothesis. *J Chronic Dis* 1986; **39**: 871–6.

53 Brody JA, Grant MD. Age- associated diseases and conditions: Implications for decreasing late life morbidity. *Aging Clin Exp Res* 2001; **13**: 64–7.

54 Kuan V, Fraser HC, Hingorani M, *et al.* Data-driven identification of ageing-related diseases from electronic health records. *Sci Rep* 2021; **11**: 2938.

For peer review only

For peer review only - http://bmjopen.bmj.com/site/about/guidelines.xhtml

Figure legend and footnote

Figure 1. The crude and age-standardised age-related diseases burden in 31 provinces of mainland China, 2016

Data source: GBD 2016
Note: The age-related diseases burden was standardized using the IHME standard population age structure (appendix)

Figure 2. The crude and age-standardised ARD burden-adjusted ages of 31 provinces in mainland China, 2016

Data source: GBD 2016
Note: The provinces were grouped into eastern, central and western provinces from top down.
The age-related diseases burden was standardized using the IHME standard population age structure (appendix)

Figure 3. Change of the age-standardised age-related diseases burden rates by administrative region in mainland China, 1990-2016

Data source: GBD 2016
Note: The age-related diseases burden was standardized using the IHME standard population age structure (appendix)

Figure 4. Change of the age-standardised age-related diseases burden rates of the five leading ARD contributors in mainland China, 1990-2016

Data source: GBD 2016
Note: The age-related diseases burden was standardized using the IHME standard population age structure (appendix)

For peer review only - http://bmjopen.bmj.com/site/about/guidelines.xhtml

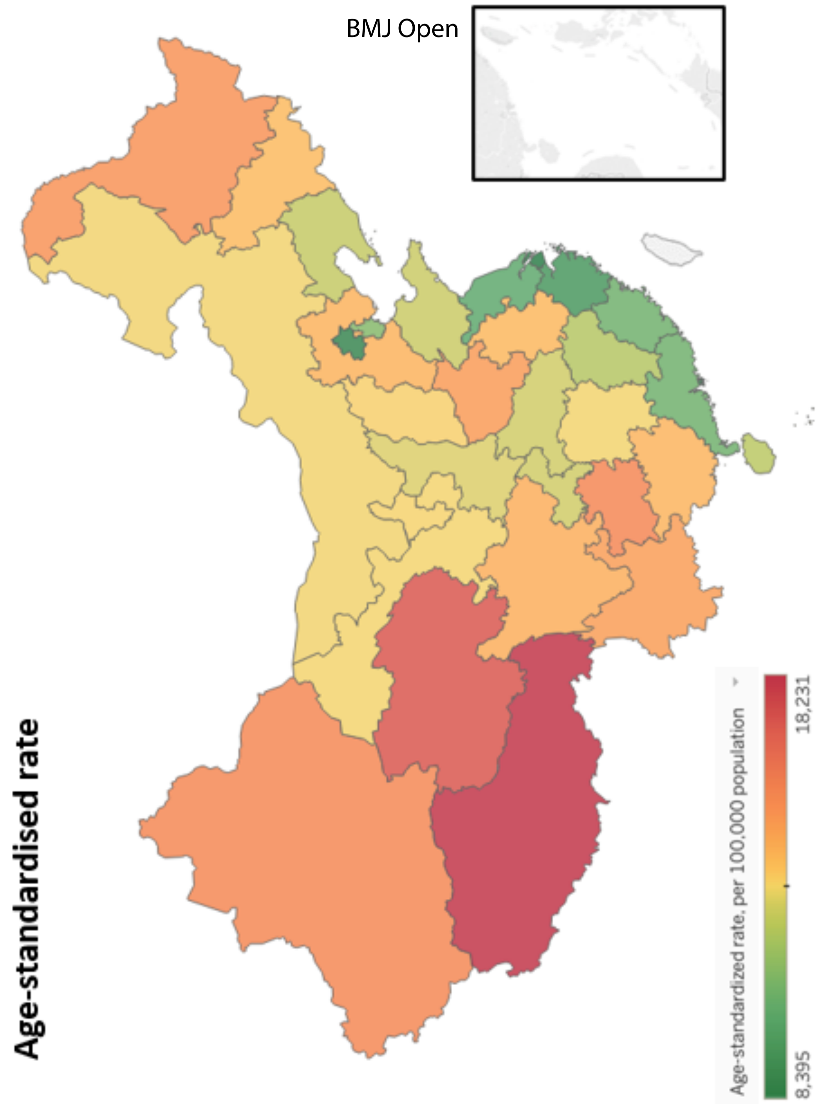
Age-standardised rate
Age-standardized rate, per 100,000 population
18,231
8,395

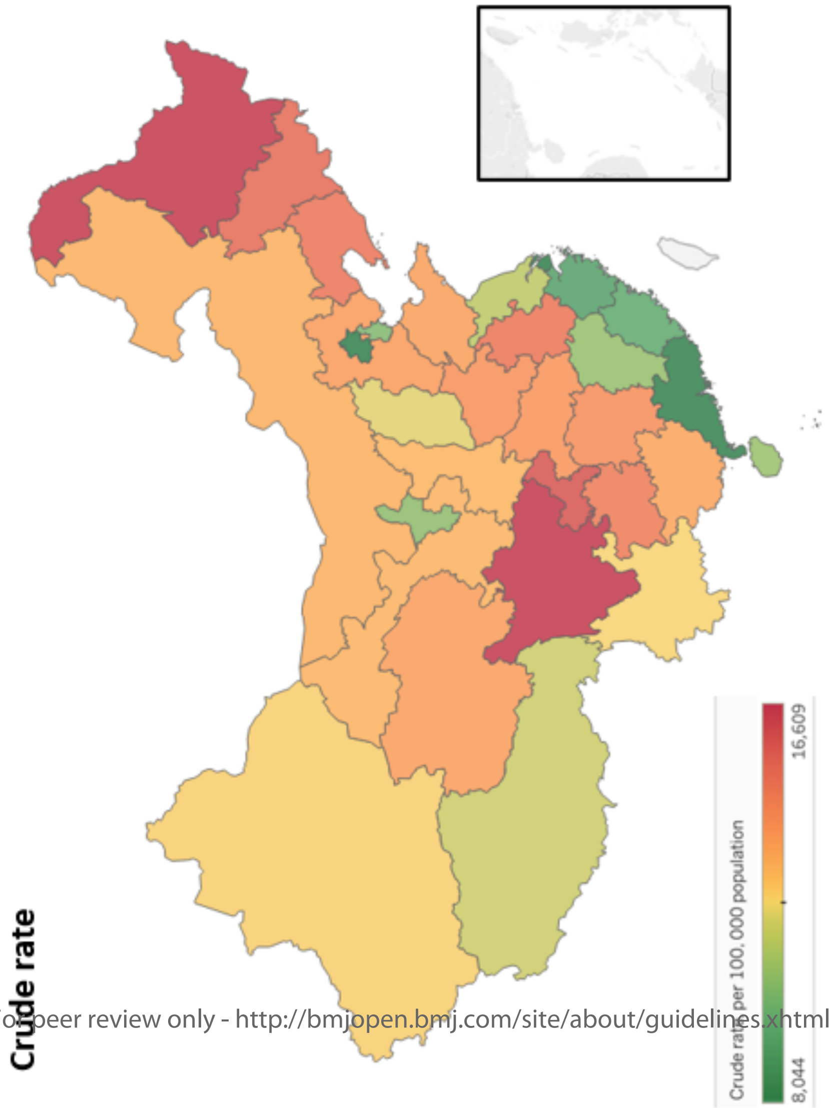
Crude rate
Crude rate per 100,000 population
16,609
8,044

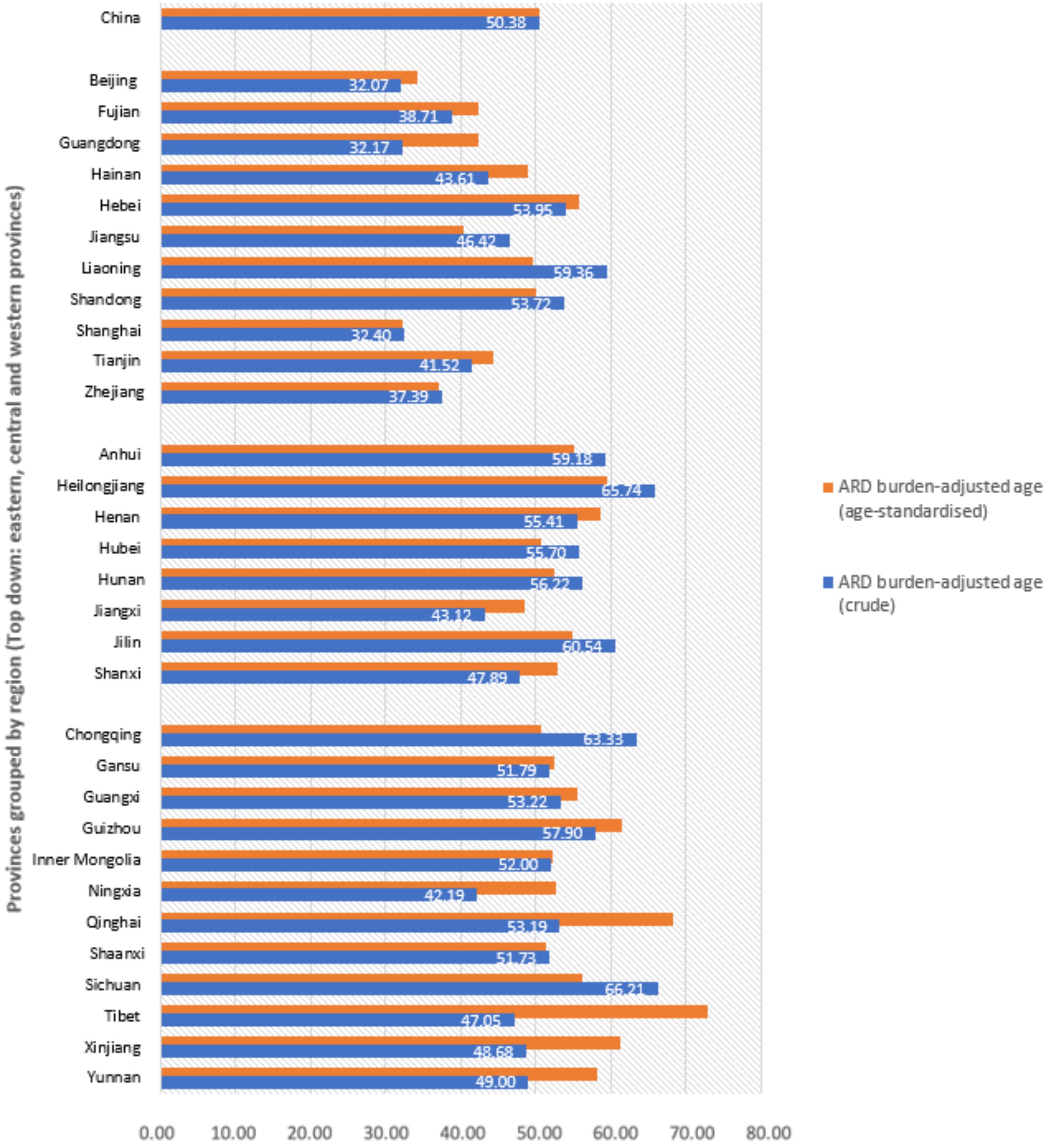
China
50.38
Beijing
32.07
Fujian
38.71
Guangdong
32.17
Hainan
43.61
Hebei
53.95
Jiangsu
46.42
Liaoning
59.36
Shandong
53.72
Shanghai
32.40
Tianjin
41.52
Zhejiang
37.39
Anhui
59.18
Heilongjiang
65.74
Henan
55.41
Hubei
55.70
Hunan
56.22
Jiangxi
43.12
Jilin
60.54
Shanxi
47.89
Chongqing
63.33
Gansu
51.79
Guangxi
53.22
Guizhou
57.90
Inner Mongolia
52.00
Ningxia
42.19
Qinghai
53.19
Shaanxi
51.73
Sichuan
66.21
Tibet
47.05
Xinjiang
48.68
Yunnan
49.00
Provinces grouped by region (Top down: eastern, central and western provinces)
0.00
10.00
20.00
30.00
40.00
50.00
60.00
70.00
80.00
ARD burden-adjusted age (age-standardised)
ARD burden-adjusted age (crude)

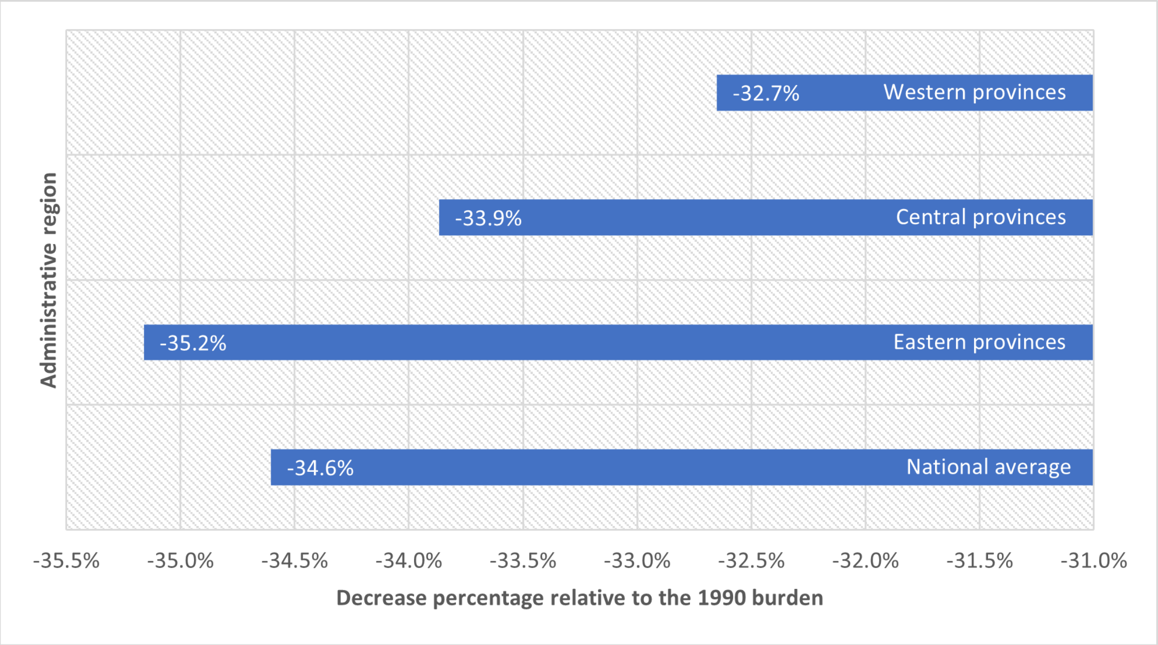
-32.7%
Western provinces
-33.9%
Central provinces
-35.2%
Eastern provinces
-34.6%
National average
Administrative region
-35.5%
-35.0%
-34.5%
-34.0%
-33.5%
-33.0%
-32.5%
-32.0%
-31.5%
-31.0%
Decrease percentage relative to the 1990 burden

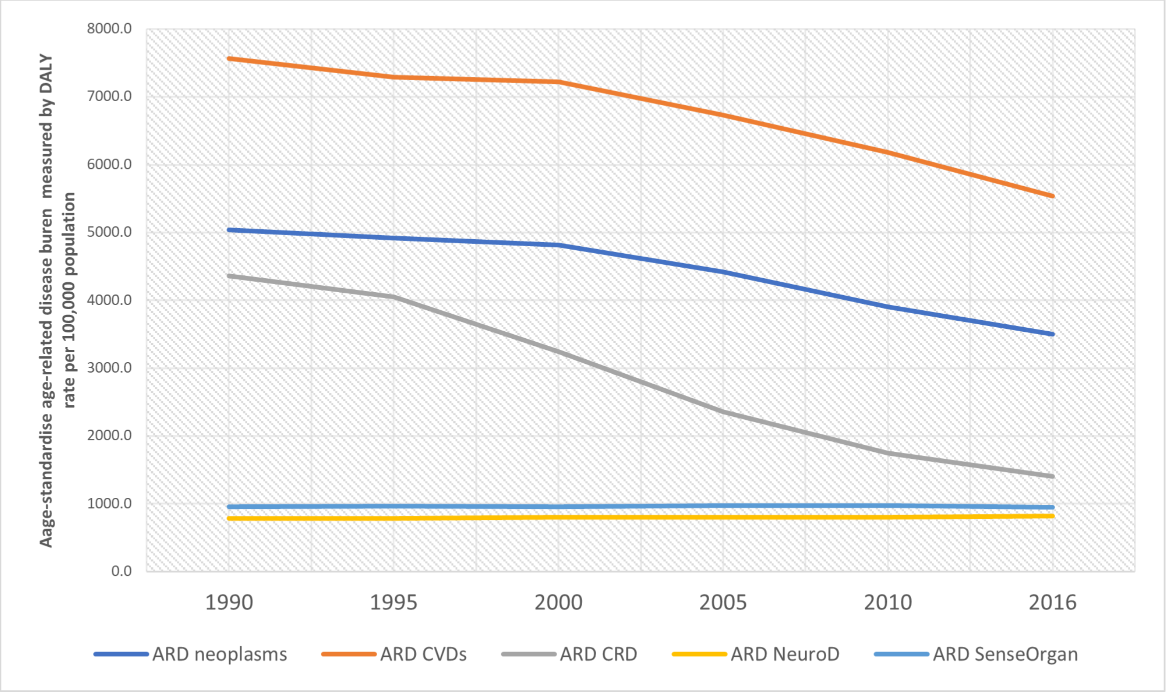

For peer review only

Appendix

Table S1. Administrative division of provinces in mainland China

Administrative division (No.)	Province
Eastern provinces (11)	Beijing, Fujian, Guangdong, Hainan, Hebei, Jiangsu, Liaoning, Shandong, Shanghai, Tianjin, Zhejiang
Central provinces (8)	Anhui, Heilongjiang, Henan, Hubei, Hunan, Jiangxi, Jilin, Shanxi
Western provinces (12)	Inner Mongolia, Guangxi, Chongqing, Gansu, Guizhou, Ningxia, Qinghai, Shaanxi, Sichuan, Tibet, Xinjiang, Yunnan

Table S2. IHME standard age weight

Age group	Population percentage
<5	7.2%
5–9	8.7%
10–14	8.4%
15–19	8.1%
20–24	7.8%
25–29	7.6%
30–34	7.2%
35–39	6.9%
40–44	6.4%
45–49	5.8%
50–54	5.3%
55–59	4.7%
60–64	4.1%
65–69	3.4%
70 +	8.6%

ARD burden-adjusted age calculation

First, we identified the ARD burden-adjusted age of China by comparing the national average burden rate with its burden rates by age group. We selected the two five-year age groups with the closest burden rate as the national average and calculated the national average age by assuming a linear increase in the ARD burden within each five-year age group. For example, the age-related disease burden of China was 12637.2 DALYs per 100,000 population in 2016, closest to burden rates for the age group of 45–49 (9316.3) and 50–54 (14163.7). Hence, the national age was calculated as: 45+12637.2/((9316.3+14163.7)/10)=50.38.

For peer review only - http://bmjopen.bmj.com/site/about/guidelines.xhtml

Second, we calculated the ARD burden-adjusted age of each province by dividing the provincial age-related disease burden rate by the unit share of the national average burden rate per age. The unit share of the national average burden rate per age was calculated by dividing the national average burden rate by the national equivalent age. For example, the age-related disease burden of Shanghai was 8,127·4 DALYs per 100,000 population. Hence, Shanghai's ARD burden-adjusted age was calculated as: 8,127·4/(12637·2/50.38)=32·40.

Regression model

$$Y_{it} = \alpha + \beta_0 healthexp_{it} + \beta_1 healthwf_{it} + \beta_2 X_{it} + \beta_3 D_t + \eta_i + \varepsilon_{it},$$

where Y_{it} is the age-standardised age-related disease burden of Province i in Year t, β_0 is the coefficient of interest, $healthexp_{it}$ is the total health expenditures per capita, and $healthwf_{it}$ is the total health professional density, licensed doctor density, or licensed nurse density (per 1,000 population) of the Province i in Year t (three separate models). X_{it} is the set of covariates controlled in the model, including GDP per capita, education, the proportion of females, the proportion of people living in urban areas, and the urban-rural health workforce density ratio. D_t represents the time dummies, η_i the province fixed effects, and ε_{it} is the error term. All variables are in log form except for the time dummies.

We also assessed the correlation between 1) the total health expenditures per capita and health workforce density and 2) the rural/urban ratio in health workforce density and the proportion of rural/urban population to check multicollinearity before and after controlling for covariates, including GDP per capita, sex and education. The results are presented below in Table S3 and Table S4.

For peer review only

Table S3. Regression model results: assessing correlation between health workforce density and total health expenditures per capita

Independent Variables	Model 1		Model 2		Model 3		Model 4		Model 5		Model 6	
	Coef (β)	P value	Coef (β)	P value	Coef (β)	P value	Coef (β)	P value	Coef (β)	P value	Coef (β)	P value
Health prof density	0.09	0.10	-0.04	0.34	··	··	··	··	··	··	··	··
Licensed doc density	··	··	··	··	0.11	0.06	0.01	0.89	··	··	··	··
Licensed nurse density	··	··	··	··	··	··	··	··	0.08	0.09	-0.02	0.51
GDP per capita	··	··	0.37	<0.01	··	··	0.33	<0.01	··	··	0.36	<0.01
Female (%)	··	··	0.08	0.32	··	··	0.08	0.35	··	··	0.08	0.33
≥ junior mid sch educ (%)	··	··	0.08	0.50	··	··	0.06	0.58	··	··	0.08	0.50
adj. R^2	0.96	··	0.94	··	0.96	··	0.94	··	0.96	··	0.94	··

Note:
Model 1: Total health professional density as independent variable, not controlling for covariates except for time dummies
Model 2: Total health professional density as independent variable, controlling for covariates except for time dummies
Model 3: Licensed doctor density as independent variable, not controlling for covariates except for time dummies
Model 4: Licensed doctor density as independent variable, controlling for covariates except for time dummies
Model 5: Licensed nurse density as independent variable, not controlling for covariates except for time dummies
Model 6: Licensed nurse density as independent variable, controlling for covariates except for time dummies

Table S4. Regression model results: assessing correlation between urban residency and urban-rural ratio in health workforce density

Independent Variables	Model 1		Model 2	
	Coef (β)	P value	Coef (β)	P value
Population living in urban areas (%)	-0.44	0.23	-0.06	0.90
GDP per capita	··	··	-0.01	0.95
Female (%)	··	··	-0.19	0.02
≥ junior mid sch educ (%)	··	··	-0.28	0.11
adj. R^2	0.84	··	0.94	··

Note:
Model 1: Population living in urban areas as independent variable, not controlling for covariates except for time dummies
Model 2: Population living in urban areas as independent variable, controlling for covariates except for time dummies

For peer review only - http://bmjopen.bmj.com/site/about/guidelines.xhtml

Table S5. Selected statistics of the variables in the regression model, 2010 and 2016

Variables	Health expenditures per capita (CNY)*		Total health professionals density (per 1,000 population)		Urban-rural ratio in total health professionals density		GDP per capita (CNY)*		Living in urban areas (%)		Female (%)		Received at least middle school education (%)	
Province	2010	2016	2010	2016	2010	2016	2010	2016	2010	2016	2010	2016	2010	2016
Anhui	1210·5	2652·2	3·1	4·7	2·4	2·3	20888·0	39561·0	44.8%	52.0%	49·2%	48·7%	62·8%	65·5%
Beijing	4147·2	9429·7	13·6	10·8	1·9	··	73856·0	118198·0	86.2%	86.5%	48·4%	48·6%	87·6%	88·6%
Chongqing	1501·0	3492·2	3·4	5·9	1·4	2·0	27596·0	58502·0	55.0%	62.6%	49·4%	49·2%	61·0%	64·1%
Fujian	1280·1	3226·8	4·1	5·7	2·8	2·5	40025·0	74707·0	58.1%	63.6%	48·6%	49·1%	63·0%	61·7%
Gansu	1153·9	2889·2	3·7	5·2	2·0	2·2	16113·0	27643·0	37.2%	44.7%	48·9%	49·3%	56·5%	57·9%
Guangdong	1445·9	3812·5	5·3	6·0	3·2	3·2	44736·0	74016·0	66.5%	69.2%	47·8%	46·9%	73·2%	74·3%
Guangxi	1116·9	2557·0	3·6	6·0	2·2	2·2	20219·0	38027·0	41.8%	48.1%	48·0%	48·0%	62·5%	67·4%
Guizhou	946·6	2472·4	2·5	5·8	4·2	3·9	13119·0	33246·0	35.0%	44.2%	48·3%	48·4%	48·8%	54·6%
Hainan	1193·0	3306·8	4·4	6·3	2·3	3·3	23831·0	44347·0	50.5%	56.8%	47·4%	47·3%	72·1%	73·0%
Hebei	1253·8	2710·6	4·0	5·3	3·2	2·7	28668·0	43062·0	45.6%	53.3%	49·3%	48·9%	69·9%	69·7%
Heilongjiang	1580·2	3133·4	5·0	5·8	2·3	2·3	27076·0	40432·0	56.5%	59.2%	49·2%	49·5%	73·0%	73·3%
Henan	1134·0	2594·0	3·5	5·7	3·0	3·2	24446·0	42575·0	40.6%	48.5%	49·5%	49·0%	68·2%	69·8%
Hubei	1191·1	3270·6	4·2	6·5	2·1	2·2	27906·0	55665·0	51.8%	58.1%	48·6%	48·7%	69·8%	70·5%
Hunan	1042·1	2821.0	3·8	5·8	2·9	2·9	24719·0	46382·0	45.1%	52.8%	48·6%	48·9%	67·1%	71·2%

**Inner Mongolia**	1767·5	3599·7	5·1	6·8	2·7	2·6	47347·0	72064·0	56.6%	61.2%	48·1%	49·5%	69·9%	73·3%
**Jiangsu**	1566·0	4200·2	4·4	6·5	2·0	2·1	52840·0	96887·0	61.9%	67.7%	49·6%	49·6%	70·9%	71·5%
**Jiangxi**	992·0	2374·8	5·1	6·1	1·8	2·1	21253·0	40400·0	45.7%	53.1%	48·2%	48·0%	65·6%	64·1%
**Jilin**	1653·9	3501·19	5·5	6·3	2·5	2·9	31599·0	53868·0	53.4%	56.0%	49·3%	49·2%	69·8%	73·6%
**Liaoning**	1765·9	3390·9	4·7	6·6	3·0	2·6	42355·0	50791·0	64.0%	67.4%	49·4%	49·5%	75·5%	78·3%
**Ningxia**	1190·1	3730·5	4·5	6·2	4·4	5·4	26860·0	47194·0	49.8%	56.3%	48·8%	48·4%	61·3%	66·5%
**Qinghai**	1472·0	4043·1	4·7	7·6	2·0	2·0	24115·0	43531·0	46.3%	51.6%	48·2%	48·6%	49·8%	51·8%
**Shaanxi**	2040·7	3535·7	4·7	6·5	1·7	2·2	27133·0	51015·0	47.3%	55.3%	48·3%	49·5%	69·8%	71·5%
**Shandong**	1403·1	3372·7	9·7	7·4	1·3	1·6	41106·0	68733·0	50.9%	59.0%	49·4%	49·0%	67·5%	69·0%
**Shanghai**	2828·1	7596·0	5·6	6·1	2·6	3·2	76074·0	116562·0	89.3%	87.9%	48·5%	48·6%	83·6%	83·5%
**Shanxi**	1297·5	2650·3	3·4	4·8	2·7	3·0	26283·0	35532·0	49.7%	56.2%	48·6%	48·5%	74·1%	77·5%
**Sichuan**	1019·1	3238·6	3·6	6·0	2·0	2·0	21182·0	40003·0	41.8%	49.2%	49·2%	50·1%	57·2%	58·9%
**Tianjin**	2737·3	5294·2	7·1	6·1	1·4	1·1	72994·0	115053·0	80.5%	82.9%	46·6%	46·6%	80·5%	82·0%
**Tibet**	1472·0	3780·9	3·4	4·5	4·9	4·1	17027·0	35184·0	22.8%	29.6%	48·6%	49·5%	26·4%	29·6%
**Xinjiang**	1676·8	4012·9	5·7	7·1	3·2	2·4	25034·0	40564·0	43.5%	48.3%	48·7%	48·9%	66·1%	65·5%
**Yunnan**	1107·2	2754·1	3·2	5·2	3·2	3·3	15752·0	31093·0	36.8%	45.0%	48·1%	49·5%	48·3%	52·5%
**Zhejiang**	2099·0	4603·8	6·1	7·7	1·8	1·8	51711·0	84916·0	62.3%	67.0%	48·6%	47·9%	64·7%	66·0%

*CNY=Chinese Yuan, exchange rate: 1USD≈6.37CNY (Dec 3, 2020)

Data source: National Statistical Yearbook of China (2011, 2017) and National Health Statistical Yearbook of China (2011,2017)

Checklist of information that should be included in new reports of global health estimates

Item #	Checklist item	Reported on page #
**Objectives and funding**		
**1**	Define the indicator(s), populations (including age, sex, and geographic entities), and time period(s) for which estimates were made.	4
**2**	List the funding sources for the work.	6
**Data Inputs**		
For all data inputs from multiple sources that are synthesized as part of the study:		
**3**	Describe how the data were identified and how the data were accessed.	4 & 9
**4**	Specify the inclusion and exclusion criteria. Identify all ad-hoc exclusions.	4
**5**	Provide information on all included data sources and their main characteristics. For each data source used, report reference information or contact name/institution, population represented, data collection method, year(s) of data collection, sex and age range, diagnostic criteria or measurement method, and sample size, as relevant.	4 & 5
**6**	Identify and describe any categories of input data that have potentially important biases (e.g., based on characteristics listed in item 5).	4 & 5 & 9
For data inputs that contribute to the analysis but were not synthesized as part of the study:		
**7**	Describe and give sources for any other data inputs.	4 & 5 & 9
For all data inputs:		
**8**	Provide all data inputs in a file format from which data can be efficiently extracted (e.g., a spreadsheet rather than a PDF), including all relevant meta-data listed in item 5. For any data inputs that cannot be shared because of ethical or legal reasons, such as third-party ownership, provide a contact name or the name of the institution that retains the right to the data.	9 and appendix
**Data analysis**		
**9**	Provide a conceptual overview of the data analysis method. A diagram may be helpful.	4 & 5
**10**	Provide a detailed description of all steps of the analysis, including mathematical formulae. This description should cover, as relevant, data cleaning, data pre-processing, data adjustments and weighting of data sources, and mathematical or statistical model(s).	4 & 5
**11**	Describe how candidate models were evaluated and how the final model(s) were selected.	4 & 5
**12**	Provide the results of an evaluation of model performance, if done, as well as the results of any relevant sensitivity analysis.	7 & 15 & 22 & 23
**13**	Describe methods for calculating uncertainty of the estimates. State which sources of uncertainty were, and were not, accounted for in the uncertainty analysis.	5
**14**	State how analytic or statistical source code used to generate estimates can be accessed.	5 & 6
**Results and Discussion**		
**15**	Provide published estimates in a file format from which data can be efficiently extracted.	12-15
**16**	Report a quantitative measure of the uncertainty of the estimates (e.g. uncertainty intervals).	6,7,15
**17**	Interpret results in light of existing evidence. If updating a previous set of estimates, describe the reasons for changes in estimates.	6-9
**18**	Discuss limitations of the estimates. Include a discussion of any modelling assumptions or data limitations that affect interpretation of the estimates.	8-9

For peer review only - http://bmjopen.bmj.com/site/about/guidelines.xhtml

This checklist should be used in conjunction with the GATHER statement and Explanation and Elaboration document, found on gather-statement.org

For peer review only

For peer review only - http://bmjopen.bmj.com/site/about/guidelines.xhtml
